# Quantitative phospho-proteomics reveals the Plasmodium merozoite triggers pre-invasion host kinase modification of the red cell cytoskeleton

**DOI:** 10.1038/srep19766

**Published:** 2016-02-02

**Authors:** Elizabeth S. Zuccala, Timothy J. Satchwell, Fiona Angrisano, Yan Hong Tan, Marieangela C. Wilson, Kate J. Heesom, Jake Baum

**Affiliations:** 1 Division of Infection and Immunity, Walter and Eliza Hall Institute of Medical Research, Parkville, Victoria, Australia; 2 Department of Medical Biology, University of Melbourne, Parkville, Victoria, Australia; 3 School of Biochemistry, University of Bristol, Bristol, United Kingdom; 4 Department of Life Sciences, Imperial College London, South Kensington, London, United Kingdom

## Abstract

The invasive blood-stage malaria parasite – the merozoite – induces rapid morphological changes to the target erythrocyte during entry. However, evidence for active molecular changes in the host cell that accompany merozoite invasion is lacking. Here, we use invasion inhibition assays, erythrocyte resealing and high-definition imaging to explore red cell responses during invasion. We show that although merozoite entry does not involve erythrocyte actin reorganisation, it does require ATP to complete the process. Towards dissecting the ATP requirement, we present an in depth quantitative phospho-proteomic analysis of the erythrocyte during each stage of invasion. Specifically, we demonstrate extensive increased phosphorylation of erythrocyte proteins on merozoite attachment, including modification of the cytoskeletal proteins beta-spectrin and PIEZO1. The association with merozoite contact but not active entry demonstrates that parasite-dependent phosphorylation is mediated by host-cell kinase activity. This provides the first evidence that the erythrocyte is stimulated to respond to early invasion events through molecular changes in its membrane architecture.

The blood stage malaria parasite, the merozoite, achieves a remarkable feat in terms of cell biology. In less than two minutes it manages to successfully invade a target erythrocyte, a cell that is not only non-phagocytic, but whose membrane architecture also represents one of the most strong, resilient and flexible cellular structures known^1^. The process of parasite invasion forms a core foundation of malaria disease, which despite major gains in the past decade is still responsible for several hundred million cases and as many as half a million or more deaths globally each year^2^. Given the centrality of the blood stage of infection to disease pathology, there has been intensive interest in understanding the mechanisms by which merozoites attach to and invade red blood cells as a route to develop novel strategies that combat entry and, as such, block malaria disease^3^.

Erythrocyte invasion is a stepwise process that begins when a merozoite forms an initial low-affinity attachment to a red blood cell via any point on its surface. Attachment is associated with erythrocyte membrane deformation and is followed by reorientation, where the merozoite brings its apical tip into contact with the target host cell^4^. An electron dense interface then forms between the two cells, called the tight or moving junction, which co-ordinates parasite-erythrocyte interactions during entry^5^,^6^. As the parasite invades it stimulates formation of a new cellular compartment, the parasitophorous vacuole (PV), inside the erythrocyte within which development proceeds.

Most mechanistic studies of invasion have focused on investigating the contribution of parasite factors that are important for invasion, and in particular on the role of parasite adhesive proteins and the merozoite actomyosin motor, which is responsible for driving the parasite into the host cell (reviewed in^3^). Much of this work has been guided, at least in part, by a prevailing dogma that the erythrocyte is a passive victim to an entirely parasite driven process of membrane remodelling and penetration. This model is based primarily on evidence obtained using a distant relative of the malaria parasites, the apicomplexan parasite *Toxoplasma gondii*^7^,^8^, and fails to adequately consider evidence that the erythrocyte membrane is itself capable of extraordinary reversible deformation. This evidence includes the ability of erythrocytes to undergo continued transit through the narrow microcapillaries and splenic sinusoids of the body^9^, and recent evidence that erythrocyte actin might be more dynamic than previously assumed^10^. Given such observations, the potential involvement of a host-mediated component for regulating membrane and cytoskeletal proteins during parasite invasion becomes increasingly attractive. Recent research has demonstrated host-cell actin filament reorganization and accumulation at the point of entry during *T. gondii* tachyzoite invasion of fibroblasts^11^,^12^,^13^ and during murine malaria parasite, *Plasmodium berghei*, sporozoite invasion of hepatocytes^13^. Moreover, a growing body of genetic studies on the role of *T. gondii* and *Plasmodium* invasion proteins (reviewed in^14^) is beginning to undermine the centrality of parasite factors that were once thought to be essential to invasion, further paving the way for consideration of the erythrocyte as an active participant during merozoite entry.

A critical limitation to prior studies on the host cell contribution to merozoite invasion has been historical difficulties in isolating merozoites at the moment of invasion, in particular those from the most virulent malaria parasite *P. falciparum.* Thus, most work to date has not been able to differentiate host factors involved in invasion from those affecting post-invasion growth and development^15^,^16^,^17^. Nonetheless, there are compelling data that point to a role for a dynamic host cell in invasion (reviewed in^1^). For instance, studies focussed on the requirement of erythrocyte ATP in invasion point to the need for active remodelling of the host erythrocyte to support entry. For example, resealed erythrocytes produced by dialysis are resistant to invasion and/or ring-stage growth up to ~20 hours post-entry^18^. Similarly, erythrocytes dialysed in the presence of the non-hydrolysable ATP analogue AMP-PNP were found to be unable to support either invasion, downstream growth or a combination of the two^15^. Finally, in addition to experimental evidence, recent biophysical modelling of cell-cell interactions during entry suggests erythrocyte membrane wrapping could account for a sizeable portion of the energy requirements for invasion given a localized destabilization of the erythrocyte membrane and with only a relatively small contribution absolutely required from the merozoite itself^19^.

Indeed, the red blood cell possesses several complex pathways for altering the mechanical and biophysical characteristics of its membrane and underlying cytoskeleton, of which protein phosphorylation forms a key component. Among phosphorylation events, several are known to lead to a decrease in membrane stability, such as phosphorylation of protein 4.1 and adducin by protein kinase C (PKC)^20^,^21^, dematin by PKA^22^, band 3 by Syk and Lyn kinases^23^ and casein kinase I and casein kinase II phosphorylation of beta-spectrin^24^,^25^. These changes lend support to the idea that parasite-mediated phosphorylation could underpin erythrocyte alterations required for merozoite entry. Analyses of post-invasion infections have identified an array of erythrocyte proteins that display increases in phosphorylation^26^,^27^,^28^ indicating that malaria parasites likely possess effectors required to remodel the erythrocyte though post-translational modification. Although postulated^16^, evidence that such a process occurs during invasion itself is lacking.

Here, we exploit the ability to achieve synchronised erythrocyte invasion using free and viable *P. falciparum* merozoites^29^ to visualize and quantitatively assess active erythrocyte processes in merozoite invasion. Through a combination of invasion assays and quantitative phospho-proteomics we demonstrate a clear host-erythrocyte kinase response to merozoite stimulus and report novel sites of modification associated with this contact.

## Results

### Merozoite invasion of the erythrocyte requires host cell ATP

**Figure 1:**
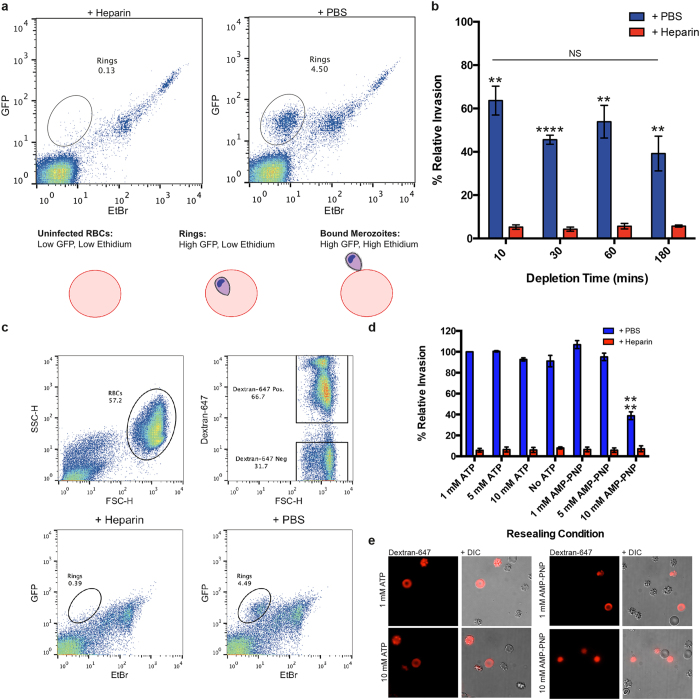
Erythrocyte ATP is required for merozoite invasion. (**a**) Method for quantifying merozoite invasion by flow cytometry. Free GFP-expressing merozoites are added to fresh erythrocytes in a 96-well plate format under agitation for 40 min. Samples are stained with ethidium bromide (EtBr), which labels the parasite nucleus, washed and run on a flow cytometer using a high-throughput plate reader. After gating on erythrocytes, the proportion of red blood cells invaded by merozoites can be quantified. Erythrocytes alone are GFP and EtBr low, while red blood cells with associated parasites and free merozoites are GFP high. EtBr distinguishes between erythrocytes with bound extracellular merozoites, which stain high, and those infected with new rings, which stain low. Here, a new ring-stage parasitemia of 4.5% was achieved, a population that is absent when the invasion assay was performed in the presence of the invasion inhibitor heparin. (**b**) Invasion rate into cells depleted of ATP for different amounts of time, where invasion is expressed as a percentage relative to the new ring parasitemia of control cells that were incubated in PBS alone. **p < 0.05, ***p < 0.01, ****p < 0.0001, NS = non-significant difference, two-tailed unpaired t-test. Graph displays mean +/−SEM. n = 3 in triplicate. (**c**) When free D10-PHG merozoites are added to resealed erythrocytes and stained with EtBr, dextran-Alexa647 allows quantitation of invasion into resealed cells by flow cytometry. (**d**) Invasion rate into erythrocytes resealed in the presence of different concentrations of either ATP or AMP-PNP, where invasion is expressed as a percentage relative to the new ring parasitemia of control cells that were resealed in the presence of 1 mM ATP. ****p < 0.0001, two-tailed unpaired t-test. Graph displays mean +/−SEM. n = 3 in triplicate. (**e**) Live imaging of erythrocytes resealed under different conditions, where dextran-Alexa647 marks successfully resealed cells.

To determine if *P. falciparum* merozoite invasion requires host cell ATP, erythrocytes were pre-treated in ATP depletion medium containing 6 mM iodoacetamide, an inhibitor of glyceraldehyde-3-phosphate dehydrogenase, and 10 mM inosine, which together accelerate ATP consumption and block its production through glycolysis^30^. For instance, incubation of erythrocytes in depletion medium for 1 hour has been shown to irreversibly reduce ATP levels from physiological concentrations (approximately 1 mM–1.5 mM) down to 1–5 uM^30^. Here, erythrocytes depleted of ATP for increasing periods of time were subjected to invasion by free D10-PHG (a cytosolic GFP expressing parasite line^31^) merozoites and invasion was quantified by flow cytometry as previously described^32^ (see schematic, Fig. 1a). Invasion into erythrocytes that had been treated in ATP depletion medium was severely impaired, with invasion reduced to ~40–65% of control levels for all depletion time points. However, increasing the time erythrocytes spent in depletion medium from 10 minutes up to 3 hours did not significantly increase invasion inhibition, indicating the action of the ATP depletion on erythrocyte ability to support invasion was likely rapid (Fig. 1b).

Iodoacetamide, whilst commonly used to inhibit glycolysis, is an alkylating reagent that modifies thiol groups in proteins by S-carboxyamidomethylation, and might thus have effects on erythrocytes beyond achieving irreversible ATP depletion. Notably, whereas incubation of erythrocytes for 3 hours in ATP depletion medium alters the biomechanical properties of their plasma membranes, incubation for 1 hour does not significantly reduce erythrocyte membrane fluctuation amplitudes^33^. To investigate the role of erythrocyte ATP in invasion in a more targeted manner, erythrocyte ATP levels were manipulated by resealing cells with the synthetic non-hydrolysable analogue AMP-PNP^15^. When performed at high haematocrit to reduce loss of cytoplasmic contents, resealed cells retain their ability to support *P. falciparum* invasion and growth, while also enabling the incorporation of fluorescent markers (Supplementary Fig. 1)^34^. Erythrocytes resealed either in the absence of ATP, with increasing amounts of ATP or with increasing concentrations of AMP-PNP, in the presence of fluorescent dextran to label resealed cells, were subjected to merozoite invasion and invasion rates into resealed cells were measured by flow cytometry (Fig. 1c,d). Compared to erythrocytes resealed in the presence of 1 mM ATP (the approximate normal physiological ATP concentration of erythrocytes), erythrocytes resealed with 10 mM AMP-PNP were inhibited from supporting merozoite invasion by ~60% (*p* < 0.0001, two-tailed unpaired t-test). Under all other conditions invasion rates were unaffected. Of note, all resealed erythrocyte populations contained a mixture of normal and mildly echinocytic erythrocytes, with the exception of erythrocytes resealed in the presence of 10 mM AMP-PNP, which all appeared reduced in size and severely echinocytic (Fig. 1e). Although these changes suggest that the relationship between the role of ATP during invasion and its critical role in modulating erythrocyte membrane and cytoskeletal integrity require further dissection, when these data are combined with results from depletion assays, the evidence is supportive of a requirement for erythrocyte ATP in parasite invasion.

### Erythrocyte actin does not polymerize around invading merozoites

**Figure 2:**
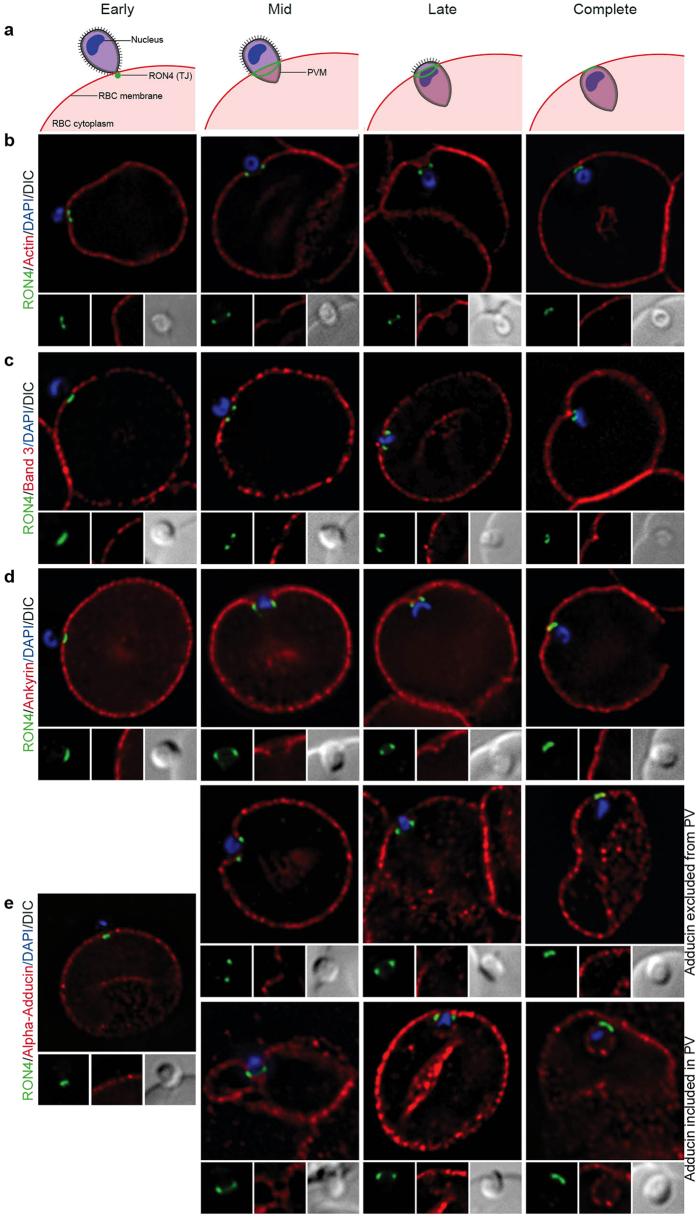
Imaging of the erythrocyte cytoskeleton throughout merozoite invasion. Widefield deconvolution fluorescence imaging of fixed merozoite invasion samples labelled with anti-RON4, DAPI and markers of erythrocyte cytoskeletal proteins. (**a**) Schematic showing how RON4 labelling allows discernment of early, mid, late and complete invasion events. Early in invasion, during apical attachment, RON4 appears as a single point of fluorescence before expanding to form a ring around the invading merozoite as invasion progresses. When imaged in two dimensions, this ring appears as two single points of fluorescence on either side of the merozoite. The right junction marks the boundary between the erythrocyte plasma membrane and the parasitophorous vacuole membrane, and closes at the base of the parasite at the end of invasion. (**b**) Erythrocyte actin, labelled by phalloidin-Alexa594. (**c**,**d**) Band 3, ankyrin and adducin in invasion labelled with specific antibodies. PVM, parasitophorous vacuole membrane; RBC, red blood cell; TJ, tight junction.

We next sought to define what active processes erythrocyte ATP might mediate during parasite entry. Given that host actin filaments accumulation has been described for *T. gondii* tachyzoite and *P. berghei* sporozoite invasion^13^, we aimed to determine whether merozoite invasion resulted in similar levels of actin reorganization in the erythrocyte. Invasion assays with free *P. falciparum* merozoites were performed to capture different stages of invasion and samples analysed by widefield fluorescence microscopy, with RON4 labelling used to track the tight junction (Fig. 2a)^35^. Throughout all stages of invasion, RON4 labelling sat beneath host F-actin (labelled with phalloidin, a phallotoxin that does not bind Apicomplexan actin), and was distributed around the periphery of the cell. By contrast, phalloidin labelling was not apparent at or in front of the tight junction, indicating that actin did not enter the forming parasitophorous vacuole membrane (PVM). Finally, there was no apparent increase in phalloidin staining near the junction, which suggests that erythrocyte actin polymerization is not stimulated around the merozoite during invasion (Fig. 2b).

The labelling pattern of F-actin during invasion mirrored that of the transmembrane protein band 3 (Fig. 2c) and the cytoskeletal linker ankyrin (Fig. 2d), with no obvious reorganisation except for exclusion from the PVM at the tight junction. By contrast, the junctional complex member adducin had two distinct labelling patterns. It was seen either excluded from the nascent PVM or beyond the tight junction in a ring that surrounded the invading parasite (Fig. 2e). Although these observations for adducin are compatible with a model where the erythrocyte responds to invasion through remodelling the molecular architecture of its cytoskeleton, they do not support a situation where host cell actin reorganises around the entering merozoite.

### Treatment of erythrocytes with anti-actin and myosin drugs does not inhibit invasion

It remains possible that only a small fraction of dynamic actin is required for invasion, beyond the resolution limits attained here. Therefore, to investigate whether host actin filament dynamics were functionally required for merozoite entry, invasion drug inhibition assays were performed using cytochalasin D, a membrane permeable fungal metabolite that inhibits actin polymerisation^36^. In addition, to investigate a potential role for erythrocyte myosin during merozoite entry, 2,3-butanedione monoxime (BDM), a broad ATPase inhibitor^37^, and blebbistatin, a more specific inhibitor of non-muscle myosin II^38^, were used.

**Figure 3:**
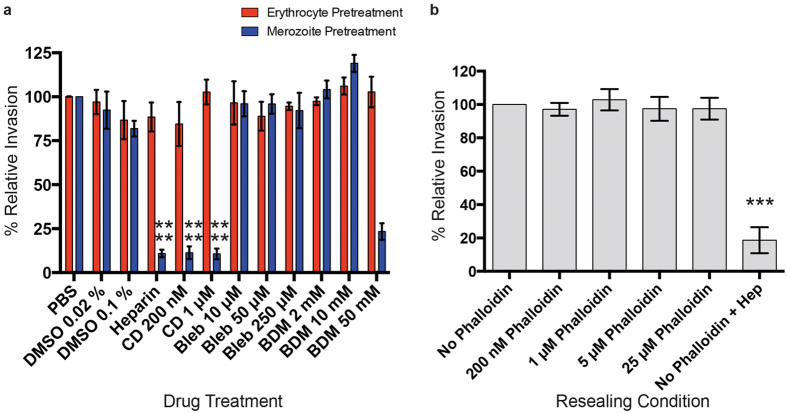
Effect of actin and myosin inhibitors on merozoite invasion. (**a**) Merozoite invasion when either erythrocytes or merozoites were pre-treated with cytochalasin D (CD), blebbistatin (bleb) or BDM. Invasion percentage is expressed relative to the new ring parasitemia of control cells that were incubated in either PBS alone (for DMSO, heparin and myosin inhibitor treatment) or DMSO (for cytochalasin D treatment). ****p < 0.0001, ***p <0.01, two-tailed unpaired t-test. Graph displays mean +/− SEM. n = 3 in triplicate for all samples except BDM at 10 mM and 50 mM, which was performed three times in either duplicate or triplicate. (**b**) Invasion rate into erythrocytes resealed in the presence of different concentrations of phalloidin, where invasion is expressed as a percentage relative to the new ring parasitemia of control cells that were resealed in the absence of phalloidin. Graph displays mean +/− SEM, n = 3 in triplicate.

Prior to invasion, either erythrocytes or merozoites were treated with drug. Pre-treated erythrocytes were washed prior to invasion, however given assay limitations pre-treated merozoite samples retained drugs throughout invasion. No invasion inhibition was observed in the erythrocyte pre-treated samples. Whereas merozoite pre-treatment with 200 nM cytochalasin D or 50 mM BDM strongly inhibited invasion (p < 0.0001, p < 0.0001, p = 0.0002 respectively, two-tailed unpaired t-test), no invasion inhibition was achieved when merozoites were pre-treated with blebbistatin (Fig. 3a). Given that drugs were present during invasion for merozoite pre-treatment samples, it is not wholly possible to distinguish merozoite and host specific drug effects. Moreover, the activities of these compounds are potentially reversible^37^,^39^,^40^. As such, although these data do not support a role for erythrocyte actin dynamics or myosin activity in invasion, such roles cannot be completely discounted.

Therefore, to more specifically target erythrocyte actin, erythrocytes were resealed in the presence of increasing concentrations of phalloidin and fluorescent dextran then subjected to merozoite invasion. Phalloidin only enters erythrocytes upon their permeabilization^41^ and binds with reasonable stability to filaments, preventing their depolymerisation^42^. Even at the highest concentration used (25 μM) phalloidin had no effect on the ability of erythrocytes to support parasite entry (Fig. 3b). Although they do not categorically exclude a role for erythrocyte actin filament dynamics during merozoite invasion, these observations, combined with the washout experiments and imaging data, certainly suggest that host actin filament turnover is not involved in, or is relatively inconsequential to, merozoite entry.

### Quantitative phospho-proteomics of the erythrocyte response to invasion

Having argued against a requirement for host cell ATP-dependent actin and myosin activity for malaria parasite entry, but observing some evidence for cytoskeletal remodelling (in particular adducin), we next sought to test whether early events in merozoite invasion might stimulate erythrocyte-mediated changes in host surface or cytoskeletal protein organisation. The centrality of phosphorylation in regulating erythrocyte membrane and cytoskeletal protein associations is well established^43^. As such, we explored the hypothesis that modulation of host cell phosphorylation of cytoskeletal and membrane proteins provides a means for merozoites to facilitate entry.

Preliminary investigations using total cell lysates of erythrocytes pre-loaded with radioactive phosphate (in the form of H_3_^32^PO_4_) suggested that, relative to non-invaded controls, erythrocytes incubated with merozoites for 2 min exhibited a marked increase in general protein phosphorylation, with a particularly dramatic response seen for of high molecular weight proteins (Supplementary Fig. 2a-b). This approach, however, does not allow identification of the specific target protein or for the high-fidelity quantitation of phosphorylation changes. Thus, to explore this potential change in more detail, we adopted a phospho-proteomic approach to identify host proteins and sites of modification associated with invasion. In addition, we used tandem mass tag (TMT)^44^ labelling of digested peptides in combination with phospho-peptide enrichment, thus enabling quantitative comparison of phospho-peptide abundance of six samples simultaneously assayed by mass spectrometry.

To isolate host cell phosphorylation changes during the early steps of invasion, red blood cells were subjected to rapid merozoite invasion assays (1.5 mins) under three different conditions, either in the presence of heparin, which inhibits firm merozoite attachment and reorientation^45^,^46^, the tight junction-blocking invasion inhibitory peptide R1^35^ or without inhibition, with the aim of isolating phosphorylation events that occur at defined steps during merozoite entry. Each experiment was repeated four times each using different sets of donor erythrocytes, filtered merozoites and mass spectrometry runs. Use of the GFP expressing D10 PHG line in experiments three and four allowed quantitation of representative invasion rates achieved by flow cytometry (Supplementary Fig. 3).

**Figure 4:**
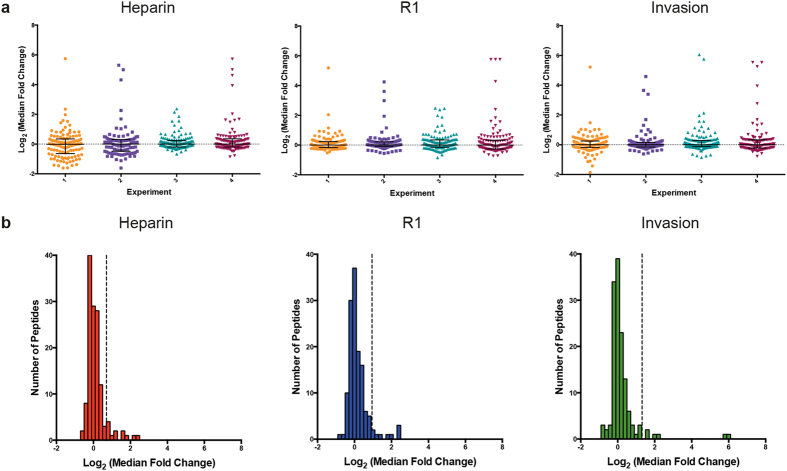
Quantitative phospho-proteomics of merozoite invasion. (**a**) Median fold-change values for high confidence phosphorylated and non-phosphorylated erythrocyte peptides were determined by comparing individual peptide abundances between test (containing added merozoites) and control (erythrocytes alone) samples. Data were transformed by median centering for the three conditions containing either heparin-based invasion inhibition, R1 mediated inhibition or invading merozoites. Bars represent the global median and inter-quartile range for each sample set. (**b**) Histograms from one invasion assay subjected to quantitative phospho-proteomics, displaying the transformed log_2_ (median fold change) value for each phosphorylated and non-phosphorylated erythrocyte peptide detected. Outlier minimums, produced using the ROUT method, are marked with the dashed line. Histogram bin width is 0.2.

Median Peptide fold-change values for high confidence peptides (FDR ≤1%) were produced by comparing the abundance of individual peptides in samples containing erythrocytes and merozoites (numerator) versus erythrocytes alone (denominator) for each of the three conditions tested. In order to directly test the effect of merozoites on erythrocytes, each control sample (denominator) was subjected to the same conditions, including the same drug treatment, as its corresponding invasion sample (numerator). Across the four experiments under all three conditions, most peptide median fold-change values were low and clustered together, while a small number of high fold-change and negative fold-change peptides were detected (Fig. 4a). The summary of all high-confidence erythrocyte peptides detected along with their individual median fold-change values is presented in Supplementary Tables 1–3 and the identity of all high confidence *P. falciparum* peptides collected can be found in Supplementary Table 4. The full set of data collected for all experiments is presented in Supplementary Data 1.

The potentially transient nature of host protein phospho-modification allied to the small proportion of parasites undergoing active invasion within invasion assays provides significant challenges to the identification of sites of specific protein phosphorylation associated with merozoite attachment and/or invasion. The robust regression and outlier removal (ROUT) method was used to identify peptides with fold-change values that likely represented clear responses to merozoite presence, as opposed to those that likely represented natural variation or noise in the data. Outlier peptides with high median fold-change values, representing an increase in phosphorylation in response to merozoites, were detected in all experiments and across all conditions (Fig. 4b and Supplementary Fig. 4). A total of 26 unique outlier phospho-peptides were detected across the four experiments (Supplementary Tables 5–10), with the majority of outlier peptides detected across multiple conditions. Overall, outlier proportions within each assay condition ranged from 3.5% to 9% of erythrocyte peptides (Supplementary Table 9).

The lack of outlier peptide segregation into the different invasion assay conditions is likely explained by experimental conditions. In order to achieve high invasion rates, invasion assays involved applying an excess of merozoites to erythrocytes compared to normal culture conditions^29^. Following our standard protocols, the concentration of merozoites typically produced is approximately 3−4 × 10^5^ merozoites/μL (D. W. Wilson and M. A. Olshina, *personal communication*), yielding a merozoite to erythrocyte ratio in these invasion proteomics assays of between approximately 5:1 and 7:1. In contrast, the rate of erythrocytes invaded in these rapid 1.5 min assays ranged from around 2–4%, such that even in uninhibited invasion samples the vast majority of erythrocytes will have experienced merozoites transiently interacting or binding rather than active invasion events. As a result, phosphorylation events specific to invasion itself (downstream of erythrocyte interactions that occur under heparin treatment) are likely masked in a proteomic assay by the predominant host-parasite interaction occurring in the samples, that of transient and weak merozoite binding.

Additionally, whereas most outlier phospho-peptides were detected in multiple invasion conditions, just over half (54%) were detected in only one of the four proteomics repeats. This variability may be due to differences in merozoite numbers, variability between donor erythrocyte samples, efficiency of phospho-enrichment or chance differences in phospho-peptides detected by the mass spectrometer between experiments, with the abundance of both haemoglobin and merozoite peptides present within the sample likely limiting the depth of sequencing attained. Thus, to increase confidence in assignment of real changes, we took a conservative approach and limited analysis to outlier phospho-peptides that appeared in at least two of the four proteomics experiments (Table 1). Where multiple potential phosphorylated residues are located close to one another in the same peptide, reliable determination of precisely which residues are phosphorylated can be problematic. Shortlisted peptides were interrogated for the accuracy of their phospho-site assignment using the SEQUEST delta Cn score and manual inspection of spectrometry ion fragmentation tables (Table 2). In all cases, except for beta-spectrin p-Y1302, the combination of scoring and manual inspection confirmed the identified phospho-sites. Although this result is strongly suggestive of correct phospho-site assignment, it remains possible that other sites within these peptides are the target of phosphorylation in response to merozoites. In particular, confident site assignments for protein 4.1 phosphorylation at T378 and T740, considered as potentially ambiguous in initial analyses due to their close proximity, led to removal of protein 4.1 from the shortlist as these outlier events each occurred in only a single experiment. Given the inherent limitations in identifying phosphorylated resides by mass spectrometry, however, this does not necessarily preclude protein 4.1 from being a *bone fide* target of phosphorylation during invasion (Supplementary Fig. 6b).

**Figure 5:**
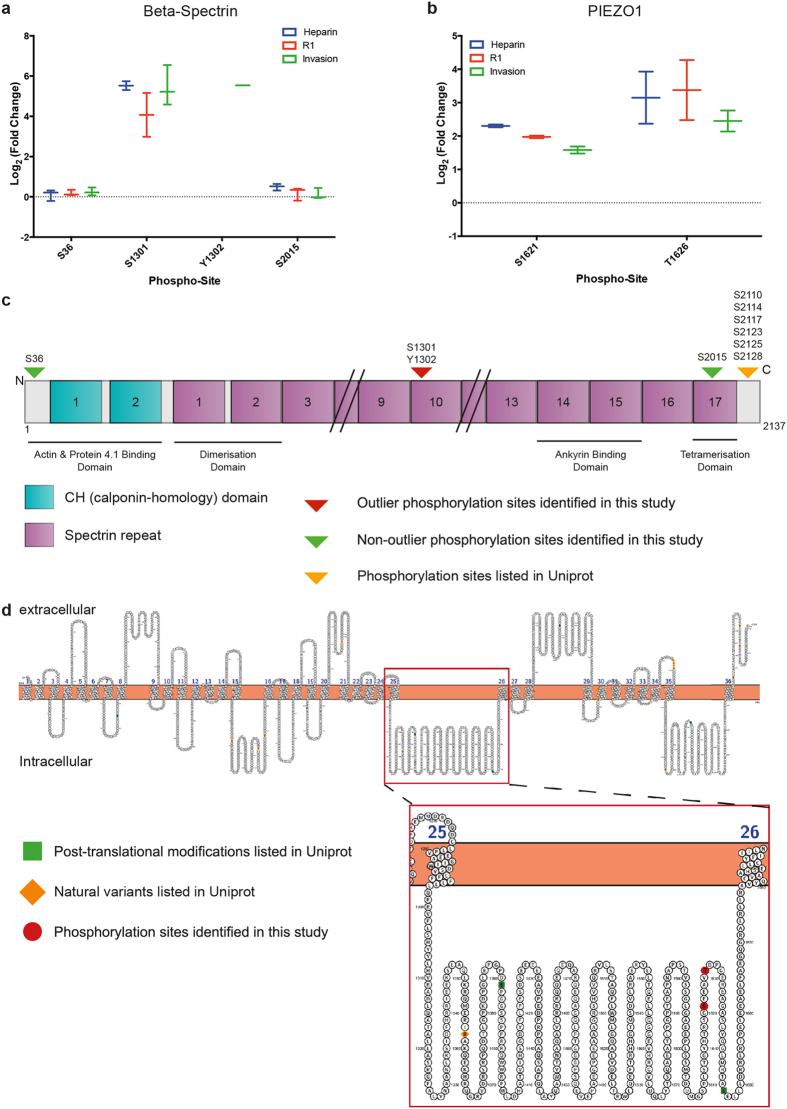
Phosphorylation of beta-spectrin and PIEZO1 in response to merozoites. Median centered individual phospho-peptide fold-change values pooled across the four invasion proteomic experiments for two proteins that contain a shortlisted outlier peptide, (**a**) beta-spectrin and (**b**) PIEZO1. Graphs display box and whisker plots showing the median, maximum and minimum values and inter-quartile range. Schematic representations of (**c**) beta-spectrin and (**d**) PIEZO1. Beta-spectrin phospho-sites identified through quantitative phospho-proteomics of invasion and their locations within the protein relative to key domains and regions of protein-protein interactions. Predicted membrane topology of PIEZO1based on Uniprot-derived consensus features from multiple prediction algorithms. Location of identified outlier phospho-sites is marked in red.

Taking this approach, only peptides from beta-spectrin and PIEZO1 were outliers every time they were detected. The transformed log_2_ (fold-change) values for beta-spectrin peptides corresponding to p-S1301/Y1302 across all three conditions ranged from 3.0 to 6.5 (median = 5.2, SEM = 0.42), indicating a large increase in abundance in response to merozoites, whereas the two other detected phosphorylation sites in the protein (S36 and S2015) displayed minimal changes and were never detected as outliers (Fig. 5a). Although phosphorylation at S1301 has been previously reported in *P. falciparum* infected erythrocytes^47^, to our knowledge phosphorylation at Y1302 has not been identified before. Similarly, both PIEZO1 S1621 (median = 2.0, SEM = 0.14) and T1626 (median = 2.6, SEM = 0.36) displayed consistently high log_2_ (fold- change) values (Fig. 5b), and phosphorylation at both sites has been previously identified (Supplementary Table 11).

The remaining shortlisted phospho-peptides from glycophorin C, eukaryotic translation initiation factor 4B (EIF4B) and ankyrin, although outliers in at least two experiments, were also detected as non-outliers in other experiments (Supplementary Figs 5 and 6). In addition, a number of cytoskeletal and membrane proteins contained phosphorylation events that had low fold changes compared to untreated erythrocytes and were consistently found not to be outliers. These included phospho-peptides from alpha-spectrin, band 3, myosin 9, alpha-adducin and solute carrier family 2 facilitated glucose transporter member 1 (SLC2A1) (Supplementary Figs 7 and 8). The presence of a broad range of protein phosphorylation events that did not increase in abundance in response to invasion provides confidence in the specificity of changes that were seen consistently across experimental conditions.

### Erythrocyte protein phosphorylation in response to merozoites is not due to mechanical stimulation alone

**Figure 6:**
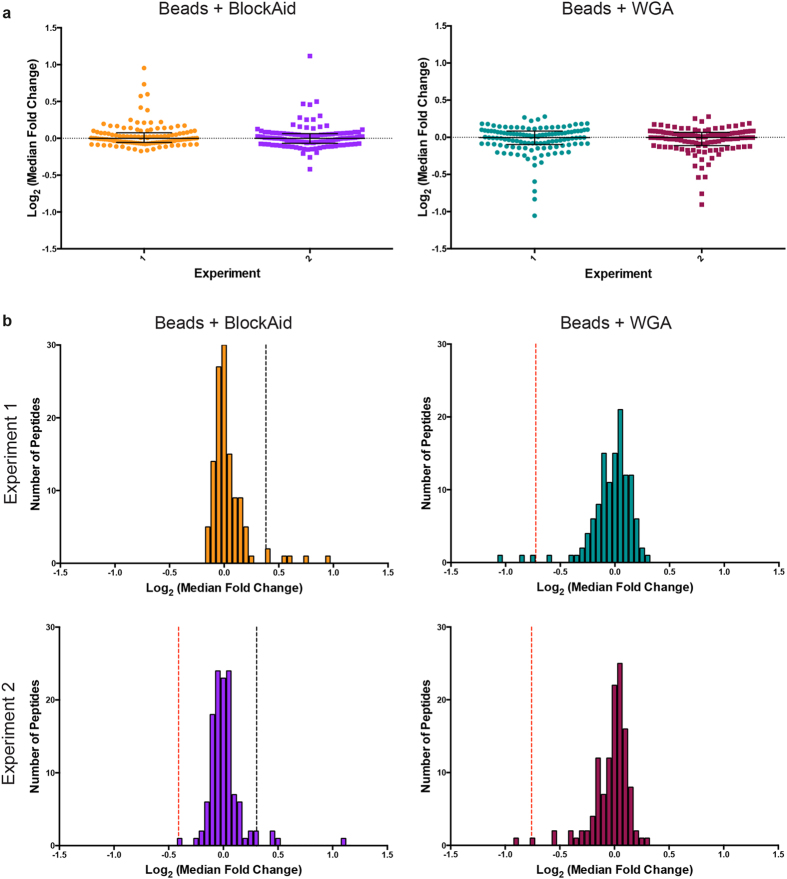
Quantitative phospho-proteomics of erythrocyte response to microbeads. (**a**) Median fold-change values for high confidence phosphorylated and non-phosphorylated erythrocyte peptides were determined by comparing individual peptide abundances between test (containing added microbeads either pre-incubated with WGA or in the presence of BlockAid) and control (erythrocytes alone) samples. Values were transformed by median centering for the two conditions. Bars represent the global median and inter-quartile range for each sample set. (**b**) Histograms of each erythrocyte bead assay subjected to quantitative phospho-proteomics, displaying the transformed log_2_ (median fold change) values for each phosphorylated and non-phosphorylated erythrocyte peptide detected. Outlier boundaries, produced using the ROUT method, are marked with the dashed lines. Black dashed line represents upper boundary, where peptides with values above this line considered outliers, while the red dashed line marks the lower boundary, where peptides with values lower than this value identified as outliers. Histogram bin width is 0.05.

Having demonstrated that erythrocytes respond to the presence of merozoites through phosphorylation, we wanted to exclude the possibility that this was simply due to mechanical stimulation by small particles. This question is particularly relevant given that these merozoite invasion assays are carried out in shaking conditions and noting that one of our top outlier phospho-peptides was from PIEZO1, a mechano-sensitive ion channel. To test whether mechanical force applied by merozoite-sized bodies could stimulate the phosphorylation events detected in invasion assays, we incubated erythrocytes under invasion-assay conditions with fluorescent microspheres of 1μm diameter. Erythrocytes were incubated with shaking alone or in the presence of beads pre-coated with wheat germ agglutinin (WGA), or BlockAid, a commercially available blocking solution. This experiment was repeated twice and the two repeats were processed for quantitative phospho-proteomics and analysed as for merozoite invasion samples. Compared to the range of median fold-change values obtained from our invasion assay proteomics (Fig. 4a), the distribution of values obtained in the two bead assays was more condensed and the maximum values much lower (Fig. 6a). When ROUT-based outlier identification was applied to these datasets (Fig. 6b), compared to the invasion assay experiments a lower proportion of outlier peptides were detected with fold-change values that were also low compared to the values obtained in the invasion proteomics assays (Fig. 6b and Supplementary Tables 9 and 12). A full list of high confidence peptides can be found in Supplemental Data 2.

Shortlisted outlier peptides identified in invasion quantitative-proteomic assays were either absent among the phospho-peptides from the bead experiments, or present as non-outlier peptides (Supplementary Table 13), indicating that these phosphorylation events are likely stimulated specifically by merozoites. Notably, the same outlier beta-spectrin peptide was sequenced in assays both with and without BlockAid, however this peptide was not phosphorylated. For PIEZO1 the phospho-peptide corresponding to p-T1626 was detected as a non-outlier in both conditions. Combined, these data strongly suggest that early events in invasion by the blood stage malaria parasite stimulate ATP-dependent phosphorylation changes in the erythrocyte surface that might be required for successful entry into the human red blood cell.

## Discussion

The proposition that merozoites hijack host pathways of membrane and cytoskeletal remodelling to invade requires, at its base, an erythrocyte capable of carrying out active processes. The possibility that red blood cell ATP is required for merozoite entry was first suggested over two decades ago, however to date no studies have been able to determine whether host ATP was a requirement of invasion itself or downstream growth up to ~20 hours of intracellular development^15^,^18^,^48^. By exploiting the ability to directly and quantitatively assay *P. falciparum* invasion by flow cytometry, we demonstrate that a reduction in erythrocyte ATP indeed leads to a severe defect in merozoite invasion. Although the precise role of ATP in this context remains unclear, given the many morphological, biophysical and biochemical repercussions of ATP depletion, along with potential off-target effects of the treatments used to interfere with ATP, these results nonetheless support a model in which the red blood cell is co-opted to actively participate in its own infection by the merozoite.

Based on our combined imaging and invasion-inhibition analyses, which were unable to demonstrate either the presence of or a reliance on erythrocyte actin filament turnover during *P. falciparum* merozoite entry as observed in other apicomplexan modes of invasion^13^, we conclude that participation of host cell actin in merozoite invasion appears unlikely. Although we cannot completely discount a subtle contribution of a subpopulation of actin filaments (suggested by recent erythrocyte imaging^10^), our data suggest little to no involvement of host actin turnover, and in particular stimulated polymerisation, during malaria parasite entry.

An alternative host-cell involvement during merozoite entry might be the localized destabilization of the erythrocyte cytoskeleton, which would likely increase the potential for membrane wrapping, thereby leading to a lower parasite energy requirement for invasion^19^. This is conceivable through disruption of vertical and lateral linkages of the cytoskeleton rather than through changes in actin filament length or organisation (reviewed in^9^). Intriguingly, the cytoskeletal linker adducin, but not ankyrin, which plays a similar role in the erythrocyte, was observed located beyond the tight junction with a labelling pattern suggestive of a PVM localization. Given that the known binding partners of adducin are either excluded from the PVM (band 3, spectrin, actin and potentially stomatin^9^), or have not been studied in this context (GLUT1), the mechanism of how adducin enters the PVM remains unclear. However, the potential that adducin, unbound to the underlying spectrin/actin cytoskeleton, is present during invasion and flows past the junction introduces the prospect that it may (and by extension, the junctional complex) be a target of cytoskeletal remodelling by the merozoite to achieve changes in host membrane properties required to cause invagination of the erythrocyte plasma membrane and thus PV formation. Live imaging of cytoskeletal components during entry, though currently limited due to a paucity of markers for each protein, might reveal some of the changes that underlie these dynamic events.

Phosphorylation provides a key mechanism by which the erythrocyte is able to regulate the affinity of interactions between membrane and cytoskeletal proteins in order to remodel its structure in response to stimuli. Here, we clearly demonstrate that a large number of red blood cell proteins are phosphorylated in the presence of merozoites, with beta-spectrin and PIEZO1 consistently identified as targets of phosphorylation across biological and technical replicates. The fact that these increases in erythrocyte protein phosphorylation were detected not only in samples where parasites can enter host cells, but also in the presence of the invasion inhibitors R1 and heparin, suggests that erythrocytes respond to early interactions with merozoites. R1 treatment stalls invasion at reorientation^35^, whereas heparin treatment allows weak, transient binding of merozoites to red blood cells and blocks apical reorientation and the events downstream^45^, likely through its interaction with processed forms of MSP1 on the merozoite surface. Moreover, merozoite binding in the presence of heparin allows only minimal deformations to the erythrocyte surface compared to those seen later in invasion^46^. Nonetheless, the phosphorylation events identified by quantitative proteomics appear to be merozoite specific, rather than based on the mechanical stimulation of or sugar group-mediated binding to erythrocytes, raising the question of how the parasite is inducing these changes within the host-cell.

One possible mechanism is through secreted parasite factors, which might interact with the erythrocyte surface prior to active invasion. Alternatively, receptor-ligand interactions between the two cells might explain the observed phosphorylation response. It is currently unclear as to whether key invasion ligands located on the surface of merozoites bind erythrocytes in the presence of heparin, however heparin treatment does inhibit parasites that utilize different invasion pathways at comparable levels^45^, suggesting heparin may not act at the level of EBA and Rh binding. Ligation of complement receptor 1 (CR1) for instance, which is the receptor for PfRh4^49^,^50^, stimulates beta-spectrin phosphorylation and an increase in erythrocyte deformability in a CKII dependent manner^24^. In addition, binding of monoclonal F’ab fragments raised against glycophorin A, the receptor for EBA-175^51^, to the erythrocyte surface alters band 3 mobility in the membrane and increases erythrocyte rigidity^52^,^53^. These data indicate that ligand binding to erythrocyte invasion receptors has the ability to transmit signals into the host cell and alter its membrane and cytoskeletal properties. Whilst different strains of *P. falciparum* invade using different sets of initial receptor-ligand interactions (reviewed in^54^), it is likely that distinct pathways converge at some point to achieve a required alteration in erythrocyte membrane and cytoskeletal properties. Of note here, assays were carried out using a D10 parasite background, which lacks Rh2b and EBA-140 and invades in a chymotrypsin dependent manner^55^. Repetition of quantitative phospho-proteomics using parasite strains that rely on different sets of receptor ligand interactions might shed light on convergent and divergent parasite-initiated host cell signalling pathways.

Is an erythrocyte kinase recruited to prime the host cell for invasion? Since erythrocyte protein phosphorylation was detected prior to the point at which merozoites begin to secrete effectors into the target erythrocyte, it follows that a host cell kinase must be mediating these events. The kinases responsible for phosphorylating the outlier sites identifies through proteomics are unknown. Kinase prediction using NetPhosK is suggestive of a role for CKII and Ca^2+^/calmodulin-dependent protein kinase II (CAMKII) (Supplementary Table 14). Indeed, our data might provide molecular detail of relevance to the reported requirement for an erythrocyte protein with casein kinase activity in either merozoite invasion or early parasite intracellular development^16^. The use of kinase inhibitors in tandem with invasion assays to investigate these hypotheses, whilst attractive, is complicated by the difficulty in attributing the effects of such inhibition as a result of human and *Plasmodium* kinase homology. CAMKII specific inhibitors do not appear to block *P. falciparum* invasion or early ring stage survival and although previous work has indicated that a CKII inhibitor (DRB) does not inhibit invasion, genetic approaches have indicated that the *P. falciparum* CKII homologue is likely essential for blood stage survival and its activity has been implicated in merozoite invasion 56,57,58,59,60. There are currently no CKII inhibitors that could be used to confidently distinguish between host and parasite kinase activity during invasion, however, the divergence between the orthologous might be sufficient to develop inhibitors that specifically target either human or *P. falciparum* CKII in the future 58,61.

It is possible that the observed phosphorylation events may represent responses to merozoite contact or secreted factors with little or no functional consequence, or serve as markers of other invasion-related events in the host cell. More attractively, phosphorylation may function to alter the properties of the cytoskeleton and potentially ‘prime’ the erythrocyte to be amenable to downstream invasion steps, however the consequences of each phosphorylation event identified in this study are currently unknown. Thus far, the only report of phosphorylation at beta-spectrin S1301 was in *P. falciparum* infected erythrocytes^47^. Located in spectrin repeat 10, this phosphorylation site is not located in any of the known beta-spectrin protein-protein interaction domains that form the key linkages within the cytoskeleton (Fig. 5c), making its potential effect on spectrin function difficult to predict. Finally, while the structure and membrane topology of human PIEZO1 have not been confirmed^62^, outlier phospho-sites located at S1621 and T1626 are both located with a predicted intracellular loop of this large multi-transmembrane domain protein (Fig. 5d). The exact function that this mechano-sensitive stretch-induced ion channel plays in normal erythrocyte function remains poorly understood, much less the potential effect of its post-translational modification in response to stimuli. Nevertheless, mutations and disruptions to PIEZO1 have indicated a role in erythrocyte volume regulation, and in other cell types it has been shown to have a role in calcium ion transport (reviewed in^63^). In this light, the prospective involvement of PIEZO1, whether active or incidental, in response to merozoite stimulus is perhaps not surprising.

In summary, we present the first quantitative phospho-proteomic dataset addressing erythrocyte protein modification in response to stimulus by the early events of blood stage malaria parasite entry. We can exclude a significant contribution of host cell actin dynamics during the invasion process whilst demonstrating a host kinase response to the earliest membrane contact with the merozoite through the phosphorylation of prominent erythrocyte membrane and cytoskeletal proteins. It is now clear from this work that the erythrocyte is capable of and indeed does respond actively to attachment and invasion of the *Plasmodium* merozoite. With future development of phospho-specific antibodies to each target protein and development of live imaging probes, we anticipate that this study will inform future efforts focused toward deciphering the potential for host cell signalling and kinase involvement in invasion and to understand the precise membrane and cytoskeletal protein remodelling events that underscore red blood cell responses to malaria parasite invasion.

## Materials and Methods

### Standard parasite tissue culture

*P. falciparum* strains D10 M3′ and D10-PHG^32^ were cultured in complete culture medium, comprised of RPMI 1640 medium (GIBCO) supplemented with 25 mM HEPES, 0.0625% (v/v) gentamycin (Pfizer), 0.2% (v/v) NaHCO_3_ and either 0.4% (w/v) Albumax II (GIBCO) or a combination of 0.2% (w/v) Albumax II and 5% (v/v) heat inactivated human serum from O^+^ donors (complete culture medium) (Australian Red Cross Blood Bank) (Walter and Eliza Hall Human Research Ethics Committee (ethics number 86/17) and an Australian Red Cross Blood Service Agreement (11-09VIC-01)). Cultures were synchronised as previously described using a combination of sorbitol (Sigma) synchronization^64^ and treatment with 20 IU/mL (approximately 153 μg/mL) medicinal grade heparin (porcine mucous, Pfizer, AUST R 49232)^45^.

### Merozoite preparation and standard invasion assay

Unless otherwise specified, free merozoites were isolated and used in standard erythrocyte invasion assays according to published protocols^29^. Assays were incubated at 37 °C for specified times on rotation at either 1,100 rpm (imaging and phosphorylation assays) or 400 rpm (invasion quantitation by flow cytometry).

### Imaging of merozoite invasion

Imaging of invasion followed established methods^35^. For immunofluorescence, primary antibodies used were mouse anti-RON4 1:500 and rabbit anti-RON4 1:250^35^, rabbit anti-alpha adducin 1:1000, rabbit anti -ankyrin 1:1000 (kind gifts from Vann Bennett, Duke University) and rat-anti band 3 clone BRAC18^65^ (kind gift from David Anstee, NHSBT Bristol). Secondary antibodies used were goat anti-mouse Alexa Fluor^®^ 488 (A-11001, ThermoFisher), goat anti-rabbit Alexa Fluor^®^ 488 (A-11008), goat anti rabbit Alexa Fluor^®^ 594 (A-11037) or goat anti-rat Alexa Fluor^®^ 594 (A-11007). Phalloidin Alexa Fluor^®^ 594 1:50 (A12381, ThermoFisher) was used to label erythrocyte F-actin. Samples were mounted in Vectashield (Vector Laboratories, H-1000) containing 0.1 ng/μL 4′,6-diamidino- 2-phenylindole (DAPI, Life technologies, D3571) and widefield deconvolution imaging performed using a DeltaVision Elite equipped with a TrueLight illumination system, Photometrics CoolSnap HQ2 camera, 100 × 1.40 NA objective and SoftWoRx v6 software.

### Production of assays for quantitation of invasion by flow cytometry

Where indicated, erythrocytes were pre-treated with the actin and myosin inhibitors cytochalasin D (C2618, Sigma), blebbistatin (B0560, Sigma) and 2,3-Butanedione monoxime (BDM, B0753, Sigma) prior to merozoite invasion for 1 h at 37 °C, and washed two times. Filtered merozoites were then added to treated erythrocytes and invasion assays completed as published^32^.

To deplete erythrocytes of ATP prior to invasion, erythrocytes were washed three times in PBS and incubated at 8% haematocrit in either PBS alone or depletion media containing 6 mM iodoacetamide (I6125, Sigma) and 10 mM inosine (I4125, Sigma) in PBS for between 10 min and 3 h at 37 °C in a humidifying oven on rotation. Treated cells were then washed three times in incomplete culture medium and used in merozoite invasion assays.

### Invasion assays for proteomics

Quantitative proteomic experiments were performed using lysates of erythrocytes undergoing active merozoite invasion or in the presence of either 40 IU/mL heparin (Pfizer) or 100 μg/mL R1 peptide (GL Biochem), where invasion was allowed to continue for 1.5 min to enrich for mid-invasion events. As such, invasion rates obtained tended to be lower than for standard flow cytometry based assays (above), which allow merozoite-erythrocyte interaction for 30–40 mins. For assays where D10-PHG parasites were used, a proportion of the sample was used for evaluation of invasion rate by flow cytometry. The remaining sample was then rapidly centrifuged (676 × g, 30 sec), supernatant containing media and unbound merozoites removed and pellets snap frozen on dry ice to stop invasion.

For control microbead assays, erythrocytes were isolated from O^+^ platelet apheresis blood waste (NHSBT, Bristol, UK) from healthy donors with informed consent for research use in accordance with the Declaration of Helsinki and approval from the Local Research Ethics Committee (REC 12/SW/0199). Fluospheres of 1 μm diameter (Life Technologies, F8852) (0.67% solution) were incubated with BlockAid solution (Life Technologies, B10710) or wheat germ agglutinin (Sigma, L9640) at 50μg/mL for 1 h with rotation at room temperature. Beads were washed once in complete culture media, resuspended in complete culture media at a concentration of 3.6 × 10^6^/μL and sonicated for 1 min in a sonicating water bath. A concentration of 3.6 × 10^6^/μL microbeads was selected to mimic standard merozoite invasion assay conditions, which typically involve incubation of erythrocytes with filtered merozoites at between 3 × 10^6^/μL and 4 × 10^6^/μL (Danny Wilson and Maya Olshina, Walter and Eliza Hall Institute of Medical Research, personal communication). Assays were performed as described for merozoite invasion assays.

### Erythrocyte resealing

O^+^ erythrocytes supplied in equal amounts from two different donors were washed three times in incomplete culture medium, and 100 μL of packed erythrocytes (pRBCs) used per assay. To lyse, pRBCs were resuspended in 100 μL of lysis buffer at 4 °C (5mM K_2_HPO_4_, 1 mM ATP, pH 7.4) and incubated on ice, with periodical agitation, for 10 min. To reseal, 20 μL of resealing buffer (475 mM KoAc, 25 mM Na_2_HPO_4_, 25 mM MgCl_2_, 237.5 mM KCl, 1 mM ATP, 5 mM GTP, 1.5 mM DTT, pH 7) was added to the lysing erythrocytes and each sample incubated at 37 °C on rotation for 1 h in a humidifying oven. Samples were washed three times in incomplete culture medium. For resealing of membrane impermeable compounds into erythrocytes, the desired compounds were included in the lysis buffer. To label resealed erythrocytes, dextran-Alexa Fluor^®^ 647 10,000 MW (D-22914, ThermoFisher) was included in lysis buffer resulting in a final concentration of 125 μg/mL. Phalloidin (P3457) was supplied by ThermoFisher. For ATP studies, various concentrations of either ATP (adenosine 5′-triphosphate disodium salt, A6559, Sigma) or AMP-PNP (adenosine 5′ –(β,γ-imido)triphosphate tetralithium salt hydrate, A2647, Sigma), were included in both the lysis and resealing buffers, with ATP or analogue buffer concentrations ranging from 1 mM to 10 mM in equal amounts of solvent (DDW).

### Quantitative phospho-proteomics

#### Sample Preparation

Samples were lysed for 10 min on ice in lysis buffer containing 20 mM Tris-HCl pH 8.0, 137 mM NaCl, 10 mM EDTA, 100 mM NaF, 1% (v/v) Nonidet P-40, 10 mM Na_3_VO_4_, 2 mM PMSF, 1% (v/v) protease inhibitor cocktail set V (Merck Millipore) and 1% (v/v) phosphatase inhibitor cocktail set IV (Merck Millipore). Total protein concentration of lysates was quantified using a DC Protein Assay (BioRad) and 100 μg of each sample labelled using TMTsixplex reagents according to manufacturer’s instructions (Thermo Scientific). The labelled samples were pooled and then subjected to phospho-peptide enrichment using a TiO_2_-based enrichment kit, according to the manufacturer’s instructions (Pierce). The phospho-enriched sample was then analysed by RP nano-LC MSMS using an LTQ-Orbitrap Velos mass spectrometer.

#### Nano-LC Mass Spectromerty

Each phospho-enriched sample was fractionated using an Ultimate 3000 nanoHPLC system in line with an LTQ-Orbitrap Velos mass spectrometer (Thermo Scientific). In brief, peptides in 1% (v/v) formic acid were injected onto an Acclaim PepMap C18 nano-trap column (Thermo Scientific). After washing with 0.5% (v/v) acetonitrile 0.1% (v/v) formic acid peptides were resolved on a 250 mm × 75 μm Acclaim PepMap C18 reverse phase analytical column (Thermo Scientific) over a 150 min organic gradient, using seven gradient segments (1–6% solvent A over 1 min, 6–15% B over 58 min, 15–32% B over 58 min, 32–40% B over 3 min, 40–90% B over 1 min, held at 90% B for 6 min and then reduced to 1% B over 1min) with a flow rate of 300 nL/min. Solvent A was 0.1% (v/v) formic acid and Solvent B was aqueous 80% (v/v) acetonitrile in 0.1% (v/v) formic acid. Peptides were ionized by nano-electrospray ionization at 2.1 kV and a capillary temperature of 250 °C.

Tandem mass spectra were acquired using an LTQ- Orbitrap Velos mass spectrometer controlled by Xcalibur 2.1 software (Thermo Scientific) and operated in data-dependent acquisition mode. The Orbitrap was set to analyse the survey scans at 60,000 resolution (at m/z 400) in the mass range m/z 300 to 1800 and the top 10 multiply charged ions in each duty cycle selected for MS/MS fragmentation using higher-energy collisional dissociation (HCD) with a normalised collision energy of 45%, activation time of 0.1 ms and at a resolution of 7500 within the Orbitrap. Charge state filtering, where unassigned precursor ions were not selected for fragmentation, and dynamic exclusion (repeat count, 1; repeat duration, 30 s; exclusion list size, 500) was used.

#### Data Processing

The raw data files were processed using Proteome Discoverer software v1.2 (Thermo Scientific) and searched against a combined database consisting of the UniProt Human and *P. falciparum* 3D7 databases using the SEQUEST algorithm. Peptide precursor mass tolerance was set at 10 ppm, and MS/MS tolerance was set at 20 mmu. Search criteria included carbamidomethylation of cysteine (+57.0214) and the addition of the TMT 6Plex mass tag (+229.163) to peptide N-termini and lysine as fixed modifications and oxidation of methionine (+15.9949) and phosphorylation of serine, threonine and tyrosine (79.966Da) as variable modifications. Searches were performed with full tryptic digestion and a maximum of 1 missed cleavage was allowed. The reverse database search option was enabled and all peptide data was filtered to satisfy false discovery rate (FDR) of 5%. The Proteome Discoverer software generates a reverse “decoy” database from the same protein database used for the search and any peptides passing the initial filtering parameters that were derived from this decoy database are defined as false positive identifications. The minimum cross-correlation factor (Xcorr) filter was readjusted for each individual charge state separately to optimally meet the predetermined target FDR of 5% based on the number of random false positive matches from the reverse decoy database. Thus each data set has its own passing parameters. Quantitation was done using a peak integration window tolerance of 0.0075 Da with the integration method set as the most confident centroid.

#### Data Analysis

Quantitative peptide fold-change data was manually curated using Excel v14.4.1 to select peptides of human origin with a high confidence measure (FDR ≤ 1%). Peptides derived from known serum proteins, based on localisation data from the Uniprot database, were excluded from further analysis. Where multiple spectra were obtained for the same peptide within an experiment, the median fold-change of the reads was taken. Peptide medians were then transformed to log base 2 and median centered to adjust for loading, a linear transformation commonly used in microarray analyses^66^, by subtracting the global median of the dataset from each individual median peptide fold-change value. To identify outliers, representing fold-change values that fall outside the assumed normal variation in the data, the ROUT method was employed^67^ using Prism v6. This method assumes a dataset follows a normal distribution and uses non-linear regression to identify outliers with a false discovery rate of 1%^67^. ROUT analysis was applied to median fold-change values prior to logarithmic transformation.

The confidence of phospho-site localisations within individual outlier peptides was assessed using Scaffold v4 (Proteome Software) to calculate the SEQUEST delta Cn score, which is a measure of the difference in the top two peptide sequence correlation scores (Xcorr), where a score of > 0.1 is considered a confident modified site assignment^68^,^69^,^70^. Visualization of individual peptide spectra and their corresponding ion fragmentation tables was also performed using Scaffold v4 to manually confirm confidence measures provided by the delta Cn scores. Peptide modification sites were mapped within their respective proteins based on protein sequences obtained from the Uniprot database using EditSeq v10.1.2 (DNASTAR Lasergene). Data presentation was carried out using Prism v6.

Data collected for peptides of *P. falciparum* origin was filtered using Excel v14.4.1 to exclude medium confidence peptides (1% ≤ FDR ≤ 5%) and retain only high confidence peptides (FDR ≤ 1%).

## Additional Information

**How to cite this article**: Zuccala, E. S. *et al.* Quantitative phospho-proteomics reveals the Plasmodium merozoite triggers pre-invasion host kinase modification of the red cell cytoskeleton. *Sci. Rep.* **6**, 19766; doi: 10.1038/srep19766 (2016).

## Supplementary Material

Supplementary Information

Supplementary Data 1

Supplementary Data 2

## Figures and Tables

**Table 1:** Shortlisted outlier phospho-peptides.

Protein	Peptide	Mapped	Experiment 1			Experiment 2			Experiment 3			Experiment 4		
			Hep.	R1	Inv.	Hep.	R1	Inv.	Hep.	R1	Inv.	Hep.	R1	Inv.
Beta-spectrin	LLTSQDVsYDEAR	S1301	Y	Y	Y	Y	Y	Y	ND	ND	Y	ND	ND	ND
Beta-spectrin	LLTSQDVSyDEAR	Y1302	ND	ND	ND	ND	ND	ND	ND	ND	ND	ND	ND	Y
PIEZO1	SGsEEAVTDPGER	S1621	Y	Y	Y	Y	Y	Y	ND	ND	ND	ND	ND	ND
PIEZO1	SGSEEAVtDPGER	T1626	ND	ND	ND	ND	ND	ND	Y	Y	Y	Y	Y	Y
Glycophorin C	GTEFAEsADAALQGDPALQDAGDSSR	S104	N	N	N	ND	ND	ND	Y	Y	Y	Y	Y	Y
EIF4B	SQSSDTEQQsPTSGGGK	S504	N	N	N	ND	ND	ND	Y	Y	Y	Y	Y	Y
EIF4B	SQSSDtEQQSPTSGGGK	T500	ND	ND	ND	ND	ND	ND	ND	ND	ND	N	N	N
Protein 4.1	TQTVtISDNANAVK	T738	ND	ND	ND	ND	ND	ND	ND	ND	Y	ND	ND	ND
Protein 4.1	TQTVTIsDNANAVK	S740	ND	ND	ND	ND	ND	ND	ND	ND	ND	Y	Y	Y
Ankyrin	RQDDATGAGQDsENEVSLVSGHQR/GDDATGAGQDsENEVSLVSGHQR	S1666	N	N	N	N	N	N	Y	N	N	Y	Y	Y
Ankyrin	ITHsPtVSQVTER	S1686, T1688	N	Y	Y	N	N	N	N	N	N	ND	ND	ND
Ankyrin	ITHSPtVsQVTER	T1688, S1690	ND	ND	ND	N	N	N	ND	ND	ND	Y	N	N
Glucose 1,6-bisphosphate synthase	AVAGVmITAsHNR	M171, S175	ND	ND	ND	ND	ND	ND	Y	N	N	Y	Y	N

13 unique phospho‐peptides were detected as outliers in at least two experiments. Since confident assignment of phospho‐sites in mass spectrometry is often difficult when multiple possible phosphorylated residues are located in the same peptide, in cases where two identified phospho-sites were close to one another on the same peptide, such as for beta spectrin, the data for both peptides was considered collectively. Abbreviations: Y, yes peptide is an outlier; N, no peptide is not an outlier; ND, peptide not detected; Mapped, corresponding phospho‐site in the protein; Hep, invasion assay conducted in the presence of heparin; R1, assay used R1 peptide; Inv. uninhibited invasion assay. Modifications on S, T and Y residues are phosphorylation. Modification of M residues is oxidation.

**Table 2:** Confidence measures of phospho-site assignment for shortlisted outlier phospho-peptides.

Protein	Peptide	Average SEQUEST delta Cn Score				
		Mod.	Exp.1	Exp. 2	Exp. 3	Exp. 4
Beta Spectrin	LLTSQDVsYDEAR	S8	0.58	0.56	0.09**	ND
Beta Spectrin	LLTSQDVSyDEAR	Y9	ND	ND	ND	0.00*
PIEZO1	SGsEEAVTDPGER	S3	0.63	0.52	ND	ND
PIEZO1	SGSEEAVtDPGER	T8	ND	ND	0.29	0.14
Glycophorin C	GTEFAEsADAALQGDPALQDAGDSSR	S7	0.32	ND	0.32	0.35
Protein 4.1	TQTVtISDNANAVK	T5	ND	ND	0.05**	ND
Protein 4.1	TQTVTIsDNANAVK	S7	ND	ND	ND	0.02**
EIF4B Protein	SQSSDTEQQsPTSGGGK	S10	0.10	ND	0.14	ND
EIF4B Protein	SQSSDtEQQSPTSGGGK	T6	ND	ND	ND	0.11
Ankyrin 1	RQDDATGAGQDsENEVSLVSGHQR/GDDATGAGQDsENEVSLVSGHQR	S12/S11	0.56	0.58	0.35	0.32
Ankyrin 1	ITHsPtVSQVTER	S4, T6	0.59	0.52	0.11	ND
Ankyrin 1	ITHSPtVsQVTER	T6, S8	ND	0.15	ND	0.01**

For each experiment, the average SEQUEST delta Cn score was calculated for outlier phospho-peptides and those peptides with the same sequence and an alternative closely located phosphorylated residue, where a score of > 0.1 is considered a confident modified site assignment. *Manual inspection of peptide fragmentation ions indicated site assignment is ambiguous. **Manual inspection reveals good evidence for selected phospho-site. Abbreviations: Mod, modification site within peptide; Exp., experiment; ND, peptide not detected.

## References

[b1] Zuccala E. S. & Baum J. Cytoskeletal and membrane remodelling during malaria parasite invasion of the human erythrocyte. Br J Haematol 154, 680–689 (2011). 21718279 10.1111/j.1365-2141.2011.08766.x

[b2] White N. J. *et al.* Malaria. Lancet 383, 723–735 (2014). 23953767 10.1016/S0140-6736(13)60024-0

[b3] Cowman A. F., Berry D. & Baum J. The cellular and molecular basis for malaria parasite invasion of the human red blood cell. J Cell Biol 198, 961–971 (2012). 22986493 10.1083/jcb.201206112 PMC3444787

[b4] Dvorak J. A., Miller L. H., Whitehouse W. C. & Shiroishi T. Invasion of erythrocytes by malaria merozoites. Science 187, 748–750 (1975). 803712 10.1126/science.803712

[b5] Aikawa M., Miller L. H., Johnson J. & Rabbege J. Erythrocyte entry by malarial parasites. A moving junction between erythrocyte and parasite. J Cell Biol 77, 72–82 (1978). 96121 10.1083/jcb.77.1.72 PMC2110026

[b6] Bannister L. H., Butcher G. A., Dennis E. D. & Mitchell G. H. Structure and invasive behaviour of Plasmodium knowlesi merozoites *in vitro*. Parasitology 71, 483–491 (1975). 1202413 10.1017/s0031182000047247

[b7] Dobrowolski J. M. & Sibley L. D. Toxoplasma invasion of mammalian cells is powered by the actin cytoskeleton of the parasite. Cell 84, 933–939 (1996). 8601316 10.1016/s0092-8674(00)81071-5

[b8] Morisaki J. H., Heuser J. E. & Sibley L. D. Invasion of Toxoplasma gondii occurs by active penetration of the host cell. J Cell Sci 108, 2457–2464 (1995). 7673360 10.1242/jcs.108.6.2457

[b9] Mohandas N. & Chasis J. A. Red blood cell deformability, membrane material properties and shape: regulation by transmembrane, skeletal and cytosolic proteins and lipids. Semin Hematol 30, 171–192 (1993). 8211222

[b10] Gokhin D. S. *et al.* Dynamic actin filaments control the mechanical behavior of the human red blood cell membrane. Mol Biol Cell 26, 1699–1710 (2015). 25717184 10.1091/mbc.E14-12-1583 PMC4436781

[b11] Bichet M. *et al.* The toxoplasma-host cell junction is anchored to the cell cortex to sustain parasite invasive force. BMC Biol 12, 773 (2014). 25551479 10.1186/s12915-014-0108-y PMC4316648

[b12] Delorme-Walker V. *et al.* Toxofilin upregulates the host cortical actin cytoskeleton dynamics, facilitating Toxoplasma invasion. J Cell Sci 125, 4333–4342 (2012). 22641695 10.1242/jcs.103648 PMC3516439

[b13] Gonzalez V. *et al.* Host cell entry by apicomplexa parasites requires actin polymerization in the host cell. Cell Host Microbe 5, 259–272 (2009). 19286135 10.1016/j.chom.2009.01.011

[b14] Bargieri D. *et al.* Host cell invasion by apicomplexan parasites: the junction conundrum. PLoS Pathog 10, e1004273 (2014). 25232721 10.1371/journal.ppat.1004273 PMC4169498

[b15] Dluzewski A. R., Rangachari K., Wilson R. J. & Gratzer W. B. A cytoplasmic requirement of red cells for invasion by malarial parasites. Mol Biochem Parasitol 9, 145–160 (1983). 6321983 10.1016/0166-6851(83)90106-8

[b16] Rangachari K., Dluzewski A., Wilson R. J. & Gratzer W. B. Control of malarial invasion by phosphorylation of the host cell membrane cytoskeleton. Nature 324, 364–365 (1986). 3537806 10.1038/324364a0

[b17] Rangachari K., Dluzewski A. R., Wilson R. J. & Gratzer W. B. Cytoplasmic factor required for entry of malaria parasites into RBCs. Blood 70, 77–82 (1987). 3297207

[b18] Olson J. A. & Kilejian A. Involvement of spectrin and ATP in infection of resealed erythrocyte ghosts by the human malarial parasite, Plasmodium falciparum. J Cell Biol 95, 757–762 (1982). 6759513 10.1083/jcb.95.3.757 PMC2112918

[b19] Dasgupta S. *et al.* Membrane-wrapping contributions to malaria parasite invasion of the human erythrocyte. Biophys J 107, 43–54 (2014). 24988340 10.1016/j.bpj.2014.05.024 PMC4184798

[b20] Manno S., Takakuwa Y. & Mohandas N. Modulation of erythrocyte membrane mechanical function by protein 4.1 phosphorylation. J Biol Chem 280, 7581–7587 (2005). 15611095 10.1074/jbc.M410650200

[b21] Matsuoka Y., Li X. & Bennett V. Adducin is an *in vivo* substrate for protein kinase C: phosphorylation in the MARCKS-related domain inhibits activity in promoting spectrin-actin complexes and occurs in many cells, including dendritic spines of neurons. J Cell Biol 142, 485–497 (1998). 9679146 10.1083/jcb.142.2.485 PMC2133059

[b22] Koshino I., Mohandas N. & Takakuwa Y. Identification of a novel role for dematin in regulating red cell membrane function by modulating spectrin-actin interaction. J Biol Chem 287, 35244–35250 (2012). 22927433 10.1074/jbc.M111.305441 PMC3471709

[b23] Ferru E. *et al.* Regulation of membrane-cytoskeletal interactions by tyrosine phosphorylation of erythrocyte band 3. Blood 117, 5998–6006 (2011). 21474668 10.1182/blood-2010-11-317024 PMC3112043

[b24] Glodek A. M. *et al.* Ligation of complement receptor 1 increases erythrocyte membrane deformability. Blood 116, 6063–6071 (2010). 20861458 10.1182/blood-2010-04-273904 PMC3031392

[b25] Manno S., Takakuwa Y., Nagao K. & Mohandas N. Modulation of erythrocyte membrane mechanical function by beta-spectrin phosphorylation and dephosphorylation. J Biol Chem 270, 5659–5665 (1995). 7890688 10.1074/jbc.270.10.5659

[b26] Murray M. C. & Perkins M. E. Phosphorylation of erythrocyte membrane and cytoskeleton proteins in cells infected with Plasmodium falciparum. Mol Biochem Parasitol 34, 229–236 (1989). 2525229 10.1016/0166-6851(89)90051-0

[b27] Pantaleo A. *et al.* Analysis of changes in tyrosine and serine phosphorylation of red cell membrane proteins induced by P. falciparum growth. Proteomics 10, 3469–3479 (2010). 20799346 10.1002/pmic.201000269

[b28] Wu Y. *et al.* Identification of phosphorylated proteins in erythrocytes infected by the human malaria parasite Plasmodium falciparum. Malar J 8, 105 (2009). 19450262 10.1186/1475-2875-8-105 PMC2696463

[b29] Boyle M. J. *et al.* Isolation of viable Plasmodium falciparum merozoites to define erythrocyte invasion events and advance vaccine and drug development. Proc Natl Acad Sci USA 107, 14378–14383 (2010). 20660744 10.1073/pnas.1009198107 PMC2922570

[b30] Lew V. L. & Garcia-Sancho J. Measurement and control of intracellular calcium in intact red cells. Methods Enzymol 173, 100–112 (1989). 2528668 10.1016/s0076-6879(89)73008-1

[b31] Wilson D. W., Crabb B. S. & Beeson J. G. Development of fluorescent Plasmodium falciparum for *in vitro* growth inhibition assays. Malar J 9, 152 (2010). 20525251 10.1186/1475-2875-9-152 PMC2891815

[b32] Wilson D. W., Langer C., Goodman C. D., McFadden G. I. & Beeson J. G. Defining the timing of action of antimalarial drugs against Plasmodium falciparum. Antimicrob Agents Chemother 57, 1455–1467 (2013). 23318799 10.1128/AAC.01881-12 PMC3591904

[b33] Betz T., Lenz M., Joanny J.-F. & Sykes C. ATP-dependent mechanics of red blood cells. Proc Natl Acad Sci USA 106, 15320–15325 (2009). 19717437 10.1073/pnas.0904614106 PMC2741249

[b34] Abu Bakar N., Klonis N., Hanssen E., Chan C. & Tilley L. Digestive-vacuole genesis and endocytic processes in the early intraerythrocytic stages of Plasmodium falciparum. J Cell Sci 123, 441–450 (2010). 20067995 10.1242/jcs.061499

[b35] Riglar D. T. *et al.* Super-Resolution Dissection of Coordinated Events during Malaria Parasite Invasion of the Human Erythrocyte. Cell Host Microbe 9, 9–20 (2011). 21238943 10.1016/j.chom.2010.12.003

[b36] Fenteany G. & Zhu S. Small-molecule inhibitors of actin dynamics and cell motility. Curr Top Med Chem 3, 593–616 (2003). 12570855 10.2174/1568026033452348

[b37] Bond L. M., Tumbarello D. A., Kendrick-Jones J. & Buss F. Small-molecule inhibitors of myosin proteins. Future Med Chem 5, 41–52 (2013). 23256812 10.4155/fmc.12.185 PMC3971371

[b38] Fowler V. M., Davis J. Q. & Bennett V. Human erythrocyte myosin: identification and purification. J Cell Biol 100, 47–55 (1985). 3880759 10.1083/jcb.100.1.47 PMC2113489

[b39] Dobrowolski J. M., Carruthers V. B. & Sibley L. D. Participation of myosin in gliding motility and host cell invasion by Toxoplasma gondii. Mol Microbiol 26, 163–173 (1997). 9383198 10.1046/j.1365-2958.1997.5671913.x

[b40] Ryning F. W. & Remington J. S. Effect of cytochalasin D on Toxoplasma gondii cell entry. Infect Immun 20, 739–743 (1978). 669821 10.1128/iai.20.3.739-743.1978 PMC421921

[b41] Glushakova S. *et al.* New stages in the program of malaria parasite egress imaged in normal and sickle erythrocytes. Curr Biol 20, 1117–1121 (2010). 20537541 10.1016/j.cub.2010.04.051 PMC3541015

[b42] Atkinson M. A., Morrow J. S. & Marchesi V. T. The polymeric state of actin in the human erythrocyte cytoskeleton. J Cell Biochem 18, 493–505 (1982). 7200988 10.1002/jcb.1982.240180410

[b43] Pantaleo A., De Franceschi L., Ferru E., Vono R. & Turrini F. Current knowledge about the functional roles of phosphorylative changes of membrane proteins in normal and diseased red cells. J Proteomics 73, 445–455 (2010). 19758581 10.1016/j.jprot.2009.08.011

[b44] Thompson A. *et al.* Tandem mass tags: a novel quantification strategy for comparative analysis of complex protein mixtures by MS/MS. Anal Chem 75, 1895–1904 (2003). 12713048 10.1021/ac0262560

[b45] Boyle M. J., Richards J. S., Gilson P. R., Chai W. & Beeson J. G. Interactions with heparin-like molecules during erythrocyte invasion by Plasmodium falciparum merozoites. Blood 115, 4559–4568 (2010). 20220119 10.1182/blood-2009-09-243725

[b46] Weiss G. E. *et al.* Revealing the sequence and resulting cellular morphology of receptor-ligand interactions during Plasmodium falciparum invasion of erythrocytes. PLoS Pathog 11, e1004670 (2015). 25723550 10.1371/journal.ppat.1004670 PMC4344246

[b47] Solyakov L. *et al.* Global kinomic and phospho-proteomic analyses of the human malaria parasite Plasmodium falciparum. Nat Commun 2, 565 (2011). 22127061 10.1038/ncomms1558

[b48] Dluzewski A. R., Nash G. B., Wilson R. J., Reardon D. M. & Gratzer W. B. Invasion of hereditary ovalocytes by Plasmodium falciparum *in vitro* and its relation to intracellular ATP concentration. Mol Biochem Parasitol 55, 1–7 (1992). 1435863 10.1016/0166-6851(92)90121-y

[b49] Tham W.-H. *et al.* Complement receptor 1 is the host erythrocyte receptor for Plasmodium falciparum PfRh4 invasion ligand. Proc Natl Acad Sci USA 107, 17327–17332 (2010). 20855594 10.1073/pnas.1008151107 PMC2951459

[b50] Spadafora C. *et al.* Complement receptor 1 is a sialic acid-independent erythrocyte receptor of Plasmodium falciparum. PLoS Pathog 6, e1000968 (2010). 20585558 10.1371/journal.ppat.1000968 PMC2887475

[b51] Orlandi P. A., Klotz F. W. & Haynes J. D. A malaria invasion receptor, the 175-kilodalton erythrocyte binding antigen of Plasmodium falciparum recognizes the terminal Neu5Ac(alpha 2-3)Gal- sequences of glycophorin A. J Cell Biol 116, 901–909 (1992). 1310320 10.1083/jcb.116.4.901 PMC2289329

[b52] Chasis J. A., Mohandas N. & Shohet S. B. Erythrocyte membrane rigidity induced by glycophorin A-ligand interaction. Evidence for a ligand-induced association between glycophorin A and skeletal proteins. J Clin Invest 75, 1919–1926 (1985). 4008645 10.1172/JCI111907 PMC425549

[b53] Knowles D. W., Chasis J. A., Evans E. A. & Mohandas N. Cooperative action between band 3 and glycophorin A in human erythrocytes: immobilization of band 3 induced by antibodies to glycophorin A. Biophys J 66, 1726–1732 (1994). 8061221 10.1016/S0006-3495(94)80965-8 PMC1275892

[b54] Tham W. H., Healer J. & Cowman & A. F. Erythrocyte and reticulocyte binding-like proteins of Plasmodium falciparum. Trends Parasitol 28, 23–30 (2012). 22178537 10.1016/j.pt.2011.10.002

[b55] Duraisingh M. T. *et al.* Phenotypic variation of Plasmodium falciparum merozoite proteins directs receptor targeting for invasion of human erythrocytes. Embo J 22, 1047–1057 (2003). 12606570 10.1093/emboj/cdg096 PMC150330

[b56] Dastidar E. G. *et al.* Involvement of Plasmodium falciparum protein kinase CK2 in the chromatin assembly pathway. BMC Biol 10, 5 (2012). 22293287 10.1186/1741-7007-10-5 PMC3296614

[b57] Engelberg K. *et al.* Specific phosphorylation of the PfRh2b invasion ligand of Plasmodium falciparum. Biochem J 452, 457–466 (2013). 23544851 10.1042/BJ20121694 PMC3671792

[b58] Holland Z., Prudent R., Reiser J. B., Cochet C. & Doerig C. Functional analysis of protein kinase CK2 of the human malaria parasite Plasmodium falciparum. Eukaryot Cell 8, 388–397 (2009). 19114502 10.1128/EC.00334-08 PMC2653243

[b59] Kato K., Sudo A., Kobayashi K., Tohya Y. & Akashi H. Characterization of Plasmodium falciparum protein kinase 2. Mol Biochem Parasitol 162, 87–95 (2008). 18762219 10.1016/j.molbiopara.2008.07.007

[b60] Jones M. L., Cottingham C. & Rayner J. C. Effects of calcium signaling on Plasmodium falciparum erythrocyte invasion and post-translational modification of gliding-associated protein 45 (PfGAP45). Mol Biochem Parasitol 168, 55–62 (2009). 19576251 10.1016/j.molbiopara.2009.06.007 PMC2754074

[b61] Graciotti M. *et al.* Malaria protein kinase CK2 (PfCK2) shows novel mechanisms of regulation. PLoS One 9, e85391 (2014). 24658579 10.1371/journal.pone.0085391 PMC3962329

[b62] Coste B. *et al.* Piezo1 ion channel pore properties are dictated by C-terminal region. Nat Commun 6, 7223 (2015). 26008989 10.1038/ncomms8223 PMC4445471

[b63] Bagriantsev S. N., Gracheva E. O. & Gallagher P. G. Piezo proteins: regulators of mechanosensation and other cellular processes. J Biol Chem 289, 31673–31681 (2014). 25305018 10.1074/jbc.R114.612697 PMC4231648

[b64] Lambros C. & Vanderberg J. P. Synchronization of Plasmodium falciparum erythrocytic stages in culture. J Parasitol 65, 418–420 (1979). 383936

[b65] Smythe J. S. *et al.* Monoclonal antibodies recognizing epitopes on the extracellular face and intracellular N-terminus of the human erythrocyte anion transporter (band 3) and their application to the analysis of South East Asian ovalocytes. Blood 85, 2929–2936 (1995). 7742553

[b66] Morrison D. & Hoyle & D. C. in A practical approach to microarray data analysis. Vol. 2003 (eds D. P. Berrar, W. Dubitzky, & M. Granzow ) 76–90 (Kluwer, 2003).

[b67] Motulsky H. J. & Brown R. E. Detecting outliers when fitting data with nonlinear regression - a new method based on robust nonlinear regression and the false discovery rate. BMC bioinformatics 7, 123 (2006). 16526949 10.1186/1471-2105-7-123 PMC1472692

[b68] Eng J. K., McCormack A. L. & Yates J. R. An approach to correlate tandem mass spectral data of peptides with amino acid sequences in a protein database. J Am Soc Mass Spectrom 5, 976–989 (1994). 24226387 10.1016/1044-0305(94)80016-2

[b69] Yates J. R. 3rd, Eng J. K. & McCormack A. L. Mining genomes: correlating tandem mass spectra of modified and unmodified peptides to sequences in nucleotide databases. Anal Chem 67, 3202–3210 (1995). 8686885 10.1021/ac00114a016

[b70] Yates J. R. 3rd, Eng J. K., McCormack A. L. & Schieltz D. Method to correlate tandem mass spectra of modified peptides to amino acid sequences in the protein database. Anal Chem 67, 1426–1436 (1995). 7741214 10.1021/ac00104a020

