# Supplementary material for: Quantitative phospho-proteomics reveals the Plasmodium merozoite triggers pre-invasion host kinase modification of the red cell cytoskeleton
Source: Sci Rep. 2016 Feb 2;6:19766. doi: 10.1038/srep19766 (PMC4735681; doi:10.1038/srep19766)

Quantitative phospho-proteomics reveals the *Plasmodium* merozoite triggers pre-invasion host kinase modification of the red cell cytoskeleton

Elizabeth S. Zuccala[1*], Timothy J. Satchwell[2*], Fiona Angrisano[1], Yan Hong Tan[1], Marieangela C. Wilson[2], Kate J. Heesom[2], and Jake Baum[3]

SUPPLEMENTAL METHODS

Detection of Merozoite-Induced Erythrocyte Phosphorylation Using Radioactive Phosphate

For labelling of erythrocyte proteins with radioactive phosphate, 25 μL of uninfected erythrocytes were washed three times and resuspended in a total volume of 50 μL of phosphate-free RPMI Medium 1640 (GIBCO). Erythrocytes were incubated for 18 h at 37°C with stated concentrations of ^{32}P as $H_3{}^{32}PO_4$ (Perkin Elmer) and washed 3 times in incomplete culture medium to a final volume of 50 μL. Either 500 μL of filtered D10 M3' merozoites in incomplete medium, or incomplete medium alone, were added to labelled erythrocytes and invasion assays performed for either 2 min or 10 min. Invasion was stopped by snap-freezing samples on dry ice. Erythrocyte membrane associated proteins were prepared in the presence of 1X complete EDTA-free protease inhibitor cocktail (Roche) and 1X Halt phosphatase inhibitor cocktail (Thermo Scientific) and proteins separated by SDS PAGE. Autoradiography was performed to detect phosphorylated proteins and compared to unlabelled erythrocyte membrane proteins separated in the same way and stained with Coomassie Blue.

SUPPLEMENTAL TABLES

Supplementary Tables 1-3: These tables list the high-confidence peptides of human origin, excluding serum proteins, produced in the quantitative phospho-proteomics study. For each peptide the tables list the corresponding protein name and Uniprot ID, peptide sequence, other potential protein matches (other), peptide modifications (mod.), and for each experiment the

number of peptide reads obtained (No.) and fold-change values. Modifications detected were phosphorylation of serine, threonine and tyrosine residues (S, T and Y), carbamidomethylation of cysteine residues (C) and oxidation of methionine residues (M). Fold-change data presented are the raw median fold-change values obtained (A), the log base 2 transformed median fold-change values (B, produced by taking $\log_2$(A)) and the median centred log transformed median fold-change value (C, produced by calculating B-(global median of the dataset)). Table shaded in red list peptide data from invasion assays treated with heparin, where the fold-change values reflect peptide abundance in samples containing erythrocytes, heparin and merozoites versus samples containing erythrocytes plus heparin only. Table shaded in blue lists peptides obtained from invasion assays conducted in the presence of R1, where the fold-change values reflect peptide abundance in samples containing erythrocytes, R1 and merozoites versus samples containing erythrocytes plus R1 only. Finally, the table with green shading present peptide data obtained from uninhibited invasion assay samples, where fold-changes were produced by taking the ratio of peptide abundance between samples containing both erythrocytes and merozoites versus those containing erythrocytes alone.

Supplemental Table 4: High-confidence peptides of *P. falciparum* origin produced in the quantitative phospho-proteomics study of merozoite invasion. For each peptide the tables list the corresponding protein name and Uniprot ID, peptide sequence, peptide modifications, and for each experiment the number of peptide reads obtained (No.). Modifications detected were phosphorylation of serine, threonine and tyrosine residues (S, T and Y), carbamidomethylation of cysteine residues (C) and oxidation of methionine residues (M). Invasion assay conditions were either inhibition of invasion by heparin treatment (hep.), inhibition of invasion by R1 treatment (R1) or uninhibited invasion (inv.).

Supplemental Tables 5-7: Full-list of outlier peptides for either heparin, R1 or invasion quantitative-proteomic datasets. Tables include raw median fold change value (A), $\log_2$ (median fold change) value (B) and transformed $\log_2$ (median fold change) value (C) ranked within each experiment. Modifications on S, T and Y residues are phosphorylation events, modification of C residues is carbamidomethylation and modification of M residues is oxidation. Abbreviations:

Experiment (Exp.), peptide modification (Mod.), number of peptide reads collected (No.). *These peptides share 100% sequence identity with *P. falciparum* proteins. B4DNV4 peptide is found in Pf 3D7 heat shock 70 kDa protein, uniprot ID Q8IB24. I3L0W5 is found in putative Pf 3D7 14-3-3 protein, uniprot ID C0H4V6.

Supplemental Table 8: Outlier phospho-peptides unique to one or two assay conditions. Four outlier phospho-peptides were found exclusively in either R1 assays or R1 and invasion (uninhibited) assays. These peptides were also detected in other experiments and assay conditions. Abbreviations: Peptide modifications (Mod.), heparin (Hep.), uninhibited invasion (Inv.), experiment (Exp.).

Supplemental Table 9: Summary of outliers identified across all four experiments. Outlier proportions across experiments and conditions ranged from 3.5% - 9% of all erythrocyte peptides. While the proportion of phospho-peptides in each assay ranged from around 45% to around 60%, the proportion of outliers that were phosphorylated was much higher, indicating the outlier identification method is robust. Abbreviations: Experiment (Exp.). *Assays where some non-phosphorylated outlier peptides shared 100% sequence identity with *P. falciparum* proteins.

Supplemental Table 10: Proportion of unique erythrocyte peptides that are outliers and phospho-peptides in microbead assays.

Supplemental Table 11: Previous reports of shortlisted outlier phosphorylated residues.

Supplemental Table 12: Outlier peptides identified in assays where erythrocytes were incubated with microbeads.

Supplemental Table 13: Shortlisted erythrocyte outlier phospho-peptides from invasion proteomics were not phosphorylated in response to microbeads.

Supplemental Table 14: Top kinase predictions for shortlisted invasion phospho-peptides using NetPhosK.

SUPPLEMENTAL FIGURES

Supplemental Figure 1: *P. falciparum* growth in resealed erythrocytes. GFP expressing *P.falciparum* schizonts were mixed with erythrocytes resealed in the presence of dextran-Alexa594 and allowed to rupture, invade and develop over 24 hours. Live imaging of infected dextran-Alexa594 positive erythrocytes demonstrates parasites can invade and grow within resealed cells.

Supplemental Figure 2: Invasion into radio-labelled erythrocytes reveals merozoite-induced phosphorylation events. Erythrocytes labeled with 0.5 mCi/mL $H_3{}^{32}PO_4$ were subjected to merozoite invasion. **a)** Coomassie stain of unlabelled erythrocytes (RBCs) showing the separation of the major components of the membrane and cytoskeleton **b)** Autoradiograph with lanes corresponding to labelled erythrocytes alone (RBCs), labelled erythrocytes incubated with merozoites in the presence of heparin for two minutes (RBCs + MZ & Hep), labelled erythrocytes incubated with merozoites for two minutes without inhibition (RBCs + MZ 2 min) and labelled erythrocytes incubated with merozoites for ten minutes before snap-freezing (RBCs + MZ 10 min).. n=1.

Supplemental Figure 3: Estimated invasion rates achieved in samples used for quantitative phospho-proteomics. Prior to centrifugation, small aliquots invasion assay samples from experiments four and five were taken for invasion quantification by flow cytometry. Whereas both heparin and R1 effectively inhibit invasion, early ring-stage parasitemias of 2.1% and 3.4 % were detected for these two experiments respectively. While slightly low in comparison to standard invasion inhibition assays, this is not surprising given that invasion has only been allowed to proceed for ~ 2 minutes, compared to the 40 minutes allowed in normal flow cytometry-based invasion inhibition experiments.

Supplemental Figure 4: Outlier identification across all four invasion quantitative phosphor-proteomics experiments. Histograms of each invasion assay subjected to quantitative phospho-proteomics, displaying the transformed $\log_2$ (median fold change) value for each phosphorylated and non-phosphorylated erythrocyte peptide detected. Outlier minimums, produced using the ROUT method, are marked with the dashed line. Histogram bin width is 0.2.

Supplemental Figure 5: Peptide fold-change values for proteins containing outlier phospho-peptides. Median centered individual phospho-peptide fold-change values pooled across the four invasion proteomic experiments for proteins that contain a shortlisted outlier peptide. Graphs display box and whisker plots showing the median, maximum and minimum values and inter-quartile range. **a)** Glycophorin C; **b)** Eukaryotic translation initiation factor 4B; **c)** Glucose 1,6-bisphosphate synthase.

Supplemental Figure 6: Peptide fold-change values for proteins containing outlier phospho-peptides. Median centered individual phospho-peptide fold-change values pooled across the four invasion proteomic experiments for proteins that contain a shortlisted outlier peptide. Graphs display box and whisker plots showing the median, maximum and minimum values and inter-quartile range. **a)** Ankyrin; **b)** Protein 4.1

Supplemental Figure 7: Peptide fold-change values for membrane and cytoskeletal proteins containing that did not contain outlier phosphor-peptides. Median centered individual phospho-peptide fold-change values pooled across the four invasion proteomic experiments for erythrocyte membrane and cytoskeletal proteins that contain did not contain a shortlisted outlier peptide. Proteins were considered if they contained a phospho-peptide that was detected in at least two separate experiments. Graphs display box and whisker plots showing the median, maximum and minimum values and inter-quartile range. **a)** Alpha-spectrin; **b)** Band 3; **c)** Solute carrier family 2, facilitated glucose transporter member 1; **d)** Myosin 9.

Supplemental Figure 8: Peptide fold-change values for membrane and cytoskeletal proteins containing that did not contain outlier phosphor-peptides. Median centered individual phospho-peptide fold-change values pooled across the four invasion proteomic experiments for erythrocyte membrane and cytoskeletal proteins that contain did not contain a shortlisted outlier peptide. Proteins were considered if they contained a phospho-peptide that was detected in at least two separate experiments. Graphs display box and whisker plots showing the median, maximum and minimum values and inter-quartile range. **a)** Alpha-adducin; **b)** Dematin.

SUPPLEMENTAL DATA FILES

Supplemental Data File 1 (Excel Spread sheet)

Processed peptide data from four invasion assay experiments with merozoites. Key:

Experiments 1 and 2: Tag 126, erythrocyte alone control; Tag 127, erythrocyte alone plus R1 control; Tag 128, erythrocyte alone plus heparin control; Tag 129, heparin 1.5 min invasion assay; Tag 130, R1 1.5 min invasion assay; Tag 131, uninhibited 1.5 min invasion assay;

Experiments 3 and 4: Tag 126, erythrocyte alone control; Tag 127, uninhibited 1.5 min invasion assay; Tag 128, erythrocyte alone plus R1 control; Tag 129, R1 1.5 min invasion assay; Tag 130, erythrocyte alone plus heparin control; Tag 131, heparin 1.5 min invasion assay

Supplemental Data File 2 (Excel Spread sheet)

Processed peptide data from two invasion assay experiments with beads.

Processed peptide data from bead assays. Key: Tag 126, erythrocytes alone (experiment 1); Tag 127, erythrocytes, microbeads and BlockAid (experiment 1); Tag 128, erythrocytes and microbeads pre-incubated in wheat germ agglutinin (experiment 1); Tag 129, erythrocytes alone (experiment 2); Tag 130, erythrocytes, microbeads and BlockAid (experiment 2); Tag 131, erythrocytes and microbeads pre-incubated in wheat germ agglutinin (experiment 2)

Supplemental Table 1-3

					Experiment 1				Experiment 2				Experiment 3				Experiment 4			
**Uniprot #**	**Protein**	**Peptide**	**Mod.**	**Other**	**No.**	**A**	**B**	**C**	**No.**	**A**	**B**	**C**	**No.**	**A**	**B**	**C**	**No.**	**A**	**B**	**C**
P02549	Alpha Spectrin Erythrocytic 1	aLsNAANLQR	S3		1	1.3614	1.3614	1.5825												
		kEsLNEAQk	S3		3	0.8606	1.2111	1.4322	1	1.4912	0.5765	0.3052	1	2.0693	1.0491	0.1904	1	1.835	0.8758	-0.0475
		gLAEVQNR	None		1	1.2111	0.2763	0.4974	1	2.6838	1.4243	1.1529	1	3.0228	1.5959	0.7371				
		gTQLHEANQQLQFENNAEDLQR	None		1	1.2914	0.369	0.59	3	2.1061	1.0746	0.8033	1	2.9585	1.5649	0.7062	1	2.7491	1.459	0.5358
		vLQEESQNk	None						1	1.4056	0.4912	0.2199	1	1.9463	0.9607	0.102	1	2.1936	1.1333	0.2101
		vLETAEEIQER	None						1	1.896	0.9229	0.6516								
		rEEPGNITQR	None						1	1.4798	0.5654	0.2941								
		qEQIENQYR	None						1	1.4391	0.5251	0.2538								
B2RMN7	Beta Spectrin Erythrocytic	wDAPDDELDNDNSsAR	S14		1	0.7459	-0.4229	-0.2019	2	1.4478	0.5339	0.2626								
		lLTSQDVsYDEAR	S8		1	45.976	5.5228	5.7439	1	47.72	5.5765	5.3052								
		qIAERPAEETGPQEEEGETAGEAPVsHHAATER	S26		1	1.2334	0.3026	0.5237	2	1.6887	0.7559	0.4846								
		lLSGEDVGQDEGATR	None										1	2.0527	1.0375	0.1788				
		eNYHDQk	None										1	2.467	1.3027	0.444				
E7EV99	Alpha Adducin	sPGsPVGEGTGSPPk	S4		3	0.8476	-0.2386	-0.0175	5	1.5851	0.6646	0.3933	2	1.6849	0.7527	-0.106	1	1.7197	0.7822	-0.141
		sRsPGsPVGEGTGSPPk	S3, S6		3	1.0884	0.1222	0.3433					2	1.9471	0.9613	0.1026	3	2.9683	1.5696	0.6464
		sRsPGSPVGEGtGSPPk	S3						1	1.748	0.8057	0.5344								
		gsEENLDEAR /qkGsEENLDEAR	S2/S4		3	0.8791	-0.186	0.0351	5	1.1487	0.1999	-0.0714	2	1.5563	0.6381	-0.2206	2	2.4558	1.2962	0.373
		aAVVTsPPPTTAPHk	S6						3	1.5022	0.5871	0.3158	3	1.8626	0.8973	0.0386	4	2.3782	1.2499	0.3267
		gDEASEEGQNGssPkSk	S12, S13														1	2.6618	1.4124	0.4892
		gDEASEEGQNGsSPksk	S12, S16														1	2.5574	1.3547	0.4315
P16157	Ankyrin 1	gAsPNVSNVk	S3		1	0.9198	-0.1206	0.1005	2	1.4556	0.5417	0.2703	1	1.6236	0.6992	-0.1595				
		lGYIsVTDVLk	S5		1	0.9954	-0.0067	0.2144	1	0.6043	-0.7267	-0.9981								
		lStPPPLAEEEGLASR	T3		2	1.0901	0.1245	0.3456	2	1.4252	0.5112	0.2399	2	1.9067	0.9311	0.0723	3	1.9392	0.9554	0.0322
		eLQFsVEDINR	S5		1	0.7383	-0.4377	-0.2166	1	1.2861	0.363	0.0917	1	1.5764	0.6567	-0.202	1	1.6924	0.759	-0.1642
		aEDsDATGHEWk	S4		1	0.8416	-0.2488	-0.0277					1	1.792	0.8415	-0.0172				
		aEDSDAtGHEWk	T7						1	1.2069	0.2713	-4E-06	1	1.8838	0.9137	0.055	2	2.157	1.109	0.1858
		aEDsDAtGHEWk	S4, T7										1	1.8537	0.8904	0.0317	1	2.408	1.2679	0.3446
		lEGALsEEPR	S6		1	0.9513	-0.072	0.149	1	0.9216	-0.1177	-0.389								
		rQDDATGAGQDsENEVSLVSGHQR/ qDDATGAGQDsENEVSLVSGHQR	S12/S11		5	1.1832	0.2427	0.4638	4	1.7015	0.7668	0.4955	6	3.4044	1.7674	0.9087	5	3.7601	1.9108	0.9875
		iTHsPTVSQVTER	S4		4	0.8662	-0.2072	0.0139	4	1.3892	0.4743	0.2029	3	1.9632	0.9732	0.1145	2	1.892	0.9199	-0.0033
		iTHSPtVSQVTER	T6										1	1.8167	0.8613	0.0026				
		iTHSPtVSQVTERSQDR	T6										2	1.7144	0.7777	-0.081				
		iTHSPTVsQVTERSQDR	S8														1	2.3805	1.2513	0.3281
		iTHSPTVSQVTERsQDR	S14														1	2.771	1.4704	0.5472
		iTHsPtVSQVTER	S4, T6		1	1.6106	0.6876	0.9087	2	1.2896	0.367	0.0957	2	1.9503	0.9637	0.105				
		iTHsPTVsQVTER	S4, S8		2	0.9022	-0.1485	0.0726	3	1.1363	0.1844	-0.0869	1	1.8514	0.8886	0.0299				

					Experiment 1				Experiment 2				Experiment 3				Experiment 4			
Uniprot #	Protein	Peptide	Mod.	Other	No.	A	B	C	No.	A	B	C	No.	A	B	C	No.	A	B	C
P16157	Ankyrin 1 (Cont.)	iTHSPtVsQVTER	T6, S8						1	0.9193	-0.1213	-0.3926					1	2.6643	1.4138	0.4905
		iTHSPtVsQVTERSQDR	T6, S8														1	1.7669	0.8212	-0.102
		ySILSEStPGSLSGTEQAEmk	T8, M20						1	1.1401	0.1891	-0.0822								
		sEEQEQASk	None						1	1.8662	0.9001	0.6288								
		dSGEGDTTSLR	None										1	2.8599	1.516	0.6573				
G4V2I8	Band 3	rYQSSPAkPDsSFYk /yQSSPAkPDsSFYk	S11/S10		3	0.9592	-0.0601	0.161	5	1.3011	0.3798	0.1085	3	1.5642	0.6454	-0.2133	3	1.5682	0.6491	-0.2741
		yQSSPAkPDSsFYk	S11														1	1.8638	0.8982	-0.025
		yQSSPAkPDssFYk	S10, S11		4	0.8238	-0.2796	-0.0586	9	1.0208	0.0297	-0.2416					1	2.1297	1.0906	0.1674
		yQSsPAkPDsSFYk	S4, S10						1	1.4144	0.5002	0.2289								
		yQSSPAkPDSsFyk	S11, Y13						2	1.0545	0.0766	-0.1947								
		nVELQcLDADDAk	C6		1	0.8409	-0.25	-0.0289	1	1.2881	0.3653	0.094	1	1.6703	0.7401	-0.1186	1	1.6603	0.7314	-0.1918
P04921	Glycophorin C	gTEFAEsADAALQGDPALQDAGDSSR	S7		1	0.7202	-0.4735	-0.2524					1	6.6142	2.7256	1.8668	1	6.0521	2.5974	1.6742
		gTEFAESADAALQGDPALQDAGDsSRk/gTEFAESADAALQGDPALQDAGDsSR	S24		1	0.9965	-0.0051	0.216	1	1.135	0.1827	-0.0886	2	1.6733	0.7427	-0.116	2	1.8178	0.8622	-0.061
		gTEFAESADAALQGDPALQDAGDSsRk	S25		1	0.8008	-0.3205	-0.0994	3	1.1059	0.1452	-0.1261								
		gTEFAESADAALQGDPALQDAGDSSR	None						2	2.4654	1.3018	1.0305								
Q4VB87	Protein 4.1	sLDGAAAVDSADR	S1		5	1.3172	0.3975	0.6185	4	1.4117	0.4974	0.2261	6	1.9597	0.9706	0.1119	5	2.8257	1.4986	0.5754
		sLDGAAAVDsADR	S10		2	2.0774	1.0548	1.2758					1	2.3822	1.2523	0.3936				
		sLDGAAAVDSADRSPRPTsAPAITQGQVAEGGVLDASAk	S19		1	0.8155	-0.2943	-0.0732												
		sLDGAAAVDSADRSPRPTSAPAItQGQVAEGGVLDASAk	T24		1	0.88	-0.1845	0.0366												
		hHAsISELk	S4										1	2.0754	1.0534	0.1947	1	2.894	1.5331	0.6098
		tQTVTIsDNANAVk	S7														1	60.884	5.928	5.0048
Q08495	Dematin	qPLTSPGSVSPsR	S12		1	1.4671	0.5529	0.774	2	1.7056	0.7703	0.499								
		qREsVGGSPQTk	S4		2	0.8603	-0.2171	0.0039	1	1.4352	0.5212	0.2499								
		qRESVGGsPQTk	S8										1	1.5656	0.6467	-0.212	1	1.8626	0.8973	-0.0259
		eSVGGsPQTk	S6		1	0.9722	-0.0406	0.1804	1	1.384	0.4689	0.1976	1	1.7551	0.8116	-0.0472	1	1.8873	0.9163	-0.0069
		rGAEEEEEEEDDDsGEEmk /gAEEEEEEEDDDsGEEmk	S14,M18 / S13, M17		4	1.0103	0.0147	0.2358	5	1.0914	0.1262	-0.1451	4	1.5799	0.6599	-0.1988	6	2.197	1.1355	0.2123
		rGAEEEEEEEDDDsGEEMk /gAEEEEEEEDDDsGEEMk	S14/S13		1	0.9964	-0.0052	0.2159	2	1.1655	0.221	-0.0503	5	1.7053	0.77	-0.0887	6	1.6404	0.7141	-0.2091
		gNsLPcVLEQk	S3, C6		1	0.8053	-0.3125	-0.0914					1	1.6721	0.7416	-0.1171				
		hLsAEDFSR	S3		1	0.8825	-0.1803	0.0408	2	1.4037	0.4893	0.2179								
		ssSLPAYGR	S2						1	1.5256	0.6094	0.3381								

					Experiment 1				Experiment 2				Experiment 3				Experiment 4			
**Uniprot #**	**Protein**	**Peptide**	**Mod.**	**Other**	**No.**	**A**	**B**	**C**	**No.**	**A**	**B**	**C**	**No.**	**A**	**B**	**C**	**No.**	**A**	**B**	**C**
Q92508	Piezo-type mechanosensitive ion channel component 1	sGsEEAVTDPGER	S3		1	4.3667	2.1265	2.3476	1	5.7884	2.5332	2.2619								
		sGSEEAVtDPGER	T8										1	9.373	3.2285	2.3698	1	28.901	4.853	3.9298
P02724	Glycophorin A	kSPSDVkPLPSPDTDVPLSSVEIENPEtSDQ	T28		1	0.9039	-0.1458	0.0753												
		kSPSDVkPLPSPDTDVPLSSVEIENPETsDQ	S29		1	0.9406	-0.0884	0.1327												
K7ESE3	UV excision repair protein RAD23 homolog A	eDksPSEESAPTTSPESVSGSVPSSGSSGR	S4		1	0.6423	-0.6387	-0.4177	2	0.789	-0.3419	-0.6132	4	1.4793	0.5649	-0.2938				
		eDkSPsEESAPTTSPESVSGSVPSSGSSGR	S6		1	0.5447	-0.8766	-0.6555									2	2.0333	1.0238	0.1006
		eDkSPSEEsAPTTSPESVSGSVPSSGSSGR	S9		2	0.8221	-0.2826	-0.0615					1	1.5957	0.6742	-0.1845				
		eDksPSEESAPTTsPESVSGSVPSSGSSGR	S4, S14		1	0.6077	-0.7185	-0.4975												
Q9Y570	Protein phosphatase methylesterase 1	qcEGItSPEGSk	T6, C2		1	0.6541	-0.6123	-0.3913												
		qcEGITsPEGSk	S7, C2						1	1.3018	0.3805	0.1092	1	2.1023	1.072	0.2133				
P08238	Heat shock protein HSP 90-beta	iEDVGsDEEDDSGk/iEDVGsDEEDDSGkDk	S6		2	0.7858	-0.3477	-0.1267	2	0.9529	-0.0696	-0.3409	2	1.9876	0.9911	0.1323	3	2.1687	1.1168	0.1936
Q9Y2V2	Calcium-regulated heat stable protein 1	gNVVPsPLPTR	S6		3	0.9137	-0.1303	0.0908	1	1.2733	0.3486	0.0773								
		gNVVPSPLPtR	T10						1	1.5094	0.5939	0.3226								
Q5H924	HECT, UBA and WWE domain containing 1	sHHAASTTTAPTPAAR	S1		1	1.8787	0.9097	1.1308					1	2.8175	1.4944	0.6357				
		gSGTAsDDEFENLR	S6		1	0.8615	-0.2151	0.006	1	1.7253	0.7868	0.5155	1	1.6882	0.7555	-0.1032	1	1.8603	0.8955	-0.0277
		aEsPEEVAcR	S3, C9														1	2.6628	1.4129	0.4897
P07900	Heat shock protein HSP 90-alpha	eSEDkPEIEDVGsDEEEEk	S13		1	0.6636	-0.5916	-0.3706	1	0.3956	-1.3379	-1.6092	1	2.5503	1.3507	0.492	2	3.2706	1.7095	0.7863
P07741	Adenine phosphoribosyltransferase	iDYIAGLDsR	S9		1	0.5241	-0.9322	-0.7111	1	0.7401	-0.4341	-0.7054								
Q5TDH0	Protein DDI1 homolog 2	qPPGTQQSHsSPGEITSSPQGLDNPALLR	S10		1	0.7514	-0.4124	-0.1914	1	1.4974	0.5825	0.3111	1	2.235	1.1603	0.3016				
		qPPGTQQSHSsPGEITSSPQGLDNPALLR	S11						1	1.1187	0.1618	-0.1095								
P17812	CTP synthase 1	sGSSsPDSEITELk	S5		1	0.6589	-0.6019	-0.3808	2	0.7674	-0.382	-0.6533								
		sGSSSPDsEITELk	S8														1	2.0721	1.0511	0.1279
B3KVN0	Solute carrier family 2 (Facilitated glucose transporter), member 1	qGGASQSDkTPEELFHPLGADsQV	S22		1	0.9293	-0.1058	0.1153	1	1.5774	0.6575	0.3862	1	1.2698	0.3446	-0.5141	1	1.0492	0.0693	-0.8539
Q14C86	GTPase-activating protein and VPS9 domain-containing protein 1	sRsSDIVSSVR	S3		1	1.502	0.5869	0.8079												
		sSDIVSSVR	S1		1	1.029	0.0412	0.2623												
P68871	Hemoglobin subunit beta	lLGNVLVcVLAHHFGk	C8	Q6J1Z8; Q6VFQ6	2	0.3764	-1.4096	-1.1886	1	0.8091	-0.3055	-0.5768	7	1.5581	0.6398	-0.2189	12	1.5662	0.6473	-0.2759

					Experiment 1				Experiment 2				Experiment 3				Experiment 4			
Uniprot #	Protein	Peptide	Mod.	Other	No.	A	B	C	No.	A	B	C	No.	A	B	C	No.	A	B	C
P68871	Hemoglobin subunit beta (cont.)	gTFATLSELHcDk	C11	Q6J1Z7; Q4TZM4; Q670S4	2	0.2814	-1.8293	-1.6082	5	0.7284	-0.4572	-0.7285	5	1.4983	0.5833	-0.2754	5	1.6453	0.7184	-0.2049
		fFESFGDLSTPDAVmGNPk	M15	Q6J1Z7; Q4TZM4	1	0.3534	-1.5005	-1.2794	3	0.7721	-0.3731	-0.6444					1	2.7949	1.4828	0.5596
		fFESFGDLSTPDAVMGNPk	None	Q6J1Z7; Q4TZM4					2	0.8334	-0.2628	-0.5342								
		sAVTALWGk	S1	Q14477; Q6J1Z7; Q4TZM4	1	0.4286	-1.2221	-1.0011												
		sAVTALWGk	None	Q14477; Q6J1Z7; Q4TZM4	3	0.3862	-1.3724	-1.1513					2	1.5134	0.5978	-0.261	3	1.545	0.6276	-0.2956
		vNVDEVGGEALGR	None	Q4TZM4	6	0.4874	-1.0367	-0.8157	8	0.8651	-0.2091	-0.4804	8	1.7258	0.7872	-0.0715	11	1.7939	0.8431	-0.0801
		vVAGVANALAHk	None	P02042; Q6VFQ6	3	0.4284	-1.223	-1.0019	4	0.8651	-0.2091	-0.4804	4	1.7116	0.7754	-0.0833	5	1.7118	0.7755	-0.1477
		aLAHk	None	Q6J1Z8; Q6VFQ6									1	1.7919	0.8415	-0.0173	1	2.1123	1.0788	0.1556
		vLGAFSDGLAHLDNLk	None	P02042; Q14477; Q6J1Z7; Q670S4	6	0.3213	-1.6381	-1.4171	5	0.6821	-0.552	-0.8233	8	1.7794	0.8314	-0.0273	8	1.6382	0.7121	-0.2111
		kVLGAFSDGLAHLDNLk	None	P02042; Q14477; Q6J1Z7; Q670S4	1	0.3135	-1.6737	-1.4526					3	1.4961	0.5812	-0.2775	3	1.3789	0.4636	-0.4597
		eFTPPVQAAYQk	None		3	0.4357	-1.1985	-0.9775	4	0.9044	-0.145	-0.4163	3	1.4593	0.5453	-0.3135	3	1.5995	0.6776	-0.2456
		lLVVYPWTQR	None	P02042; Q14476; Q14477; Q6J1Z7; Q670S4; Q4TZM4	2	0.4303	-1.2165	-0.9954	1	0.7918	-0.3369	-0.6082	2	1.5106	0.5951	-0.2636	2	1.5424	0.6251	-0.2981

					Experiment 1				Experiment 2				Experiment 3				Experiment 4			
Uniprot #	Protein	Peptide	Mod.	Other	No.	A	B	C	No.	A	B	C	No.	A	B	C	No.	A	B	C
P68871	Hemoglobin subunit beta (cont.)	lHVDPENFR	None	P02042; Q6J1Z7; Q670S4					1	0.7925	-0.3355	-0.6068	3	1.6171	0.6934	-0.1653	6	1.7325	0.7929	-0.1303
P69905	Hemoglobin subunit alpha	vADALTNAVAHVDDmPNALSALSDLHAHk /kVADALTNAVAHVDDmPNALSALSDLHAHk	M15/M16		2	0.397	-1.3328	-1.1117	1	0.5569	-0.8445	-1.1158	3	1.769	0.8229	-0.0358	1	1.7195	0.782	-0.1412
		vADALTNAVAHVDDMPNALSALSDLHAHk	None		2	0.7709	-0.3754	-0.1543									1	1.683	0.7511	-0.1721
		vGAHAGEyGAEALER	Y8		2	1.1338	0.1812	0.4023	2	1.6751	0.7443	0.4729	2	2.1838	1.1268	0.2681	2	2.2364	1.1612	0.238
		vGAHAGEYGAEALER	None		8	0.4605	-1.1186	-0.8975	16	0.7823	-0.3542	-0.6255	7	2.1838	1.1268	0.2681	5	1.672	0.7416	-0.1816
		fLASVSTVLTSk	None		1	0.3907	-1.3557	-1.1346	2	0.5866	-0.7695	-1.0408	2	1.1395	0.1884	-0.6703	5	1.1068	0.1464	-0.7768
		tYFPHFDLSHGSAQVk	None		2	0.4485	-1.1569	-0.9358					2	1.6872	0.7546	-0.1041	3	1.6104	0.6874	-0.2358
		vDPVNFk	None		1	0.3763	-1.4099	-1.1889	1	0.6859	-0.544	-0.8153	3	1.4063	0.4919	-0.3669	4	1.4968	0.5819	-0.3413
		mFLSFPTTk	None		1	0.2829	-1.8216	-1.6006	1	0.7731	-0.3713	-0.6426	1	1.5309	0.6144	-0.2443	3	1.5193	0.6034	-0.3198
P02042	Hemoglobin subunit delta	gTFSQLSELHcDk	C11		1	0.4827	-1.0508	-0.8297	1	0.6822	-0.5518	-0.8231	2	2.0187	1.0134	0.1547	3	1.7729	0.8261	-0.0971
		eFTPQmQAAYQk	M6		3	0.4573	-1.1286	-0.9076	2	1.0474	0.0668	-0.2045	4	1.5418	0.6246	-0.2341	5	1.796	0.8448	-0.0784
		vNVDAVGGEALGR	None	Q6J1Z7; Q670S4	1	0.5961	-0.7464	-0.5254	1	0.7616	-0.393	-0.6643	1	1.9143	0.9368	0.0781	1	1.6074	0.6848	-0.2384
		tAVNALWGk	None	Q670S4	1	0.3162	-1.6613	-1.4402	1	0.7675	-0.3817	-0.653	1	1.557	0.6388	-0.2199	1	1.5005	0.5855	-0.3377
		lLGNVLVcVLAR	C8														1	1.5095	0.5941	-0.3291
Q6VFQ6	Hemoglobin beta chain	eFTPPVQAAYEk	None		2	0.4754	-1.0727	-0.8517	2	0.8976	-0.1559	-0.4272	3	1.5761	0.6564	-0.2023	4	1.5345	0.6178	-0.3054
P00915	Carbonic Anhydrase 1	sLLSNVEGDNAVPmQHNNRPTQPLk	M14		1	1.6511	0.7234	0.9445					1	2.1138	1.0799	0.2211				
		eSISVSSEQLAQFR	None		1	1.3457	0.4284	0.6494	2	0.9513	-0.0721	-0.3434	1	1.536	0.6192	-0.2395	2	1.5182	0.6023	-0.3209
		ySSLAEAASk	None		1	0.4163	-1.2642	-1.0432	1	0.6801	-0.5563	-0.8276	1	1.3849	0.4698	-0.3889	1	1.3831	0.4679	-0.4553
		eIINVGHSFHVNFEDNDNR	None		2	1.4935	0.5787	0.7998	1	1.4715	0.5573	0.286	1	2.0671	1.0476	0.1889				
		vLDALQAIk	None		1	0.3288	-1.6049	-1.3838	1	0.7243	-0.4652	-0.7366	2	1.5479	0.6304	-0.2284	1	1.4618	0.5477	-0.3755
		aDGLAVIGVLmk	M11										1	1.6266	0.7018	-0.1569				
		nGPEQWSk	None										3	2.3382	1.2254	0.3667				
		hDTSLkPISVSYNPATAk	None										1	1.8819	0.9122	0.0535	1	2.341	1.2271	0.3039
		vGEANPk	None										1	1.4674	0.5533	-0.3055	1	1.5616	0.643	-0.2802
		gGPFSDSYR	None														1	5.2747	2.3991	1.4759
A8K8G0	Hepatoma-derived growth factor	gNAEGssDEEGkLVIDEPAk	S6, S7		3	0.7042	-0.5059	-0.2848					2	1.656	0.7277	-0.131	1	1.6448	0.7179	-0.2053
Q7Z5Y0	EIF4B protein	sQSSDTEQQsPTSGGGk	S10		2	0.6397	-0.6445	-0.4234					1	5.3177	2.4108	1.5521	1	5.7658	2.5275	1.6043
		sQSSDtEQQSPTSGGGk	T6														1	1.6858	0.7535	-0.1697
Q8IV54	TSC22D4 protein	vEAEAGGSGARtPPLSR	T12		1	1.2586	0.3318	0.5528					1	2.7626	1.466	0.6073				
		gASGGAGGRsLDSR	S10		1	0.6957	-0.5235	-0.3025												
Q8IXQ3	Uncharacterized protein C9orf40	rDsGDNSAPSGQER	S3		2	0.6436	-0.6358	-0.4147					3	1.8141	0.8593	0.0005	1	1.9105	0.9339	0.0107
P35612	Beta Adducin	tESVTSGPmSPEGSPSksPSk	M9, S18		1	0.9393	-0.0904	0.1307												

					Experiment 1				Experiment 2				Experiment 3				Experiment 4			
**Uniprot #**	**Protein**	**Peptide**	**Mod.**	**Other**	**No.**	**A**	**B**	**C**	**No.**	**A**	**B**	**C**	**No.**	**A**	**B**	**C**	**No.**	**A**	**B**	**C**
Q5W0S3	RAD23 homolog B	qEkPAEkPAETPVATsPTATDSTSGDSSR	S16		1	0.6651	-0.5883	-0.3672									1	1.6471	0.72	-0.2032
		qEkPAEkPAETPVAtSPTATDSTSGDSSR	T15										1	1.5147	0.599	-0.2597				
H7C118	Protein phosphatase 1 regulatory subunit 7	rVEsEEsGDEEGk	S4, S7		1	0.5241	-0.9321	-0.711					1	2.1951	1.1343	0.2755				
Q5HYM4	Putative uncharacterized protein DKFZp686H1993	cDmSDLSLIGcLGysLLLMVtcTVYAIk	C1, M3, C11, Y14, S15, T21, C22		1	2.4691	1.304	1.525												
P35580	Myosin 10	qLHLEGASLELsDDDTESk	S12		1	3.3687	1.7522	1.9732												
H3BS66	Small integral membrane protein 1	dGVsLGAVsSTEEASR	S4, S9		1	1.5177	0.6019	0.8229												
		dGVSLGAVssTEEASR	S9, S10														1	2.3295	1.22	0.2968
D6RBW0	Centromere-associated protein E	tRIIcTItPVSFDETLtALQFAstAk	T8, T17, S23, T24		1	0.9794	-0.03	0.191												
Q8NH48	Olfactory receptor 5B3	mAPVFyTMVIPmLNPLVySLRNk	Y6, M12, Y18		1	0.8579	-0.2211	-4E-06												
Q0JRZ9	FCH domain only protein 2	nLsNEELTk	S3		1	0.8985	-0.1544	0.0666												
Q59G71	Tensin variant	vATTPGsPSLGR	S7		1	1.2431	0.314	0.535												
K7EM68	Serine/threonine-protein kinase BRSK1	yPScEDQDLPPR	Y1, C4		1	0.453	-1.1423	-0.9212												
Q4TZM4	Hemoglobin beta chain	kVLGAFSNGLAHLDNLk	None		1	0.431	-1.2144	-0.9933												
P32119	Peroxiredoxin-2	lSEDYGVLk	None		1	0.5941	-0.7511	-0.5301					1	1.5859	0.6653	-0.1934	2	1.6412	0.7147	-0.2085
		qITVNDLPVGR	None		1	1.0751	0.1045	0.3256	1	2.534	1.3414	1.0701	1	8.0496	3.0089	2.1502				
		tDEGIAYR	None		1	1.2766	0.3523	0.5733	1	1.3246	0.4056	0.1343	1	1.9634	0.9733	0.1146	1	1.7283	0.7894	-0.1338
		gLFIIDGk	None		1	0.5711	-0.8081	-0.5871	1	1.0333	0.0473	-0.224	1	1.4156	0.5014	-0.3573	1	1.5581	0.6397	-0.2835
		sVDEALR	None		1	0.7926	-0.3354	-0.1143					1	1.9708	0.9788	0.1201	1	1.9007	0.9265	0.0033
		aTAVVDGAFk	None						1	1.0563	0.0791	-0.1923	1	1.5129	0.5973	-0.2614	1	1.6632	0.734	-0.1893
		kEGGLGPLNIPLLADVTR	None										1	1.5212	0.6052	-0.2535	1	1.5608	0.6423	-0.2809
		iGkPAPDFk	None										1	1.5566	0.6384	-0.2203	1	1.3448	0.4274	-0.4958
B4DWK8	Catalase	nLSVEDAAR	None		1	0.8812	-0.1824	0.0386					1	2.2018	1.1387	0.28	1	3.1569	1.6585	0.7353
		fNTANDDNVTQVR	None		1	1.0249	0.0355	0.2566	1	1.1696	0.226	-0.0453	1	2.1615	1.112	0.2533	1	2.1199	1.084	0.1608
		rFNTANDDNVTQVR	None						1	1.7866	0.8372	0.5659								
		aFYVNVLNEEQR	None		1	1.6243	0.6998	0.9208												
		lSQEDPDYGIR	None		1	1.1251	0.1701	0.3911					1	1.9441	0.9591	0.1004	1	2.7038	1.435	0.5118

					Experiment 1				Experiment 2				Experiment 3				Experiment 4			
Uniprot #	Protein	Peptide	Mod.	Other	No.	A	B	C	No.	A	B	C	No.	A	B	C	No.	A	B	C
B4DWK8	Catalase (cont.)	tDQGIk	None										1	2.0638	1.0453	0.1866				
		vVHAk	None										1	1.4602	0.5462	-0.3126	1	1.6893	0.7564	-0.1668
P69892	Hemoglobin subunit gamma-2	vNVEDAGGETLGR	None	Q14476	1	0.5029	-0.9917	-0.7706					1	1.7369	0.7966	-0.0622				
		aTITSLWGk	None	B7UCU6									1	1.6903	0.7573	-0.1014				
		lHVDPENFk	None	B7UCU6									1	1.3298	0.4112	-0.4475				
		hLDDLk	None	B7UCU6									2	1.4151	0.5009	-0.3578				
J3KRH2	Haptoglobin	tEGDGVYTLNDk	None	P00738	1	1.2281	0.2965	0.5175												
Q96HD4	Similar to adducin 2 (Beta)	tESVTSGPmSPEGSPSksPSk	None		1	0.9393	-0.0904	0.1307												
P06703	Protein S100-A6	lQDAEIAR	None		1	0.7198	-0.4743	-0.2533					1	2.1296	1.0906	0.2319				
		eLTIGSk	None										1	1.6203	0.6963	-0.1624				
Q9NZD4	Alpha-hemoglobin-stabilizing protein	qQVTGEPQER	None		1	1.5037	0.5885	0.8095					1	2.5245	1.336	0.4773				
P35579	Myosin 9	kGAGDGsDEEVDGk	S7						1	0.7495	-0.4159	-0.6872	3	2.4145	1.2717	0.413	1	2.523	1.3352	0.412
H0YJ03	Proteasome subunit alpha type-3	eSLkEEDEsDDDNm	S9, M14						1	1.1107	0.1515	-0.1198								
A2RUB6	Coiled-coil domain-containing protein 66	syPGSQSQLFSQsTHk	S1, Y2, S13						1	38.765	5.2767	5.0054								
E7EQ12	Calpastatin	dTSsDkDLDDALDk	S4										1	1.7546	0.8111	-0.0476				
		dTSQsDkDLDDALDk	S5						1	1.0298	0.0424	-0.2289	2	1.7317	0.7922	-0.0665				
Q03001	Dystonin	stStQGLEHDLDDVNARWk	S1, T2, T4						1	24.082	4.5899	4.3186								
Q59HD5	Sulfurtransferase	aRsPSVAAmASPQLcR	S3, M9, C15						1	3.8905	1.96	1.6887								
H7C4J9	TSC22 domain family protein 1	sDPRTTDtAk	T8						1	1.4152	0.501	0.2297								
Q9HAP0	Valosin-containing protein	gGNIGDGGGAADR	None						1	1.3196	0.4001	0.1288								
P00918	Carbonic Anhydrase 2	ePISVSSEQVLk	None										1	2.2241	1.1532	0.2945	1	2.1477	1.1028	0.1796
		vVDVLDSIk	None										1	1.596	0.6745	-0.1842	1	1.6691	0.7391	-0.1842
		gGPLDGTYR	None										1	2.0512	1.0365	0.1777	1	2.7758	1.4729	0.5497
		vGSAkPGLQk	None										1	2.4283	1.2799	0.4212				
		dFPIAk	None										1	1.8127	0.8582	-0.0006	1	1.9061	0.9306	0.0074
		qSPVDIDTHTAk	None														1	1.9217	0.9424	0.0192
Q53HF2	Heat shock 70kDa protein 8 isoform 2 variant	sTAGDTHLGGEDFDNR	None										1	2.3563	1.2365	0.3778				
B4DNV4	cDNA FLJ53071, highly similar to Heat shock 70 kDa protein 1	aTAGDTHLGGEDFDNR	None	Q8IB24									1	5.3013	2.4063	1.5476				
Q5T4S7	E3 ubiquitin-protein ligase UBR4	hAsTSSPADk	S3										1	3.9437	1.9795	1.1208				
H3BUH7	Fructose-bisphosphate aldolase A	gILAADEStGSIAk	T9										2	2.3183	1.2131	0.3544				
		gILAADEsTGSIAk	S8										1	1.5804	0.6603	-0.1985				
P30043	Flavin reductase (NADPH)	tVAGQDAVIVLLGTR	None										1	1.5524	0.6345	-0.2242				
F5GWK0	Glucose 1,6-bisphosphate synthase	aVAGVmITAsHNR	M6, S10										1	3.244	1.6978	0.8391	1	5.8361	2.545	1.6218

					Experiment 1				Experiment 2				Experiment 3				Experiment 4			
**Uniprot #**	**Protein**	**Peptide**	**Mod.**	**Other**	**No.**	**A**	**B**	**C**	**No.**	**A**	**B**	**C**	**No.**	**A**	**B**	**C**	**No.**	**A**	**B**	**C**
F5GWK0	Glucose 1,6-bisphosphate synthase (cont.)	aVAGVMITAsHNR	S10										1	2.5284	1.3382	0.4795	1	2.0819	1.0579	0.1347
		aVAGVmItASHNR	M6, T8														1	1.7922	0.8417	-0.0815
A4UCT1	Glyceraldehyde-3-phosphate dehydrogenase	aGAHLQGGAk	None										1	2.0187	1.0135	0.1547	1	2.2238	1.1531	0.2298
		tVDGPSGk	None										1	2.2391	1.1629	0.3042	1	2.4194	1.2746	0.3514
		aAFNSGk	None														1	1.8836	0.9135	-0.0097
I3L1U0	Rab-interacting lysosomal protein	gkAESSEDEtSSPAPSk	T10										1	1.6718	0.7414	-0.1174				
		gkAESsEDEtSSPAPSk	S6, T10										1	1.8554	0.8917	0.033				
P00441	Superoxide dismutase [Cu-Zn]	aVcVLk	C3										1	1.9158	0.938	0.0793	1	2.1745	1.1207	0.1975
		gGNEESTk	None										1	1.8361	0.8767	0.018				
Q92539	Phosphatidate phosphatase LPIN2	sDSELEVkPAEsLLR	S12										1	1.9828	0.9875	0.1288				
		vIPsEDNLISEVEk	S4										1	1.7232	0.7851	-0.0736				
H0YBM4	Arf-GAP with SH3 domain, ANK repeat and PH domain-containing protein 1	qEEIDEsDDDLDDkPSPIk	S7										1	1.7605	0.816	-0.0428				
I3L0W5	14-3-3 protein epsilon	lAEQAER	None										1	4.3833	2.132	1.2733				
Q53T94	TATA box-binding protein-associated factor RNA polymerase I subunit B	aFDEk	None										1	1.7884	0.8387	-0.02				
Q5STZ8	ATP-binding cassette sub-family F (GCN20) member 1	kAEQGsEEEGEGEEEEEEGGESk	S6										1	2.0643	1.0456	0.1869	2	2.0831	1.0588	0.1355
J3KSH8	Hematological and neurological-expressed 1 protein	rNSsEASSGDFLDLk	S4										1	1.724	0.7858	-0.0729	1	2.1389	1.0969	0.1736
Q5T619	Zinc finger protein 648	aLGSLPSGLAHk	None										1	1.4928	0.578	-0.2807				
Q9UQ98	Multidrug resistance protein	hHNStAELQk	T5										1	2.9478	1.5596	0.7009				
Q14587	Zinc finger protein 268	lLVHQRmHTR	M7														1	1.7647	0.8194	-0.1038
B4DZX7	Thioredoxin domain containing, isoform CRA_b	kVEEEQEADEEDVsEEEAESk	S14														1	2.2204	1.1508	0.2276
Q9C0C9	Ubiquitin-conjugating enzyme E2 O	lIHGEDsDsEGEEEGR	S7, S9														2	2.7299	1.4489	0.5257
Q9BZ23	Pantothenate kinase 2, mitochondrial	rAsSASVPAVGASAEGTR	S3														1	7.7342	2.9512	2.028
Q9H8W4	Pleckstrin homology domain-containing family F member 2	sDSYSQsLk	S7														1	100	6.6439	5.7206
A5JTV0	MutL homolog 1	cAYRAsYSDGk	S6														1	46.48	5.5385	4.6153
I3L1A3	Ribosomal L1 domain-containing protein 1	kAVDALLtHck	T8 C10														1	1.737	0.7966	-0.1266
H7BZV9	Ankyrin repeat domain-containing protein 54	aSGGAQsPLR	S7														1	2.2434	1.1657	0.2425

					Experiment 1				Experiment 2				Experiment 3				Experiment 4			
**Uniprot #**	**Protein**	**Peptide**	**Mod.**	**Other**	**No.**	**A**	**B**	**C**	**No.**	**A**	**B**	**C**	**No.**	**A**	**B**	**C**	**No.**	**A**	**B**	**C**
P02549	Alpha Spectrin Erythrocytic 1	aLsNAANLQR	S3		1	1.7879	0.8382	0.2674												
		kEsLNEAQk	S3		3	1.6282	0.7032	0.1325	1	1.5288	0.6124	0.0341	1	1.9629	0.973	0.3954	1	1.8543	0.8909	0.0073
		gLAEVQNR	None		1	1.7143	0.7776	0.2068	1	2.7289	1.4483	0.87	1	2.4877	1.3148	0.7372				
		gTQLHEANQQLQFENNAEDLQR	None		1	2.3822	1.2523	0.6815	3	2.1095	1.0769	0.4986	1	2.1766	1.1221	0.5445	1	3.077	1.6215	0.7379
		vLQEESQNk	None						1	1.4629	0.5488	-0.0295	1	1.7941	0.8433	0.2657	1	2.0095	1.0068	0.1232
		vLETAEEIQER	None						1	1.9884	0.9916	0.4133								
		rEEPGNITQR	None						1	1.4505	0.5365	-0.0418								
		qEQIENQYR	None						1	1.3574	0.4408	-0.1375								
B2RMN7	Beta Spectrin Erythrocytic	wDAPDDELDNDNSsAR	S14		1	1.6135	0.6902	0.1194	2	1.7613	0.8166	0.2383								
		lLTSQDVsYDEAR	S8		1	54.185	5.7598	5.189	1	11.818	3.5629	2.9846								
		qIAERPAEETGPQEEEGETAGEAPVs HHAATER	S26		1	1.3266	0.4077	-0.1631	2	1.9381	0.9547	0.3764								
		lLSGEDVGQDEGATR	None										1	1.7658	0.8203	0.2427				
		eNYHDQk	None										1	2.0494	1.0352	0.4576				
E7EV99	Alpha Adducin	sPGsPVGEGTGSPPk	S4		3	1.4601	0.5461	-0.0247	5	1.5313	0.6148	0.0365	2	1.4148	0.5006	-0.077	1	1.7795	0.8315	-0.0521
		sRsPGsPVGEGTGSPPk	S3, S6		3	1.6472	0.72	0.1492					2	1.7889	0.8391	0.2615	3	2.8382	1.505	0.6213
		sRsPGSPVGEGtGSPPk	S3						1	1.5274	0.611	0.0327								
		gsEENLDEAR /qkGsEENLDEAR	S2/S4		3	1.3918	0.4769	-0.0939	5	1.4292	0.5152	-0.0631	2	1.3107	0.3904	-0.1872	2	2.3088	1.2071	0.3235
		aAVVTsPPPTTAPHk	S6						3	1.729	0.7899	0.2116	3	1.9154	0.9377	0.3601	4	2.6635	1.4133	0.5297
		gDEASEEGQNGssPkSk	S12, S13														1	2.4486	1.2919	0.4083
		gDEASEEGQNGsSPksk	S12, S16														1	2.6918	1.4286	0.5449
P16157	Ankyrin 1	gAsPNVSNVk	S3		1	1.4648	0.5507	-0.0201	2	1.5874	0.6667	0.0884	1	1.4304	0.5164	-0.0612				
		lGYIsVTDVLk	S5		1	1.594	0.6727	0.1019	1	1.0427	0.0603	-0.518								
		lStPPPLAEEEGLASR	T3		2	1.5742	0.6546	0.0838	2	1.5782	0.6583	0.08	2	1.5724	0.653	0.0754	3	2.1209	1.0846	0.201
		eLQFsVEDINR	S5		1	1.3035	0.3824	-0.1884	1	1.5223	0.6063	0.028	1	1.2717	0.3468	-0.2308	1	1.5344	0.6177	-0.266
		aEDsDATGHEWk	S4		1	1.8879	0.9168	0.346					1	1.4873	0.5727	-0.0049				
		aEDSDAtGHEWk	T7						1	1.6929	0.7595	0.1812	1	1.5006	0.5855	0.0079	2	2.0002	1.0002	0.1165
		aEDsDAtGHEWk	S4, T7										1	1.4063	0.4919	-0.0857	1	2.2428	1.1653	0.2817
		lEGALsEEPR	S6		1	1.6505	0.7229	0.1521	1	1.7996	0.8477	0.2694								
		rQDDATGAGQDsENEVSLVSGHQR/ qDDATGAGQDsENEVSLVSGHQR	S12/S11		5	1.6432	0.7165	0.1458	4	1.6759	0.7449	0.1666	6	2.4372	1.2852	0.7076	5	3.9609	1.9858	1.1022
		iTHsPTVSQVTER	S4		4	1.4599	0.5458	-0.025	4	1.6406	0.7142	0.1359	3	1.6571	0.7287	0.1511	2	1.8329	0.8742	-0.0095
		iTHSPtVSQVTER	T6										1	1.5123	0.5967	0.0191				
		iTHSPtVSQVTERSQDR	T6										2	1.4842	0.5697	-0.0079				
		iTHSPTVsQVTERSQDR	S8														1	2.2909	1.1959	0.3123
		iTHSPTVSQVTERsQDR	S14														1	2.1963	1.1351	0.2514
		iTHsPtVSQVTER	S4, T6		1	2.8248	1.4981	0.9274	2	1.308	0.3874	-0.1909	2	1.9319	0.95	0.3724				
		iTHsPTVsQVTER	S4, S8		2	1.4429	0.529	-0.0418	3	1.2728	0.348	-0.2303								

					Experiment 1				Experiment 2				Experiment 3				Experiment 4			
**Uniprot #**	**Protein**	**Peptide**	**Mod.**	**Other**	**No.**	**A**	**B**	**C**	**No.**	**A**	**B**	**C**	**No.**	**A**	**B**	**C**	**No.**	**A**	**B**	**C**
P16157	Ankyrin 1 (cont.)	iTHsPTVsQVTERSQDR	S4, S8										1	1.6249	0.7004	0.1228				
		iTHSPtVsQVTER	T6, S8						1	1.0942	0.1299	-0.4484					1	3.5734	1.8373	0.9537
		iTHSPtVsQVTERSQDR	T6, S8														1	1.7483	0.8059	-0.0777
		ySILSEStPGSLSGTEQAEmk	T8, M20						1	1.4354	0.5214	-0.0569								
		sEEQEQASk	None						1	2.0817	1.0578	0.4795								
		dSGEGDTTSLR	None										1	2.4808	1.3108	0.7332				
G4V2I8	Band 3	rYQSSPAkPDsSFYk /yQSSPAkPDsSFYk	S11/S10		3	1.4549	0.5409	-0.0299	5	1.4099	0.4956	-0.0827	3	1.3478	0.4306	-0.147	3	1.3954	0.4807	-0.4029
		yQSSPAkPDSsFYk	S11														1	1.3263	0.4074	-0.4762
		yQSSPAkPDssFYk	S10, S11		4	1.4331	0.5192	-0.0516	9	1.6195	0.6955	0.1172					1	1.8579	0.8937	0.01
		yQSsPAkPDsSFYk	S4, S10						1	1.4403	0.5263	-0.052								
		yQSSPAkPDSsFyk	S11, Y13						2	1.6697	0.7396	0.1613								
		nVELQcLDADDAk	C6		1	1.6534	0.7254	0.1546	1	1.3389	0.421	-0.1573	1	1.3474	0.4301	-0.1475	1	1.4339	0.5199	-0.3637
P04921	Glycophorin C	gTEFAEsADAALQGDPALQDAGDSSR	S7		1	1.7492	0.8067	0.2359					1	5.9156	2.5645	1.9869	1	6.5323	2.7076	1.824
		gTEFAESADAALQGDPALQDAGDsSRk/gTEFAESADAALQGDPALQDAGDsSR	S24		1	1.7354	0.7953	0.2245	1	1.3401	0.4224	-0.1559	2	1.2973	0.3756	-0.202	2	1.7385	0.7979	-0.0858
		gTEFAESADAALQGDPALQDAGDSsRk	S25		1	1.8745	0.9065	0.3357	3	1.5143	0.5987	0.0204								
		gTEFAESADAALQGDPALQDAGDSSR	None						2	2.265	1.1795	0.6012								
Q4VB87	Protein 4.1	sLDGAAAVDSADR	S1		5	2.8223	1.4969	0.9261	4	1.4996	0.5845	0.0062	6	1.6669	0.7371	0.1595	5	2.7027	1.4344	0.5508
		sLDGAAAVDsADR	S10		2	2.2906	1.1957	0.6249					1	2.0722	1.0512	0.4736				
		QVAEGGVLDASAk	S19		1	1.5971	0.6755	0.1047												
		QVAEGGVLDASAk	T24		1	1.4182	0.5041	-0.0667												
		hHAsISELk	S4										1	1.8125	0.858	0.2804	1	2.9425	1.557	0.6734
		tQTVTIsDNANAVk	S7														1	100	6.6439	5.7602
Q08495	Dematin	qPLTSPGSVSPsR	S12		1	1.9171	0.9389	0.3681	2	1.9096	0.9333	0.355								
		qREsVGGSPQTk	S4		2	1.2972	0.3754	-0.1954	1	1.4931	0.5783	4E-05								
		eSVGGsPQTk /qRESVGGsPQTk	S6/S8		1	1.3882	0.4732	-0.0976	1	1.4841	0.5696	-0.0087	3	1.4614	0.5474	-0.0302	2	1.8441	0.8829	-0.0007
		rGAEEEEEEEEDDDsGEEmk/gAEEEEEEEEDDDsGEEmk	S14, M18/S13, M17		4	1.7787	0.8308	0.26	5	1.1322	0.1791	-0.3992	4	1.1952	0.2572	-0.3204	6	2.1509	1.1049	0.2213
		rGAEEEEEEEEDDDsGEEMk/gAEEEEEEEEDDDsGEEMk	S14/S13		1	1.6532	0.7252	0.1545	2	1.1126	0.154	-0.4243	5	1.3769	0.4614	-0.1162	6	1.5301	0.6136	-0.27
		gNsLPcVLEQk	S3, C6		1	1.4888	0.5741	0.0034					1	1.3373	0.4194	-0.1582				
		hLsAEDFSR	S3		1	1.3625	0.4462	-0.1246	2	1.4775	0.5631	-0.0152								
		ssSLPAYGR	S2						1	1.6413	0.7148	0.1365								
Q92508	Piezo-type mechanosensitive ion channel component 1	sGsEEAVTDPGER	S3		1	6.108	2.6107	2.0399	1	5.7207	2.5162	1.9379								

					Experiment 1				Experiment 2				Experiment 3				Experiment 4			
**Uniprot #**	**Protein**	**Peptide**	**Mod.**	**Other**	**No.**	**A**	**B**	**C**	**No.**	**A**	**B**	**C**	**No.**	**A**	**B**	**C**	**No.**	**A**	**B**	**C**
Q92508	Piezo-type mechanosensitive ion channel component 1 (cont.)	sGSEEAVtDPGER	T8										1	8.3224	3.057	2.4794	1	35.705	5.158	4.2744
P02724	Glycophorin A	kSPSDVkPLPSPDTDVPLSSVEIENPEtSDQ	T28		1	1.2942	0.3721	-0.1987												
K7ESE3	UV excision repair protein RAD23 homolog A	eDksPSEESAPTTSPESVSGSVPSSGSSGR	S4		1	1.3806	0.4653	-0.1055	2	1.8845	0.9141	0.3358	4	1.3048	0.3838	-0.1938				
		eDkSPsEESAPTTSPESVSGSVPSSGSSGR	S6		1	1.2356	0.3053	-0.2655									2	1.6616	0.7325	-0.1511
		eDkSPSEEsAPTTSPESVSGSVPSSGSSGR	S9		2	1.9406	0.9565	0.3857					1	1.2316	0.3005	-0.2771				
		eDksPSEESAPTTsPESVSGSVPSSGSSGR	S4, S14		1	1.332	0.4136	-0.1572												
Q9Y570	Protein phosphatase methylesterase 1	qcEGItSPEGSk	T6, C2		1	1.6944	0.7608	0.19												
		qcEGITsPEGSk	S7, C2						1	1.8915	0.9196	0.3413	1	2.0354	1.0253	0.4477				
P08238	Heat shock protein HSP 90-beta	iEDVGsDEEDDSGk/iEDVGsDEEDDSGkDk	S6		2	1.5886	0.6678	0.097	2	1.4907	0.5759	-0.0024	2	1.3893	0.4743	-0.1033	3	2.128	1.0895	0.2059
Q9Y2V2	Calcium-regulated heat stable protein 1	gNVVPsPLPTR	S6		3	1.7305	0.7912	0.2204	1	1.7576	0.8136	0.2353								
		gNVVPSPLPtR	T10						1	1.8206	0.8644	0.2861								
Q5H924	HECT, UBA and WWE domain containing 1	sHHAASTTTAPTPAAR	S1		1	2.5695	1.3615	0.7907					1	2.7289	1.4483	0.8707				
		gSGTAsDDEFENLR	S6		1	1.3763	0.4608	-0.11	1	1.7135	0.777	0.1987	1	1.4546	0.5406	-0.037	1	1.845	0.8836	-4E-06
		aEsPEEVAcR	S3, C9														1	2.9812	1.5759	0.6922
P07900	Heat shock protein HSP 90-alpha	eSEDkPEIEDVGsDEEEEk	S13		1	1.7192	1.7192	1.1484	1	2.002	1.0014	0.4231	1	1.567	0.648	0.0704	2	2.5789	1.3667	0.4831
P07741	Adenine phosphoribosyltransferase	iDYIAGLDsR	S9		1	1.4062	0.4918	-0.079	1	1.3958	0.4811	-0.0972								
Q5TDH0	Protein DDI1 homolog 2	qPPGTQQSHsSPGEITSSPQGLDNPALLR	S10		1	1.7459	0.804	0.2332	1	1.9751	0.9819	0.4036	1	2.4207	1.2754	0.6978				
		qPPGTQQSHSsPGEITSSPQGLDNPALLR	S11						1	1.4106	0.4963	-0.082								
P17812	CTP synthase 1	sGSSsPDSEITELk	S5		1	1.7719	0.8253	0.2545	2	1.3678	0.4519	-0.1264								
		sGSSSPDsEITELk	S8														1	1.6891	0.7563	-0.1273
B3KVN0	Solute carrier family 2 (Facilitated glucose transporter), member 1	qGGASQSDkTPEELFHPLGADsQV	S22		1	1.2682	0.3428	-0.228	1	1.0133	0.0191	-0.5592	1	1.4234	0.5093	-0.0683	1	1.1187	0.1618	-0.7219
Q14C86	GTPase-activating protein and VPS9 domain-containing protein 1	sRsSDIVSSVR	S3		1	2.1593	1.1106	0.5398												
		sSDIVSSVR	S1		1	1.8408	0.8804	0.3096												
P68871	Hemoglobin subunit beta	lLGNVLVcVLAHHFGk	C8	Q6J1Z8; Q6VFQ6	2	1.4847	0.5702	-0.0006	1	1.5197	0.6038	0.0255	7	1.4265	0.5125	-0.0651	12	1.4735	0.5593	-0.3244
		gTFATLSELHcDk	C11	Q6J1Z7; Q4TZM4; Q670S4	2	1.1713	0.2282	-0.3426	5	1.4852	0.5707	-0.0076	5	1.3548	0.4381	-0.1395	5	1.6837	0.7516	-0.132
		fFESFGDLSTPDAVmGNPk	M15	Q6J1Z7; Q4TZM4	1	1.2162	0.2824	-0.2884	3	1.3728	0.4571	-0.1212					1	1.8954	0.9225	0.0388

					Experiment 1				Experiment 2				Experiment 3				Experiment 4			
Uniprot #	Protein	Peptide	Mod.	Other	No.	A	B	C	No.	A	B	C	No.	A	B	C	No.	A	B	C
P68871	Hemoglobin subunit beta (cont.)	fFESFGDLSTPDAVMGNPk	None	Q6J1Z7; Q4TZM4					2	1.4894	0.5747	-0.0036								
		sAVTALWGk	S1	Q14477; Q6J1Z7; Q4TZM4	1	1.5615	0.6429	0.0721												
		sAVTALWGk	None	Q14477; Q6J1Z7; Q4TZM4	3	1.326	0.4071	-0.1637					2	1.2827	0.3592	-0.2184	3	1.4826	0.5682	-0.3155
		vNVDEVGGEALGR	None	Q4TZM4	6	1.3208	0.4014	-0.1693	8	1.4443	0.5304	-0.0479	8	1.4144	0.5002	-0.0774	11	1.7342	0.7943	-0.0894
		vVAGVANALAHk	None	P02042; Q6VFQ6	3	1.352	0.4351	-0.1357	4	1.4192	0.505	-0.0733	4	1.2551	0.3278	-0.2498	5	1.5134	0.5978	-0.2858
		aLAHk	None	Q6J1Z8; Q6VFQ6									1	1.4692	0.555	-0.0226	2	1.8677	0.9013	0.0176
		vLGAFSDGLAHLDNLk	None	P02042; Q14477; Q6J1Z7; Q670S4	6	1.3057	0.3849	-0.1859	5	1.4292	0.5152	-0.0631	8	1.4564	0.5424	-0.0352	8	1.552	0.6341	-0.2495
		kVLGAFSDGLAHLDNLk	None	P02042; Q14477; Q6J1Z7; Q670S4	1	1.2283	0.2967	-0.2741					3	1.4961	0.5812	0.0036	3	1.3468	0.4295	-0.4541
		eFTPPVQAAYQk	None		3	1.3007	0.3792	-0.1915	4	1.4361	0.5221	-0.0562	3	1.3156	0.3957	-0.1819	3	1.6624	0.7333	-0.1504
		lLVVYPWTQR	None	P02042; Q14476; Q14477; Q6J1Z7; Q670S4; Q4TZM4	2	1.348	0.4308	-0.14	1	1.2987	0.377	-0.2013	2	1.3893	0.4744	-0.1032	2	1.493	0.5782	-0.3054
		lHVDPENFR	None	P02042; Q6J1Z7; Q670S4					1	1.333	0.4147	-0.1636	3	1.2268	0.295	-0.2826	6	1.537	0.6201	-0.2636
P69905	Hemoglobin subunit alpha	vADALTNAVAHVDDmPNALSALSDLHAHk/kVADALTNAVAHVDDmPNALSALSDLHAHk	M15/M16		2	1.0962	0.1325	-0.4383	1	1.2723	0.3475	-0.2308	3	1.2954	0.3733	-0.2043	1	1.3909	0.476	-0.4076
		vADALTNAVAHVDDMPNALSALSDLHAHk	None		2	1.2416	0.3123	-0.2585									1	1.7748	0.8277	-0.0559
		vGAHAGEyGAEALER	Y8		2	1.85	0.8876	0.3168	2	1.7788	0.8309	0.2526	2	1.7237	0.7855	0.2079	2	2.2264	1.1547	0.2711
		vGAHAGEYGAEALER	None		9	1.4562	0.5423	-0.0285	16	1.1665	0.2222	-0.3561	7	1.5366	0.6198	0.0422	5	1.6495	0.722	-0.1616
		fLASVSTVLTSk	None		1	1.2679	0.3424	-0.2283	2	1.1611	0.2155	-0.3628	2	0.8327	-0.2641	-0.8417	5	1.1068	0.1464	-0.7373

					Experiment 1				Experiment 2				Experiment 3				Experiment 4			
**Uniprot #**	**Protein**	**Peptide**	**Mod.**	**Other**	**No.**	**A**	**B**	**C**	**No.**	**A**	**B**	**C**	**No.**	**A**	**B**	**C**	**No.**	**A**	**B**	**C**
P69905	Hemoglobin subunit alpha (cont.)	tYFPHFDLSHGSAQVk	None		2	1.2767	0.3524	-0.2184					2	1.4124	0.4981	-0.0795	3	1.6174	0.6937	-0.1899
		vDPVNFk	None		1	1.2314	0.3003	-0.2705	1	1.1489	0.2003	-0.378	3	1.164	0.2191	-0.3585	4	1.5214	0.6054	-0.2782
		mFLSFPTTk	None		1	1.2663	0.3406	-0.2302	1	1.4049	0.4905	-0.0878	1	1.2345	0.304	-0.2736	3	1.4535	0.5395	-0.3441
P02042	Hemoglobin subunit delta	gTFSQLSELHcDk	C11		1	1.2191	0.2858	-0.285	1	1.407	0.4927	-0.0856	2	1.9059	0.9305	0.3529	3	1.6314	0.7061	-0.1775
		eFTPQmQAAYQk	M6		3	1.3063	0.3855	-0.1853	2	1.4047	0.4903	-0.088	4	1.3037	0.3826	-0.195	5	1.5865	0.6658	-0.2178
		vNVDAVGGEALGR	None	Q6J1Z7; Q670S4	1	1.5105	0.5951	0.0243	1	1.4266	0.5126	-0.0657	1	1.4368	0.5229	-0.0547	1	1.5062	0.5909	-0.2927
		tAVNALWGk	None	Q670S4	1	1.3057	0.3849	-0.1859	1	1.3817	0.4664	-0.1119	1	1.2686	0.3433	-0.2343	1	1.2974	0.3756	-0.5081
		lLGNVLVcVLAR	C8										1	1.3002	0.3787	-0.1989	1	1.6071	0.6844	-0.1992
Q6VFQ6	Hemoglobin beta chain	eFTPPVQAAYEk	None		2	1.0299	0.0425	-0.5283	2	1.2802	0.3564	-0.2219	3	1.4228	0.5087	-0.0689	4	1.5895	0.6685	-0.2151
P00915	Carbonic Anhydrase 1	sLLSNVEGDNAVPmQHNNRPTQPLk	M14		1	2.2812	1.1898	0.619					1	1.821	0.8648	0.2872				
		eSISVSSEQLAQFR	None		1	1.9727	0.9802	0.4094	2	1.5813	0.6611	0.0828	1	1.0966	0.1331	-0.4445	2	1.5069	0.5916	-0.292
		ySSLAEAASk	None		1	1.3214	0.4021	-0.1687	1	1.4477	0.5338	-0.0445	1	0.948	-0.0771	-0.6547	1	1.6953	0.7616	-0.1221
		eIINVGHSFHVNFEDNDNR	None		2	2.0584	1.0415	0.4707	1	1.7036	0.7686	0.1903	1	2.2196	1.1503	0.5727				
		vLDALQAIk	None		1	1.3937	0.4789	-0.0919	1	1.4549	0.5409	-0.0374	2	1.2508	0.3229	-0.2547	1	1.4691	0.5549	-0.3287
		aDGLAVIGVLmk	M11										1	1.1257	0.1709	-0.4067				
		nGPEQWSk	None										3	1.7187	0.7813	0.2037				
		hDTSLkPISVSYNPATAk	None										1	1.6997	0.7653	0.1877	1	2.7355	1.4518	0.5682
		vGEANPk	None										1	1.0893	0.1235	-0.4541	1	1.414	0.4998	-0.3839
		gGPFSDSYR	None														1	5.5778	2.4797	1.5961
A8K8G0	Hepatoma-derived growth factor	gNAEGssDEEGkLVIDEPAk	S6, S7		3	1.486	0.5714	0.0006					2	1.3581	0.4416	-0.136	1	1.5262	0.6099	-0.2737
Q7Z5Y0	EIF4B protein	sQSSDTEQQsPTSGGGk	S10		2	1.3585	0.442	-0.1287					1	7.7206	2.9487	2.3711	1	4.5902	2.1986	1.3149
		sQSSDtEQQSPTSGGGk	T6														1	1.4778	0.5635	-0.3202
Q8IV54	TSC22D4 protein	vEAEAGGSGARtPPLSR	T12		1	1.6252	0.7006	0.1299					1	2.4727	1.3061	0.7285				
		gASGGAGGRsLDSR	S10		1	1.4212	0.5071	-0.0637												
Q8IXQ3	Uncharacterized protein C9orf40	rDsGDNSAPSGQER	S3		2	1.2097	0.2746	-0.2961					3	1.5606	0.6421	0.0645	3	2.0431	1.0308	0.1471
P35612	Beta Adducin	tESVTSGPmSPEGSPSksPSk	M9, S18		1	1.7281	0.7892	0.2184												
Q5W0S3	RAD23 homolog B	qEkPAEkPAETPVATsPTATDSTSGDSSR	S16		1	1.5465	0.629	0.0583									1	1.7226	0.7846	-0.0991
		qEkPAEkPAETPVAtSPTATDSTSGDSSR	T15										1	1.3245	0.4055	-0.1721				
H7C118	Protein phosphatase 1 regulatory subunit 7	rVEsEEsGDEEGk	S4, S7		1	1.314	0.394	-0.1768					1	1.8893	0.9179	0.3403				
Q5HYM4	Putative uncharacterized protein DKFZp686H1993	cDmSDLSLIGcLGysLLLMVtcTVYAIk	C1, M3, C11, Y14, S15, T21, C22		1	2.5891	1.3725	0.8017												

					Experiment 1				Experiment 2				Experiment 3				Experiment 4			
**Uniprot #**	**Protein**	**Peptide**	**Mod.**	**Other**	**No.**	**A**	**B**	**C**	**No.**	**A**	**B**	**C**	**No.**	**A**	**B**	**C**	**No.**	**A**	**B**	**C**
P35580	Myosin 10	qLHLEGASLELsDDDTESk	S12		1	1.8399	0.8797	0.3089												
H3BS66	Small integral membrane protein 1	dGVsLGAVsSTEEASR	S4, S9		1	2.7966	1.4837	0.9129												
		dGVSLGAVssTEEASR	S9, S10														1	1.8993	0.9255	0.0419
D6RBW0	Centromere-associated protein E	tRIIcTItPVSFDETLtALQFAstAk	T8, T17, S23, T24		1	1.406	0.4916	-0.0792												
Q8NH48	Olfactory receptor 5B3	mAPVFyTMVIPmLNPLVySLRNk	Y6, M12, Y18		1	1.8039	0.8511	0.2803												
Q0JRZ9	FCH domain only protein 2	nLsNEELTk	S3		1	1.5179	0.602	0.0312												
Q59G71	Tensin variant	vATTPGsPSLGR	S7		1	1.6944	0.7608	0.19												
K7EM68	Serine/threonine-protein kinase BRSK1	yPScEDQDLPPR	Y1, C4		1	1.2227	0.29	-0.2808												
Q4TZM4	Hemoglobin beta chain	kVLGAFSNGLAHLDNLk	None		1	1.2602	0.3337	-0.2371												
P32119	Peroxiredoxin-2	lSEDYGVLk	None		1	1.3644	0.4483	-0.1225					1	1.255	0.3277	-0.2499	2	1.5152	0.5995	-0.2842
		qITVNDLPVGR	None		1	1.7027	0.7678	0.1971	1	2.1071	1.0753	0.497	1	8.1911	3.0341	2.4565				
		tDEGIAYR	None		1	1.7209	0.7832	0.2124	1	1.4919	0.5772	-0.0011	1	1.6596	0.7308	0.1532	1	1.869	0.9023	0.0186
		gLFIIDGk	None		1	1.4162	0.502	-0.0688	1	1.5222	0.6062	0.0279	1	1.1165	0.1589	-0.4187	1	1.478	0.5637	-0.32
		sVDEALR	None		1	1.3056	0.3847	-0.1861					1	1.8435	0.8825	0.3049	1	1.8742	0.9063	0.0227
		aTAVVDGAFk	None						1	1.3979	0.4832	-0.0951	1	1.3007	0.3793	-0.1983	1	1.4718	0.5575	-0.3261
		kEGGLGPLNIPLLADVTR	None										1	1.4439	0.53	-0.0476	1	1.5427	0.6254	-0.2582
		iGkPAPDFk	None										1	1.171	0.2278	-0.3498	1	1.1757	0.2335	-0.6501
B4DWK8	Catalase	nLSVEDAAR	None		1	1.4576	0.5436	-0.0272					1	1.9794	0.9851	0.4075	1	3.2071	1.6813	0.7976
		fNTANDDNVTQVR	None		1	1.2586	0.3318	-0.239	1	1.0485	0.0683	-0.51	1	1.9308	0.9492	0.3716	1	2.1751	1.1211	0.2374
		rFNTANDDNVTQVR	None						1	1.8291	0.8712	0.2929								
		aFYVNVLNEEQR	None		1	2.2163	1.1481	0.5774												
		lSQEDPDYGIR	None		1	1.7463	0.8043	0.2335					1	1.5715	0.6521	0.0745	1	2.4313	1.2817	0.3981
		tDQGIk	None										1	1.6769	0.7458	0.1682				
		vVHAk	None										1	1.1649	0.2203	-0.3573	1	1.6122	0.689	-0.1946
P69892	Hemoglobin subunit gamma-2	vNVEDAGGETLGR	None	Q14476	1	1.3973	0.4826	-0.0882					1	1.4886	0.5739	-0.0037				
		aTITSLWGk	None	B7UCU6									1	1.4143	0.5	-0.0776				
		lHVDPENFk	None	B7UCU6									1	1.065	0.0909	-0.4867				
		hLDDLk	None	B7UCU6									2	1.3632	0.447	-0.1306				
J3KRH2	Haptoglobin	tEGDGVYTLNDk	None	P00738	1	1.6577	0.7292	0.1584												
P06703	Protein S100-A6	lQDAEIAR	None		1	1.2971	0.3753	-0.1955					1	2.0636	1.0452	0.4676				
		eLTIGSk	None										1	1.5386	0.6216	0.044				
Q9NZD4	Alpha-hemoglobin-stabilizing protein	qQVTGEPQER	None		1	1.8132	0.8586	0.2878					1	2.8795	1.5258	0.9482				
P35579	Myosin 9	kGAGDGsDEEVDGk	S7						1	1.4105	0.4962	-0.0821	3	1.635	0.7093	0.1317	2	2.2578	1.1749	0.2913
H0YJ03	Proteasome subunit alpha type-3	eSLkEEDEsDDDNm	S9, M14						1	1.5959	0.6744	0.0961								

					Experiment 1				Experiment 2				Experiment 3				Experiment 4			
**Uniprot #**	**Protein**	**Peptide**	**Mod.**	**Other**	**No.**	**A**	**B**	**C**	**No.**	**A**	**B**	**C**	**No.**	**A**	**B**	**C**	**No.**	**A**	**B**	**C**
A2RUB6	Coiled-coil domain-containing protein 66	syPGSQSQLFSQsTHk	S1, Y2, S13						1	28.33	4.8242	4.2459								
E7EQ12	Calpastatin	dTSsDkDLDDALDk	S4										1	1.3546	0.4379	-0.1397				
		dTSQsDkDLDDALDk	S5						1	1.6628	0.7336	0.1553	2	1.4795	0.5651	-0.0125				
Q03001	Dystonin	stStQGLEHDLDDVNARWk	S1, T2, T4						1	18.189	4.185	3.6067								
Q59HD5	Sulfurtransferase	aRsPSVAAmASPQLcR	S3, M9, C15						1	3.3183	1.7305	1.1522								
H7C4J9	TSC22 domain family protein 1	sDPRTTDtAk	T8						1	1.7333	0.7935	0.2152								
Q9HAP0	Valosin-containing protein	gGNIGDGGGAADR	None						1	1.6902	0.7572	0.1789								
P00918	Carbonic Anhydrase 2	ePISVSSEQVLk	None										1	1.9151	0.9374	0.3598	1	1.9892	0.9922	0.1085
		vVDVLDSIk	None										1	1.3456	0.4282	-0.1494	1	1.4116	0.4973	-0.3863
		gGPLDGTYR	None										1	1.7357	0.7955	0.2179	1	2.9106	1.5413	0.6577
		vGSAkPGLQk	None										1	1.8993	0.9255	0.3479				
		dFPIAk	None										1	1.5347	0.618	0.0404	1	1.6786	0.7473	-0.1364
		qSPVDIDTHTAk	None														1	1.6846	0.7524	-0.1312
Q53HF2	Heat shock 70kDa protein 8 isoform 2 variant	sTAGDTHLGGEDFDNR	None										1	2.1799	1.1243	0.5467				
B4DNV4	cDNA FLJ53071, highly similar to Heat shock 70 kDa protein 1	aTAGDTHLGGEDFDNR	None	Q8IB24									1	5.5159	2.4636	1.886				
Q5T4S7	E3 ubiquitin-protein ligase UBR4	hAsTSSPADk	S3										1	3.7635	1.9121	1.3345				
H3BUH7	Fructose-bisphosphate aldolase A	gILAADEStGSIAk	T9										2	1.8691	0.9023	0.3247				
		gILAADEsTGSIAk	S8										1	1.6687	0.7388	0.1612				
P30043	Flavin reductase (NADPH)	tVAGQDAVIVLLGTR	None										1	1.3935	0.4787	-0.0989				
F5GWK0	Glucose 1,6-bisphosphate synthase	aVAGVmITAsHNR	M6, S10										1	2.299	1.201	0.6234	1	5.4437	2.4446	1.561
		aVAGVMITAsHNR	S10										1	1.9269	0.9463	0.3687	1	2.7892	1.4798	0.5962
		aVAGVmItASHNR	M6, T8														1	1.8074	0.8539	-0.0297
A4UCT1	Glyceraldehyde-3-phosphate dehydrogenase	aGAHLQGGAk	None										1	1.1169	0.1595	-0.4181	1	2.0485	1.0346	0.1509
		tVDGPSGk	None										1	2.1633	1.1132	0.5356	1	2.7184	1.4427	0.5591
		aAFNSGk	None														1	2.2194	1.1502	0.2666
I3L1U0	Rab-interacting lysosomal protein	gkAESSEDEtSSPAPSk	T10										1	1.4404	0.5265	-0.0511				
		gkAESsEDEtSSPAPSk	S6, T10										1	1.648	0.7208	0.1432				
P00441	Superoxide dismutase [Cu-Zn]	aVcVLk	C3										1	1.6517	0.7239	0.1463	1	2.2905	1.1957	0.312
		gGNEESTk	None										1	1.5051	0.5899	0.0123				
Q92539	Phosphatidate phosphatase LPIN2	sDSELEVkPAEsLLR	S12										1	1.4634	0.5493	-0.0283				
		vIPsEDNLISEVEk	S4										1	1.4242	0.5101	-0.0675				
H0YBM4	Arf-GAP with SH3 domain, ANK repeat and PH domain-containing	qEEIDEsDDDLDDkPSPIk	S7										1	1.4758	0.5615	-0.0161				
I3L0W5	14-3-3 protein epsilon	lAEQAER	None										1	3.5889	1.8435	1.2659				

					Experiment 1				Experiment 2				Experiment 3				Experiment 4			
**Uniprot #**	**Protein**	**Peptide**	**Mod.**	**Other**	**No.**	**A**	**B**	**C**	**No.**	**A**	**B**	**C**	**No.**	**A**	**B**	**C**	**No.**	**A**	**B**	**C**
Q53T94	TATA box-binding protein-associated factor RNA polymerase I	aFDEk	None										1	1.6536	0.7256	0.148				
Q5STZ8	ATP-binding cassette sub-family F (GCN20) member 1	kAEQGsEEEGEGEEEEEEGGESk	S6										1	1.5573	0.639	0.0614	2	1.8881	0.9169	0.0333
J3KSH8	Hematological and neurological-expressed 1 protein	rNSsEASSGDFLDLk	S4										1	1.4184	0.5043	-0.0733	1	1.8726	0.9051	0.0214
Q5T619	Zinc finger protein 648	aLGSLPSGLAHk	None										1	1.2278	0.2961	-0.2815				
Q9UQ98	Multidrug resistance protein	hHNStAELQk	T5										1	2.979	1.5748	0.9972				
Q14587	Zinc finger protein 268	lLVHQRmHTR	M7														1	1.6103	0.6873	-0.1963
B4DZX7	Thioredoxin domain containing, isoform CRA_b	kVEEEQEADEEDVsEEEAESk	S14														1	2.2922	1.1968	0.3131
Q9C0C9	Ubiquitin-conjugating enzyme E2 O	lIHGEDsDsEGEEEGR	S7, S9														2	2.6147	1.3867	0.503
Q9BZ23	Pantothenate kinase 2, mitochondrial	rAsSASVPAVGASAEGTR	S3														1	9.6911	3.2767	2.393
Q9H8W4	Pleckstrin homology domain-containing family F member 2	sDSYSQsLk	S7														1	100	6.6439	5.7602
A5JTV0	MutL homolog 1	cAYRAsYSDGk	S6														1	100	6.6439	5.7602
I3L1A3	Ribosomal L1 domain-containing protein 1	kAVDALLtHck	T8, C10														1	1.6698	0.7397	-0.1439
H7BZV9	Ankyrin repeat domain-containing protein 54	aSGGAQsPLR	S7														1	2.0674	1.0478	0.1642

					Experiment 1				Experiment 2				Experiment 3				Experiment 4			
Uniprot #	Protein	Peptide	Mod.	Other	No.	A	B	C	No.	A	B	C	No.	A	B	C	No.	A	B	C
P02549	Alpha Spectrin Erythrocytic 1	aLsNAANLQR	S3		1	1.5797	0.6596	0.4826												
		kEsLNEAQk	S3		3	1.2194	0.2861	0.1091	1	1.5037	0.5885	0.0314	1	1.7469	0.8048	0.3123	1	1.6592	0.7305	-0.0236
		gLAEVQNR	None		1	1.4062	0.4918	0.3148	1	2.9624	1.5668	1.0097	1	2.0493	1.0351	0.5426				
		gTQLHEANQQLQFENNAEDLQR	None		1	1.6982	0.764	0.587	3	2.3607	1.2392	0.6821	1	2.5022	1.3232	0.8307	1	2.8602	1.5161	0.762
		vLQEESQNk	None						1	1.4678	0.5536	-0.0035	1	1.6043	0.6819	0.1894	1	1.7229	0.7849	0.0308
		vLETAEEIQER	None						1	2.1743	1.1206	0.5635								
		rEEPGNITQR	None						1	1.7251	0.7867	0.2296								
		qEQIENQYR	None						1	1.7585	0.8144	0.2573								
B2RMN7	Beta Spectrin Erythrocytic	wDAPDDELDNDNSsAR	S14		1	1.3256	0.4067	0.2297	2	1.788	0.8384	0.2813								
		lLTSQDVsYDEAR	S8		1	42.335	5.4038	5.2268	1	35.325	5.1426	4.5855	1	93.555	6.5477	6.0552				
		lLTSQDVSyDEAR	Y9														1	78.298	6.2909	5.5368
		qIAERPAEETGPQEEEGETAGEAPVs HHAATER	S26		1	1.5438	0.6265	0.4495	2	1.4358	0.5219	-0.0352								
		lLSGEDVGQDEGATR	None										1	1.7815	0.8331	0.3405				
		eNYHDQk	None										1	1.6234	0.699	0.2065				
E7EV99	Alpha Adducin	sPGsPVGEGTGSPPk	S4		3	1.1185	0.1615	-0.0155	5	1.4031	0.4886	-0.0685	2	1.4292	0.5152	0.0226	1	1.5382	0.6213	-0.1328
		sRsPGsPVGEGTGSPPk	S3, S6		3	1.2727	0.3479	0.1709					2	1.6013	0.6792	0.1867	3	2.3858	1.2545	0.5004
		sRsPGSPVGEGtGSPPk	S3						1	1.7904	0.8402	0.2832								
		gsEENLDEAR /qkGsEENLDEAR	S2/S4		3	0.9474	-0.078	-0.255	5	1.2996	0.378	-0.1791	2	1.1866	0.2469	-0.2457	2	2.0195	1.014	0.2599
		aAVVTsPPPTTAPHk	S6						3	1.5503	0.6326	0.0755	3	1.6892	0.7564	0.2638	4	2.1969	1.1355	0.3814
		gDEASEEGQNGssPkSk	S12, S13														1	2.374	1.2473	0.4932
		gDEASEEGQNGsSPksk	S12, S16														1	2.1189	1.0833	0.3292
P16157	Ankyrin 1	gAsPNVSNVk	S3		1	1.1759	0.2337	0.0567	2	1.5235	0.6074	0.0503	1	1.3065	0.3857	-0.1069				
		lGYIsVTDVLk	S5		1	0.3109	-1.6854	-1.8624	1	0.9577	-0.0623	-0.6194								
		lStPPPLAEEEGLASR	T3		2	1.2836	0.3602	0.1832	2	1.5336	0.6169	0.0598	2	1.3552	0.4385	-0.054	3	1.7325	0.7928	0.0387
		eLQFsVEDINR	S5		1	1.0086	0.0123	-0.1647	1	1.3349	0.4168	-0.1403	1	1.2574	0.3305	-0.1621	1	1.4186	0.5045	-0.2496
		aEDsDATGHEWk	S4		1	1.3442	0.4268	0.2498					1	1.3971	0.4824	-0.0101				
		aEDSDAtGHEWk	T7						1	1.4847	0.5702	0.0131	1	1.3648	0.4487	-0.0438	2	1.7116	0.7753	0.0212
		aEDsDAtGHEWk	S4, T7										1	1.4122	0.498	0.0054	1	2.1145	1.0803	0.3262
		lEGALsEEPR	S6		1	1.0624	0.0873	-0.0896	1	1.5554	0.6372	0.0802								
		rQDDATGAGQDsENEVSLVSGHQR/ qDDATGAGQDsENEVSLVSGHQR	S12/S11		5	1.5655	0.6467	0.4697	4	1.6369	0.711	0.1539	6	1.92	0.9411	0.4486	5	3.2661	1.7076	0.9535
		iTHsPTVSQVTER	S4		4	1.1421	0.1917	0.0147	4	1.4936	0.5788	0.0217	3	1.4866	0.572	0.0795	2	1.6658	0.7362	-0.0179
		iTHSPtVSQVTER	T6										1	1.6347	0.709	0.2164				
		iTHSPtVSQVTERSQDR	T6										2	1.5363	0.6194	0.1269				
		iTHSPTVsQVTERSQDR	S8														1	2.124	1.0868	0.3327
		iTHSPTVSQVTERsQDR	S14														1	1.4945	0.5796	-0.1745
		iTHsPtVSQVTER	S4, T6		1	2.2967	1.1996	1.0226	2	1.257	0.33	-0.2271	2	1.4707	0.5565	0.064				

					Experiment 1				Experiment 2				Experiment 3				Experiment 4			
**Uniprot #**	**Protein**	**Peptide**	**Mod.**	**Other**	**No.**	**A**	**B**	**C**	**No.**	**A**	**B**	**C**	**No.**	**A**	**B**	**C**	**No.**	**A**	**B**	**C**
P16157	Ankyrin 1 (cont.)	iTHsPTVsQVTER	S4, S8						3	1.2644	0.3384	-0.2186								
		iTHsPTVsQVTERSQDR	S4, S8		2	1.128	0.1737	-0.0033					1	1.5981	0.6763	0.1838				
		iTHSPtVsQVTER	T6, S8						1	1.0015	0.0021	-0.5549					1	2.494	1.3184	0.5643
		iTHSPtVsQVTERSQDR	T6, S8														1	1.6918	0.7585	0.0044
		ySILSEStPGSLSGTEQAEmk	T8, M20						1	1.3539	0.4371	-0.12								
		sEEQEQASk	None						1	1.9078	0.9319	0.3748								
		dSGEGDTTSLR	None										1	2.5902	1.3731	0.8805				
G4V2I8	Band 3	rYQSSPAkPDsSFYk /yQSSPAkPDsSFYk	S11/S10		3	1.396	0.4813	0.3043	5	1.29	0.3673	-0.1898	3	1.2689	0.3436	-0.1489	3	1.3919	0.477	-0.2771
		yQSSPAkPDSsFYk	S11														1	1.2689	0.3436	-0.4105
		yQSSPAkPDssFYk	S10, S11		4	0.8703	-0.2005	-0.3775	9	1.4544	0.5404	-0.0166					1	1.5548	0.6367	-0.1174
		yQSsPAkPDsSFYk	S4, S10						1	1.3689	0.4531	-0.104								
		yQSSPAkPDSsFyk	S11, Y13						2	1.4901	0.5754	0.0183								
		nVELQcLDADDAk	C6		1	1.0968	0.1333	-0.0437	1	1.4151	0.5009	-0.0562	1	1.2525	0.3248	-0.1677	1	1.3478	0.4306	-0.3235
P04921	Glycophorin C	gTEFAEsADAALQGDPALQDAGDSSR	S7		1	0.8827	-0.18	-0.357					1	4.2082	2.0732	1.5806	1	4.5485	2.1854	1.4313
		gTEFAESADAALQGDPALQDAGDsSRk/gTEFAESADAALQGDPALQDAGDsS	S24		1	1.1543	0.207	0.03	1	1.4035	0.489	-0.0681	2	1.249	0.3208	-0.1718	2	1.6047	0.6823	-0.0718
		gTEFAESADAALQGDPALQDAGDSsRk	S25		1	0.8701	-0.2007	-0.3777	3	1.4758	0.5615	0.0045								
		gTEFAESADAALQGDPALQDAGDSSR	None						2	2.7963	1.4835	0.9265								
Q4VB87	Protein 4.1	sLDGAAAVDSADR	S1		5	2.1026	1.0722	0.8952	4	1.4533	0.5393	-0.0178	6	1.6389	0.7127	0.2201	5	2.3212	1.2149	0.4608
		sLDGAAAVDsADR	S10		2	2.0465	1.0331	0.8562					1	1.8734	0.9056	0.4131				
		sLDGAAAVDSADRSPRPTsAPAITQGQVAEGGVLDASAk	S19		1	1.1531	0.2055	0.0285												
		sLDGAAAVDSADRSPRPTSAPAItQGQVAEGGVLDASAk	T24		1	1.2553	0.328	0.151												
		hHAsISELk	S4										1	1.3976	0.4829	-0.0096	1	2.1575	1.1094	0.3553
		tQTVtISDNANAVk	T5										1	76.299	6.2536	5.761				
		tQTVTIsDNANAVk	S7														1	77.907	6.2837	5.5296
Q08495	Dematin	qPLTSPGSVSPsR	S12		1	1.538	0.621	0.444	2	1.8904	0.9187	0.3616								
		qREsVGGSPQTk	S4		2	1.1177	0.1606	-0.0164	1	1.3158	0.396	-0.1611								
		eSVGGsPQTk /qRESVGGsPQTk	S6/S8		1	1.1634	0.2184	0.0414	1	1.4748	0.5606	0.0035	3	1.4733	0.559	0.0665	2	1.6866	0.7541	-3E-06
		rGAEEEEEEEDDDsGEEmk/ gAEEEEEEEDDDsGEEmk	S14, M18/S13		4	0.5279	-0.9216	-1.0985	5	1.1176	0.1603	-0.3967	4	0.7765	-0.365	-0.8576	6	1.7546	0.8111	0.057
		rGAEEEEEEEDDDsGEEMk/ gAEEEEEEEDDDsGEEMk	S14/S13		1	0.5156	-0.9557	-1.1327	2	1.1016	0.1397	-0.4174	5	0.9049	-0.1442	-0.6367	6	1.5079	0.5925	-0.1616
		gNsLPcVLEQk	S3, C6		1	1.1714	0.2282	0.0512					1	1.4674	0.5533	0.0608				
		hLsAEDFSR	S3		1	1.07	0.0976	-0.0794	2	1.4657	0.5516	-0.0055								

					Experiment 1				Experiment 2				Experiment 3				Experiment 4			
**Uniprot #**	**Protein**	**Peptide**	**Mod.**	**Other**	**No.**	**A**	**B**	**C**	**No.**	**A**	**B**	**C**	**No.**	**A**	**B**	**C**	**No.**	**A**	**B**	**C**
Q08495	Dematin (cont.)	ssSLPAYGR	S2						1	1.4261	0.5121	-0.045								
Q92508	Piezo-type mechanosensitive ion channel component 1	sGsEEAVTDPGER	S3		1	3.1613	1.6605	1.4835	1	4.7429	2.2458	1.6887								
		sGSEEAVtDPGER	T8										1	6.1828	2.6283	2.1357	1	11.479	3.521	2.7669
P02724	Glycophorin A	kSPSDVkPLPSPDTDVPLSSVEIENPEtSDQ	T28		1	1.0187	0.0267	-0.1502												
K7ESE3	UV excision repair protein RAD23 homolog A	eDksPSEESAPTTSPESVSGSVPSSGSSGR	S4		1	1.1955	0.2576	0.0806	2	1.5733	0.6538	0.0967	4	1.2449	0.316	-0.1765				
		eDkSPsEESAPTTSPESVSGSVPSSGSSGR	S6		1	1.2061	0.2704	0.0934									2	1.4798	0.5654	-0.1887
		eDkSPSEEsAPTTSPESVSGSVPSSGSSGR	S9		2	1.4768	0.5625	0.3855					1	1.484	0.5695	0.0769				
		eDksPSEESAPTTsPESVSGSVPSSGSSGR	S4, S14		1	1.2562	0.3291	0.1521												
Q9Y570	Protein phosphatase methylesterase 1	qcEGItSPEGSk	T6, C2		1	1.1363	0.1844	0.0074												
		qcEGITsPEGSk	S7, C2						1	1.6972	0.7631	0.206	1	1.7615	0.8168	0.3243				
P08238	Heat shock protein HSP 90-beta	iEDVGsDEEDDSGk/iEDVGsDEEDDSGkDk	S6		2	0.9549	-0.0665	-0.2435	2	1.2954	0.3734	-0.1837	2	1.2593	0.3326	-0.1599	3	1.636	0.7102	-0.0439
Q9Y2V2	Calcium-regulated heat stable protein 1	gNVVPsPLPTR	S6		3	1.3277	0.4089	0.2319	1	1.5806	0.6605	0.1034								
		gNVVPSPLPtR	T10						1	1.8131	0.8585	0.3014								
Q5H924	HECT, UBA and WWE domain containing 1	sHHAASTTTAPTPAAR	S1		1	2.3112	1.2087	1.0317					1	2.2032	1.1396	0.647				
		gSGTAsDDEFENLR	S6		1	1.2155	0.2816	0.1046	1	1.6492	0.7218	0.1647	1	1.4548	0.5409	0.0483	1	1.7091	0.7733	0.0192
		aEsPEEVAcR	S3, C9														1	2.2309	1.1577	0.4036
P07900	Heat shock protein HSP 90-alpha	eSEDkPEIEDVGsDEEEEk	S13		1	0.6229	-0.683	-0.86	1	1.3735	0.4578	-0.0993	1	1.3767	0.4613	-0.0313	2	2.1332	1.093	0.3389
P07741	Adenine phosphoribosyltransferase	iDYIAGLDsR	S9		1	0.801	-0.3202	-0.4972	1	1.3286	0.4099	-0.1472								
Q5TDH0	Protein DDI1 homolog 2	qPPGTQQSHsSPGEITSSPQGLDNPALLR	S10		1	1.1012	0.1391	-0.0379	1	1.6315	0.7062	0.1491	1	1.8861	0.9154	0.4229				
		qPPGTQQSHSsPGEITSSPQGLDNPALLR	S11						1	1.5683	0.6492	0.0921								
P17812	CTP synthase 1	sGSSsPDSEITELk	S5		1	0.4222	-1.244	-1.421	2	1.2181	0.2846	-0.2725								
		sGSSSPDsEITELk	S8														1	1.7855	0.8364	0.0823
B3KVN0	Solute carrier family 2 (Facilitated glucose transporter), member 1	qGGASQSDkTPEELFHPLGADsQV	S22		1	0.7752	-0.3674	-0.5444	1	1.1366	0.1847	-0.3724	1	0.8368	-0.2571	-0.7496	1	1.2787	0.3547	-0.3994
Q14C86	GTPase-activating protein and VPS9 domain-containing protein 1	sRsSDIVSSVR	S3		1	1.842	0.8813	0.7043												
		sSDIVSSVR	S1		1	1.4937	0.5789	0.4019												
P68871	Hemoglobin subunit beta	lLGNVLVcVLAHHFGk	C8	Q6J1Z8; Q6VFQ6	2	0.9981	-0.0028	-0.1798	1	1.6974	0.7633	0.2062	7	1.2549	0.3276	-0.165	12	1.4224	0.5083	-0.2458
		gTFATLSELHcDk	C11	Q6J1Z7; Q4TZM4; Q670S4	2	0.9268	-0.1097	-0.2867	5	1.4823	0.5678	0.0107	5	1.3355	0.4174	-0.0752	5	1.6736	0.743	-0.0111

					Experiment 1				Experiment 2				Experiment 3				Experiment 4			
**Uniprot #**	**Protein**	**Peptide**	**Mod.**	**Other**	**No.**	**A**	**B**	**C**	**No.**	**A**	**B**	**C**	**No.**	**A**	**B**	**C**	**No.**	**A**	**B**	**C**
P68871	Hemoglobin subunit beta (cont.)	fFESFGDLSTPDAVmGNPk	M15	Q6J1Z7; Q4TZM4	1	0.9777	-0.0325	-0.2095	3	1.4516	0.5376	-0.0195					1	2.6835	1.4241	0.67
		fFESFGDLSTPDAVMGNPk	None	Q6J1Z7; Q4TZM4					2	1.4054	0.4909	-0.0662								
		sAVTALWGk	S1	Q14477; Q6J1Z7; Q4TZM4	1	0.7195	-0.4749	-0.6519												
		sAVTALWGk	None	Q14477; Q6J1Z7; Q4TZM4	3	0.9899	-0.0147	-0.1917					2	1.2537	0.3262	-0.1664	3	2.0824	1.0583	0.3042
		vNVDEVGGEALGR	None	Q4TZM4	6	1.1417	0.1912	0.0142	8	1.4107	0.4964	-0.0607	8	1.4014	0.4869	-0.0057	11	1.5911	0.67	-0.0841
		vVAGVANALAHk	None	P02042; Q6VFQ6	3	0.9951	-0.007	-0.184	4	1.3501	0.4331	-0.124	4	1.3016	0.3803	-0.1123	5	1.4759	0.5616	-0.1925
		aLAHk	None	Q6J1Z8; Q6VFQ6									1	1.266	0.3402	-0.1523	2	1.6777	0.7465	-0.0076
		vLGAFSDGLAHLDNLk	None	P02042; Q14477; Q6J1Z7; Q670S4	6	1.0109	0.0156	-0.1614	5	3.6434	1.8653	1.3082	8	1.3596	0.4431	-0.0494	8	1.418	0.5039	-0.2502
		kVLGAFSDGLAHLDNLk	None	P02042; Q14477; Q6J1Z7; Q670S4	1	1.0755	0.105	-0.072					3	1.5609	0.6423	0.1498	3	1.494	0.5792	-0.1749
		eFTPPVQAAYQk	None		3	1.0138	0.0198	-0.1572	4	1.4227	0.5087	-0.0484	3	1.3686	0.4527	-0.0399	3	1.4997	0.5846	-0.1695
		lLVVYPWTQR	None	P02042; Q14476; Q14477; Q6J1Z7; Q670S4; Q4TZM4	2	1.0917	0.1266	-0.0504	1	1.26	0.3334	-0.2237	2	1.3196	0.4001	-0.0925	2	1.3161	0.3963	-0.3578
		lHVDPENFR	None	P02042; Q6J1Z7; Q670S4					1	1.3651	0.449	-0.1081	3	1.209	0.2738	-0.2187	6	1.6284	0.7035	-0.0506
P69905	Hemoglobin subunit alpha	vADALTNAVAHVDDmPNALSALSDLHAHk /kVADALTNAVAHVDDmPNALSALSDLHAHk	M15/M16		2	0.9547	-0.0669	-0.2439	1	1.4517	0.5377	-0.0194	3	1.4625	0.5484	0.0559	1	1.3721	0.4563	-0.2978
		vADALTNAVAHVDDMPNALSALSDLHAHk	None		2	1.2713	0.3463	0.1693									1	1.1209	0.1646	-0.5895
		vGAHAGEyGAEALER	Y8		2	1.0581	0.0814	-0.0956	2	1.5071	0.5918	0.0347	2	1.177	0.2351	-0.2575	2	2.0357	1.0255	0.2714
		vGAHAGEYGAEALER	None		9	0.5826	-0.7793	-0.9563	16	1.2815	0.3578	-0.1993	7	1.0582	0.0816	-0.411	5	1.3315	0.4131	-0.341
		fLASVSTVLTSk	None		1	0.8315	-0.2662	-0.4432	2	1.1613	0.2158	-0.3413	2	1.1286	0.1745	-0.318	5	0.9846	-0.0223	-0.7764

					Experiment 1				Experiment 2				Experiment 3				Experiment 4			
**Uniprot #**	**Protein**	**Peptide**	**Mod.**	**Other**	**No.**	**A**	**B**	**C**	**No.**	**A**	**B**	**C**	**No.**	**A**	**B**	**C**	**No.**	**A**	**B**	**C**
P69905	Hemoglobin subunit alpha (cont.)	tYFPHFDLSHGSAQVk	None		2	0.9165	-0.1259	-0.3029					2	1.2223	0.2896	-0.2029	3	1.407	0.4927	-0.2614
		vDPVNFk	None		1	0.72	-0.4739	-0.6509	1	1.0674	0.0941	-0.463	3	1.3709	0.4551	-0.0374	4	1.3165	0.3968	-0.3573
		mFLSFPTTk	None		1	0.8681	-0.204	-0.381	1	1.389	0.474	-0.0831	1	1.2942	0.3721	-0.1205	3	1.3749	0.4593	-0.2948
P02042	Hemoglobin subunit delta	gTFSQLSELHcDk	C11		1	1.0784	0.1089	-0.0681					2	1.7117	0.7754	0.2829	3	1.5805	0.6604	-0.0937
		eFTPQmQAAYQk	M6		3	1.0824	0.1143	-0.0627	2	1.2781	0.354	-0.2031	4	1.3589	0.4425	-0.0501	5	1.6055	0.6831	-0.071
		vNVDAVGGEALGR	None	Q6J1Z7; Q670S4	1	1.1036	0.1423	-0.0347	1	1.3203	0.4008	-0.1562	1	1.2922	0.3698	-0.1227	1	1.4425	0.5286	-0.2255
		tAVNALWGk	None	Q670S4	1	0.9229	-0.1158	-0.2928	1	1.308	0.3874	-0.1697	1	1.1812	0.2403	-0.2522	1	1.3839	0.4688	-0.2853
		lLGNVLVcVLAR	C8										1	1.2706	0.3455	-0.1471	1	1.3083	0.3877	-0.3664
Q6J1Z8	Hemoglobin beta chain	eFTPPVQAAYEk	None		2	1.1028	0.1412	-0.0358	2	1.229	0.2974	-0.2597	3	1.3471	0.4298	-0.0627	4	1.409	0.4947	-0.2594
P00915	Carbonic Anhydrase 1	sLLSNVEGDNAVPmQHNNRPTQPLk	M14		1	1.7775	0.8298	0.6528					1	2.0063	1.0046	0.512				
		eSISVSSEQLAQFR	None		1	1.632	0.7066	0.5296	2	1.6768	0.7457	0.1886	1	1.401	0.4865	-0.0061	2	1.3466	0.4294	-0.3247
		ySSLAEAASk	None		1	0.8954	-0.1593	-0.3363	1	1.5323	0.6157	0.0586	1	1.289	0.3662	-0.1263	1	1.3229	0.4037	-0.3504
		eIINVGHSFHVNFEDNDNR	None		2	1.6673	0.7375	0.5605	1	1.6283	0.7033	0.1462	1	2.1484	1.1033	0.6107				
		vLDALQAIk	None		1	0.9596	-0.0595	-0.2365	1	1.3284	0.4096	-0.1474	2	1.26	0.3334	-0.1592	1	1.4523	0.5383	-0.2158
		aDGLAVIGVLmk	M11										1	1.3066	0.3858	-0.1067				
		nGPEQWSk	None										3	1.3996	0.485	-0.0075				
		hDTSLkPISVSYNPATAk	None										1	1.6353	0.7096	0.217	1	2.1422	1.0991	0.345
		vGEANPk	None										1	0.8447	-0.2434	-0.736	1	1.2121	0.2775	-0.4766
		gGPFSDSYR	None														1	4.423	2.145	1.3909
A8K8G0	Hepatoma-derived growth factor	gNAEGssDEEGkLVIDEPAk	S6, S7		3	1.1044	0.1433	-0.0337					2	1.3347	0.4166	-0.076	2	1.6263	0.7016	-0.0525
Q7Z5Y0	EIF4B protein	sQSSDTEQQsPTSGGGk	S10		2	1.2255	0.2934	0.1164					1	4.1973	2.0695	1.5769	1	4.1849	2.0652	1.3111
		sQSSDtEQQSPTSGGGk	T6														1	1.5245	0.6084	-0.1457
Q8IV54	TSC22D4 protein	vEAEAGGSGARtPPLSR	T12		1	1.4899	0.5752	0.3982					1	2.1745	1.1207	0.6281				
		gASGGAGGRsLDSR	S10		1	1.5636	0.6449	0.4679												
Q8IXQ3	Uncharacterized protein C9orf40	rDsGDNSAPSGQER	S3		2	0.9439	-0.0833	-0.2603					3	1.5311	0.6145	0.122	3	1.7562	0.8124	0.0583
P35612	Beta Adducin	tESVTSGPmSPEGSPSksPSk	M9, S18		1	1.1039	0.1426	-0.0344												
Q5W0S3	RAD23 homolog B	qEkPAEkPAETPVATsPTATDSTSGDSSR	S16		1	1.1159	0.1582	-0.0188									1	1.5513	0.6335	-0.1206
		qEkPAEkPAETPVAtSPTATDSTSGDSSR	T15										1	1.2771	0.3529	-0.1397				
H7C118	Protein phosphatase 1 regulatory subunit 7	rVEsEEsGDEEGk	S4, S7		1	1.0568	0.0797	-0.0973					1	1.6869	0.7544	0.2618				
Q5HYM4	Putative uncharacterized protein DKFZp686H1993	cDmSDLSLIGcLGysLLLMVtcTVYAIk	C1, M3, C11, [illegible]		1	2.2965	1.1994	1.0224												
P35580	Myosin 10	qLHLEGASLELsDDDTESk	S12		1	2.3203	1.2143	1.0373												
H3BS66	Small integral membrane protein 1	dGVsLGAVsSTEEASR	S4, S9		1	0.6888	-0.5379	-0.7149									1	1.9443	0.9593	0.2052
		dGVSLGAVssTEEASR	S9, S10														1	2.124	1.0868	0.3327

					Experiment 1				Experiment 2				Experiment 3				Experiment 4			
Uniprot #	Protein	Peptide	Mod.	Other	No.	A	B	C	No.	A	B	C	No.	A	B	C	No.	A	B	C
Q02224	Centromere-associated protein E	tRIIcTItPVSFDETLtALQFAstAk	T8, T17, S23, T24		1	1.0046	0.0067	-0.1703												
Q8NH48	Olfactory receptor 5B3	mAPVFyTMVIPmLNPLVySLRNk	Y6, M12, Y18		1	1.1497	0.2013	0.0243												
Q0JRZ9	FCH domain only protein 2	nLsNEELTk	S3		1	1.1552	0.2081	0.0311												
Q59G71	Tensin variant	vATTPGsPSLGR	S7		1	1.4424	0.5285	0.3515												
K7EM68	Serine/threonine-protein kinase BRSK1	yPScEDQDLPPR	Y1, C4		1	0.9933	-0.0097	-0.1867												
Q4TZM4	Hemoglobin beta chain	kVLGAFSNGLAHLDNLk	None		1	1.2587	0.3319	0.1549												
P32119	Peroxiredoxin-2	lSEDYGVLk	None		1	0.9692	-0.0451	-0.2221					1	1.2045	0.2685	-0.2241	2	1.4455	0.5316	-0.2225
		qITVNDLPVGR	None		1	1.3709	0.4552	0.2782	1	2.0293	1.021	0.4639	1	5.503	2.4602	1.9677				
		tDEGIAYR	None		1	1.3444	0.427	0.25	1	1.5074	0.5921	0.035	1	1.4813	0.5669	0.0744	1	1.6847	0.7525	-0.0016
		gLFIIDGk	None		1	1.1331	0.1802	0.0033	1	1.3486	0.4314	-0.1257	1	1.2609	0.3344	-0.1581	1	1.3454	0.428	-0.3261
		sVDEALR	None		1	0.9947	-0.0077	-0.1847					1	1.7116	0.7753	0.2828	1	1.7048	0.7696	0.0155
		aTAVVDGAFk	None						1	1.4139	0.4997	-0.0574	1	1.3764	0.4609	-0.0317	1	1.4446	0.5307	-0.2234
		kEGGLGPLNIPLLADVTR	None										1	1.2112	0.2764	-0.2162	1	1.3166	0.3968	-0.3573
		iGkPAPDFk	None										1	0.8736	-0.1949	-0.6875	1	1.2527	0.3251	-0.429
B4DWK8	Catalase	nLSVEDAAR	None		1	1.0384	0.0543	-0.1226					1	1.7518	0.8088	0.3163	1	2.3474	1.2311	0.477
		fNTANDDNVTQVR	None		1	1.1642	0.2193	0.0424	1	1.5127	0.5971	0.0401	1	1.4136	0.4994	0.0068	1	1.8085	0.8548	0.1007
		rFNTANDDNVTQVR	None						1	1.7792	0.8312	0.2741								
		aFYVNVLNEEQR	None		1	1.6459	0.7189	0.5419												
		lSQEDPDYGIR	None		1	1.2703	0.3452	0.1682					1	1.6326	0.7072	0.2146	1	2.1522	1.1058	0.3517
		tDQGIk	None										1	1.7208	0.7831	0.2906				
		vVHAk	None										1	1.3809	0.4656	-0.0269	1	1.4866	0.572	-0.1821
P69892	Hemoglobin subunit gamma-2	vNVEDAGGETLGR	None	Q14476	1	1.1244	0.1692	-0.0078					1	1.4017	0.4871	-0.0054				
		aTITSLWGk	None	B7UCU6									1	1.321	0.4016	-0.091				
		lHVDPENFk	None	B7UCU6									1	1.2053	0.2694	-0.2232				
		hLDDLk	None	B7UCU6									2	1.3548	0.4381	-0.0544				
J3KRH2	Haptoglobin	tEGDGVYTLNDk	None	P00738	1	1.2583	0.3315	0.1545												
P06703	Protein S100-A6	lQDAEIAR	None		1	1.1573	0.2107	0.0337					1	1.8079	0.8544	0.3618				
		eLTIGSk	None										1	1.4007	0.4862	-0.0064				
Q9NZD4	Alpha-hemoglobin-stabilizing protein	qQVTGEPQER	None		1	1.6667	0.737	0.56					1	3.0384	1.6033	1.1108				
P35579	Myosin 9	kGAGDGsDEEVDGk	S7						1	1.1239	0.1686	-0.3885	3	1.2509	0.323	-0.1696	2	2.096	1.0677	0.3136
H0YJ03	Proteasome subunit alpha type-3	eSLkEEDEsDDDNm	S9, M14						1	1.1969	0.2592	-0.2978								
A2RUB6	Coiled-coil domain-containing protein 66	syPGSQSQLFSQsTHk	S1, Y2, S13						1	18.58	4.2157	3.6586								
E7EQ12	Calpastatin	dTSsDkDLDDALDk	S4										1	1.5355	0.6187	0.1262				
		dTSQsDkDLDDALDk	S5						1	1.5434	0.6261	0.069	2	1.2871	0.3641	-0.1284				

					Experiment 1				Experiment 2				Experiment 3				Experiment 4			
Uniprot #	Protein	Peptide	Mod.	Other	No.	A	B	C	No.	A	B	C	No.	A	B	C	No.	A	B	C
Q03001	Dystonin	stStQGLEHDLDDVNARWk	S1, T2, T4						1	15.495	3.9537	3.3966								
Q59HD5	Sulfurtransferase	aRsPSVAAmASPQLcR	S3, M9, C15						1	2.6673	1.4154	0.8583								
H7C4J9	TSC22 domain family protein 1	sDPRTTDtAk	T8						1	1.6264	0.7017	0.1446								
Q9HAP0	Valosin-containing protein	gGNIGDGGGAADR	None						1	1.5675	0.6484	0.0913								
P00918	Carbonic Anhydrase 2	ePISVSSEQVLk	None										1	1.7189	0.7815	0.289	1	1.7469	0.8048	0.0507
		vVDVLDSIk	None										1	1.3723	0.4566	-0.036	1	1.428	0.514	-0.2401
		gGPLDGTYR	None										1	1.6944	0.7608	0.2683	1	2.388	1.2558	0.5017
		vGSAkPGLQk	None										1	1.6565	0.7281	0.2355				
		dFPIAk	None										1	1.5515	0.6337	0.1412	1	1.558	0.6397	-0.1144
		qSPVDIDTHTAk	None														1	1.6887	0.7559	0.0018
Q53HF2	Heat shock 70kDa protein 8 isoform 2 variant	sTAGDTHLGGEDFDNR	None										1	2.0458	1.0326	0.5401				
B4DNV4	cDNA FLJ53071, highly similar to Heat shock 70 kDa protein 1	aTAGDTHLGGEDFDNR	None	Q8IB24									1	2.7603	1.4648	0.9723				
Q5T4S7	E3 ubiquitin-protein ligase UBR4	hAsTSSPADk	S3										1	3.2881	1.7172	1.2247				
H3BUH7	Fructose-bisphosphate aldolase A	gILAADEStGSIAk	T9										2	1.7837	0.8349	0.3424				
		gILAADEsTGSIAk	S8										1	1.3646	0.4484	-0.0441				
P30043	Flavin reductase (NADPH)	tVAGQDAVIVLLGTR	None										1	1.2964	0.3745	-0.1181				
F5GWK0	Glucose 1,6-bisphosphate synthase	aVAGVmITAsHNR	M6, S10										1	1.9583	0.9696	0.4771	1	2.7182	1.4426	0.6885
		aVAGVMITAsHNR	S10										1	1.7324	0.7928	0.3002	1	2.1063	1.0747	0.3206
		aVAGVmItASHNR	M6, T8														1	1.498	0.583	-0.1711
A4UCT1	Glyceraldehyde-3-phosphate dehydrogenase	aGAHLQGGAk	None										1	1.2879	0.3651	-0.1275	1	2.0248	1.0178	0.2637
		tVDGPSGk	None										1	1.8068	0.8534	0.3609	1	2.0714	1.0506	0.2965
		aAFNSGk	None														1	1.9654	0.9748	0.2207
I3L1U0	Rab-interacting lysosomal protein	gkAESSEDEtSSPAPSk	T10										1	1.5673	0.6482	0.1557				
		gkAESsEDEtSSPAPSk	S6, T10										1	1.5959	0.6744	0.1818				
P00441	Superoxide dismutase [Cu-Zn]	aVcVLk	C3										1	1.7814	0.833	0.3405	1	1.951	0.9642	0.2101
		gGNEESTk	None										1	1.2849	0.3616	-0.1309				
Q92539	Phosphatidate phosphatase LPIN2	sDSELEVkPAEsLLR	S12										1	1.4472	0.5333	0.0407				
		vIPsEDNLISEVEk	S4										1	1.3923	0.4774	-0.0151				
H0YBM4	Arf-GAP with SH3 domain, ANK repeat and PH domain-containing	qEEIDEsDDDLDDkPSPIk	S7										1	1.3021	0.3809	-0.1117				
I3L0W5	14-3-3 protein epsilon	IAEQAER	None										1	3.0975	1.6311	1.1386				
Q53T94	TATA box-binding protein-associated factor RNA polymerase I	aFDEk	None										1	1.0644	0.09	-0.4025				
Q5STZ8	ATP-binding cassette sub-family F (GCN20) member 1	kAEQGsEEEGEGEEEEEEGGESk	S6										1	1.3716	0.4559	-0.0367	2	1.7739	0.8269	0.0728

					Experiment 1				Experiment 2				Experiment 3				Experiment 4			
**Uniprot #**	**Protein**	**Peptide**	**Mod.**	**Other**	**No.**	**A**	**B**	**C**	**No.**	**A**	**B**	**C**	**No.**	**A**	**B**	**C**	**No.**	**A**	**B**	**C**
J3KSH8	Hematological and neurological-expressed 1 protein	rNSsEASSGDFLDLk	S4										1	1.5054	0.5901	0.0976	1	2.1215	1.0851	0.331
Q5T619	Zinc finger protein 648	aLGSLPSGLAHk	None										1	1.2249	0.2927	-0.1999				
Q9UQ98	Multidrug resistance protein	hHNStAELQk	T5										1	2.4955	1.3193	0.8268				
Q14587	Zinc finger protein 268	lLVHQRmHTR	M7														1	1.5178	0.602	-0.1521
B4DZX7	Thioredoxin domain containing, isoform CRA_b	kVEEEQEADEEDVsEEEAESk	S14														1	2.1625	1.1127	0.3586
Q9C0C9	Ubiquitin-conjugating enzyme E2 O	lIHGEDsDsEGEEEGR	S7, S9														2	2.0488	1.0347	0.2806
Q9BZ23	Pantothenate kinase 2, mitochondrial	rAsSASVPAVGASAEGTR	S3														1	5.3903	2.4304	1.6763
Q9H8W4	Pleckstrin homology domain-containing family F member 2	sDSYSQsLk	S7														1	26.026	4.7019	3.9478
A5JTV0	MutL homolog 1	cAYRAsYSDGk	S6														1	64.21	6.0047	5.2506
I3L1A3	Ribosomal L1 domain-containing protein 1	kAVDALLtHck	T8, C10														1	1.6092	0.6863	-0.0678
H7BZV9	Ankyrin repeat domain-containing protein 54	aSGGAQsPLR	S7														1	1.7225	0.7845	0.0304

Supplemental Table 4

				Experiment 1			Experiment 2			Experiment 3			Experiment 4		
**Uniprot #**	**Protein Name**	**Peptide**	**Modification**	**Hep.**	**R1**	**Inv.**	**Hep.**	**R1**	**Inv.**	**Hep.**	**R1**	**Inv.**	**Hep.**	**R1**	**Inv.**
Q8IEC8	DnaJ/SEC63 protein, putative	rVsSDDEDDDDNEQGk	S3	1	1	1							1	1	1
		rVSsDDEDDDDNEQGk	S4	1	2	2	3	4	4	1	1	1	1	1	1
		rVssDDEDDDDNEQGk	S3, S4	4	4	4				1		1			
Q8IJI0	Pre-mRNA splicing factor, putative	nAsSDFkR	S3	1	1	1									
		nAssDFkR	S3, S4	2	2	2	2	2	2						
		yRNDYsPk	S6	2	2	2									
		eLNsDDDkk	S4		1	1		1	1						
		tVsEDRsER	S3, S7	1	1	1									
Q8IK01	Membrane skeletal protein IMC1-related	qIEDsEEIlk	S5	1	1	1	2	2	2			1	1		1
		sIQASNFEPSGsmQLk	S12, M13	1	1	1									
		rLSsEEIkPAGccSAAcT	S4, C12, C13, C17				2	2	2				1		1
		rLssEEIkPAGccSAAcT	S3, S4, C12, C13, C17				1	1	1						
		sYELLNk	S1												1
Q8IDV0	Elongation factor 1-gamma, putative	dDNNNNNNNDADNQHADLLsDDLAEk	S20	1	1	1		2	2		1		1	1	
		kDDNNNNNNNDADNQHADLLsDDLAEk	S21	3	3	3									
Q8I5S6	Eukaryotic translation initiation factor 3 subunit 10, putative	kSsIDDDLTWR	S3	2	2	2				1	1	1			1
		kNDDEEAsDGGDFTVFk	S8	1	1	1	2	2	2	1	1	1	1	1	1
		nDDEEAsDGGDFTVFk	S7				1	1	1				1	1	
		akHEtsEENTk	T5, S6				2	2	2				2	1	1
		akHEtSEENTk	T5							1	1	1	1		1
		eRsGkPENEk	S3							1		1			1
Q8IIQ7	Asparagine-rich antigen	yEcLGDADEsDk	C3, S10	1	1	1	1	1	1						1
		gGSsYDYNNR	S4	1	1	1	1	1	1						
		kDDHADsVTTk	S7				1	1	1	1	1	1	1	1	1
		nRYEcLGDADEsDk	C5, S12				2	2	2						
C6KSY4	Organic anion transporter	tNTSyEHPLNk	Y5	1	1	1	1	1	1			1	1		1
		tNTsYEHPLNk	S4												
		nkTNtsYEHPLNk	T5, S6												1
		nISsGksIDDSTDNSk	S4, S7										1		
Q8IHW1	Conserved Plasmodium protein	dAHMNsLHENYNVSTk	S6	1	1	1							1	1	1
		dAHmNsLHENYNVSTk	M4, S6	1	1	1	3	3	3				2	1	1
		dAHMNSLHENyNVSTk	Y11												1
		tAsVNGsEENVYLk	S3, S7	1	3	3									
		tASVNGsEENVYLk	S7							1		1			

Uniprot #	Protein Name	Peptide	Modification	Experiment 1			Experiment 2			Experiment 3			Experiment 4		
				Hep.	R1	Inv.	Hep.	R1	Inv.	Hep.	R1	Inv.	Hep.	R1	Inv.
Q8IIK7	Conserved Plasmodium protein	nVVsEEQSINk	S4		1	1	1	2	2						
		nVVSEEQsINk	S8												1
		nVVSEEQsINkDTk	S8							1		1			
		tTSNVsTNNTTNDHAR	S6	2	2	2	1	2	2				1	2	
		tTSNVstNNTTNDHAR	S6, T7		1	1				2	1	1	2	2	1
		tTSNVStNNTTNDHAR	T7							1			1	1	
		sSNPNNNANNNLNNk	None							1	1	1			
O77317	HAD superfamily protein, putative	nHGNDQDsDNDEDEDDDDIIDR	S8	2	2	2							1		1
		iNsLSFYEk	S3	1	1	1									
		sHskGDQDAIFDDDIQNR	S3				1	1	1	1	1	1	1	1	1
		eIDsNDDNNNHNDNNNkR	S4										1	1	1
		kPsSsLDDYTHk	S3, S5										1	1	1
Q8IAW1	RNA binding protein, putative	aksVEDNLDEEYDVGk	S3	1	1	1				2	1	1			
		kQNEDEIsNYENk	S8	1	1	1					1	2	1		1
		dRsLDHEk	S3				1	1	1						
		gkHPSyAEEDR	Y6							1	1	1		1	1
		sIEGsTkNENLk	S1, S5								1	1			
		eHEHkNtQEHIDNEQk	T7										1		1
Q8IKS5	Putative uncharacterized protein	hYNNIsNTk	S6	1	1	1	1	1	1	2	2	3	2	2	3
		rNsYINPHSHsmHEcPk	S3, S11, M12, C15										1		1
C6KT20	Putative uncharacterized protein	nIsQItIktNDDEk	S3, T6, T9	1	1	1									
		nISQItIktNDDEk	T6, T9											1	1
		nDLNIQNDENIcsRENR	C12, S13							1		1			
Q8ILW8	Deoxyhypusine synthase	iRsDDEcDADSHNEGDNIEDAk	S3, C7	2	2	2	1	1	1						
		iRSDDEcDADsHNEGDNIEDAk	C7, S11	1	1	1							1	1	1
		iRsDDEcDADsHNEGDNIEDAk	S3, C7, S11	1	1	1									
Q8IIV1	Histone H2B	kTGtGPDGk	T4	1	1	1	1	3	3						
		kTGTGPDGk	None										1	1	1
		tGTGPDGk	None										1		1
		hAVSEGTk	None									1			
C6KTB4	Acetyl-CoA synthetase	sTHsHQsDHQNDFSLQQEDNNYTk	S4, S7	1	1	1									
		sTHSHQsDHQNDFsLQQEDNNYTk	S7, S14	1	1	1									
		nDHsDDNNkNk	S4							1	1	1			

				Experiment 1			Experiment 2			Experiment 3			Experiment 4		
**Uniprot #**	**Protein Name**	**Peptide**	**Modification**	**Hep.**	**R1**	**Inv.**	**Hep.**	**R1**	**Inv.**	**Hep.**	**R1**	**Inv.**	**Hep.**	**R1**	**Inv.**
Q8ID39	Uncharacterized protein MAL13P1.336	kIDDEsFPDLLESTEk	S6	1	1	1									
		tTDGNsTEFNDk	S6	2	2	2	1	1	1	1		1	1	1	1
C0H4A7	Putative uncharacterized protein	vEGGsLEVANk	S5	1	1	1						1			1
		nDADGNNsGEENk	S8	1		1	1		1	1	1				
Q8IB63	Putative uncharacterized protein	yINDGsDDLETYkk	S6	1	1	1	1	1	1	1	1	1	1		1
		yINDGsDDLETYk	S6	1	1	1	1	2	2	1		1			1
		sNIYNNsSSHk	S7							1	1	1			
		sNIYNNSSsHk	S9										1	1	1
Q8I5T3	Non-SERCA-type Ca2+-transporting P-ATPase	dSDDIkPSVskEDLINSLk	S10	1	1	1									
		dSDDIkPsVSkEDLINSLk	S8	1	1	1									
		nDDNNsIVkVEESPk	S6	1	1	1				1		1	1	1	2
		nDDNNsIVk	S6							1	1	1			
Q8I4U3	Conserved Plasmodium protein	eAAtDDHDNDsDRk	T4, S11	1	1	1									
		eAATDDHDNDsDRk	S11				2	2	2	1	1	1	1		1
Q8IKF8	Putative uncharacterized protein	nNtEHNIHISNR	T3	1	1	1				1	1	1	1		1
		nEEsDNEQkEk	S4							1	1	1			
		nDGGsVDYNEk	S5										1		1
C0H5J5	Polyadenylate-binding protein, putative	sSsNETNkR	S3	1	1	1									
		sSsNETNk	S3	1	1	1		1	1						
		sSSNEtNk	T6		1	1	1	1	1						
Q8I5H4	Polyadenylate-binding protein, putative	kDETAAsDcADTPNILYVGPHQSR	S7, C9	1	1	1	2	2	2	2	1	1	2	1	
		kEEATASTsGAANQTTGTDADk	S9							1		1			
		kEEATASTSGAANQtTGTDADk	T15										1	1	1
		kDEtAASDcADTPNILYVGPHQSR	T4, C9										1		1
Q8IKF0	Helicase 45	cHAcVGGtVVREDIDk	C4, T8	1	1	1									
		cHAcVGGtVVR	C4, T8	1	1	1									
		cHAcVGGtVVREDIDk	C1, C4, T8				2	2	2	2		2	2	1	2
		cHAcVGGtVVR	C1, C4, T8				2	2	2	1		1	1		1
C0H4V6	14-3-3 protein, putative	dNLTLWTSDLQGDQtEEk	T15		1	1		1	1						
C6KSV0	Histone H3	sAPIsAGIk	S5	1	1	1									
		sAPISAGIk	S1	1	1	1	2	2	2						
		yRPGTVALR	None												1
		eIAQDYk	None												1
		sTDLLIR	None												1

				Experiment 1			Experiment 2			Experiment 3			Experiment 4		
**Uniprot #**	**Protein Name**	**Peptide**	**Modification**	**Hep.**	**R1**	**Inv.**	**Hep.**	**R1**	**Inv.**	**Hep.**	**R1**	**Inv.**	**Hep.**	**R1**	**Inv.**
Q8I259	Putative uncharacterized protein	gHtVDsELTTEDPVIQk	S6	2	2	2									
Q8I3K7	Membrane skeletal protein IMC1-related	eNQtIsLADEk	T4, S6	1	1	1									
		eNQTIsLADEk	S6							1		1	1	1	1
		skDsGFPSIR	S1, S4	1	1	1									
Q8IAR6	Proteasome subunit alpha type 5, putative	kENEsYEk	S5	1	1	1							1	1	1
		eNEsYEk	S4	1	1	1	1	1	1						
Q8IAL6	Mannose-6-phosphate isomerase, putative	tIsDDGVVDSYDVSk	S3	1		1	1	1	1						
		tISDDGVVDSyDVSk	Y11												1
		kVsLEYEk	S3				1	1	1						
		ecATGcsNEYTNVYDNLNk	C2, C6, S7							1	1	1			1
		gHQLNQNcDStHQk	C8, T11										1	1	1
Q8IAQ8	Vacuolar proton translocating ATPase subunit A, putative	nVEELEsLk	S7	1	1	1									
Q9TY99	Knob-associated histidine-rich protein	eAsTTEGATk	S3	1	1	1									
		hkDNEDAEsVk	S9										1		1
Q7KQL9	Fructose-bisphosphate aldolase	gGAGGENAGAsLYEk	S11	1	1	1	1	1	1	2	1	2			
		gGAGGENAGASLyEk	Y13												1
		ykGGAGGENAGAsLYEk	S13				1	1	1	1	1	1		1	1
		IENTIENR	None	1	1	1							1		1
		yASIcQQNR	C5	1	1	1							1	1	1
Q8IL59	Putative uncharacterized protein	kNSNTVDsSENVEEVVDNLTSEk	S8		2	2									
		kNSNTVDSsENVEEVVDNLTSEk	S9							1			3	2	
		kNsNTVDSSENVEEVVDNLTSEk	S3										1		
Q8I4Z3	Ubiquitin conjugating enzyme E2, putative	rDDtNEEYNQNDNINVEk	T4	2	2	2									
		rDDTNEEyNQNDNINVEk	Y8										1	1	2
Q8ILC8	DNA topoisomerase 2	kADDLNsNELDNTLsVSk	S7, S15	2	2	2									
		kADDLNSNELDNtLsVSk	T13, S15							1	1	1			
Q8IDR3	Myosin-A	rVsNVEAFDk	S3	2	2	2	2	3	3				1	1	1
Q8I6U8	Glycophorin-binding protein	sVVTEEQkVEsDSEk	S11	1	2	2		1	1	1	1	1			
C0H5B1	Asparagine-rich protein	nsNVEEHLR	S2	1	1	1	2	2	2						
		sNTNsELYNQk	S5	1	1	1							1		1
		nNsVTGNIk	S3				1	1	1						
Q8IBF6	Transcription factor with AP2 domain(S), putative	rNssDGQNk	S3, S4	1	1	1									
		nSsDGQNk	S3	1	1	1									

Uniprot #	Protein Name	Peptide	Modification	Experiment 1			Experiment 2			Experiment 3			Experiment 4		
				Hep.	R1	Inv.	Hep.	R1	Inv.	Hep.	R1	Inv.	Hep.	R1	Inv.
O77343	Transporter, putative	nLYNsGEk	S5	1	1	1	1	1	1						
Q8I305	Transporter, putative	sSINISDR	S1	1	1	1									
		sTmQGQDNIQINsER	M3, S13	1	1	1									
Q8III3	Pre-RNA processing ribonucleoprotein, putative	kHDEDQHsEHsHQDQDEQEEk	S8, S11	1	2	2									
Q8I3U4	Putative uncharacterized protein	dNNSDNyDDEDELNkk	Y7	1	2	2	2	2	2	1	1	1	1		
Q8I5I3	Phospholipid-transporting ATPase, putative	eNNsDNVk	S4	1	1	1	1	1	1						
		sNHHHNNIsDNELYDEk	S9							1	1	1			
		kNsVNTTGNR	S3										1	1	1
C0H4M6	Regulator of chromosome condensation, putative	sQDSyENHTYDk	Y5		1	1		1	1	1	1	1	1	1	2
		sNNsIDLkDEDIk	S4										1	1	1
Q8IDW4	Nuclear movement protein, putative	tIEANTSNRNSyDQVNNVNk	Y12	1	1	1									
		tIEANTSNRNsYDQVNNVNk	S11	1	1	1							1	1	
Q8I1V0	Lysine decarboxylase-like protein, putative	iNENNTsDIEsDNGEVEVHEIYNk	S7, S11	2	2	2	1	1	1						
		iNENNtSDIEsDNGEVEVHEIYNk	T6, S11				1	1	1						
Q8II64	Transporter, putative	vTYGDDAsSk	S8	1	1	1									
		kDEYAAstNNSSNLVk	S7, T8									1			
		kDEYAAsTNNSSNLVk	S7							1		1			
		kDEYAAsTNNsSNLVk	S7, S11												1
		kDEYAAStNNsSNLVk	T8, S11										1		1
		emsSNDNVNNNEk	M2, S3							1		1			
Q8I308	Putative uncharacterized protein	yYsADDLLQTTAPR	S3	2	2	2									
Q8IAY2	Putative uncharacterized protein	hNsNEYYk	S3	1	1	1									
Q8IHR6	Coatomer subunit gamma	qIsNEYEEk	S3	1	1	1	1	1	1			1			
		qISNEyEEk	Y6										1	1	1
Q8I341	Putative uncharacterized protein	sINPDNSANR	S1	1	1	1	1	1	1			1	1		1
		kIDsYDNIDNNVHk	S4							1	1	1		1	
Q8I4X0	Actin-1	tSEQsSDIEk	S5	1	1	1									
		tSEQSSDIEk	None	1	1	1									1
		tSEQSsDIEk	S6				1	1	1						
Q8IE45	Uncharacterized protein	yTSTSsLNk	S6	1	1	1	1	1	1				1		1

				Experiment 1			Experiment 2			Experiment 3			Experiment 4		
**Uniprot #**	**Protein Name**	**Peptide**	**Modification**	**Hep.**	**R1**	**Inv.**	**Hep.**	**R1**	**Inv.**	**Hep.**	**R1**	**Inv.**	**Hep.**	**R1**	**Inv.**
Q8IJB1	Conserved Plasmodium protein	aSsDPNNLAGADHSQk	S3	1	1	1				1	1	1	1	1	1
Q8IJ72	Bromodomain protein, putative	sIREDsLDDVLk	S6	1	1	1				1		1	1		1
Q8IES0	Small heat shock protein, putative	sLTDYDINR	S1	1	1	1	2	2	2						1
		sLtDYDINR	T3							1		1			
		sFtLPENVSEDEIk	T3		1	1	3	3	3						
Q8IIG7	Uncharacterized protein PF11_0207	nIsDVsDIEk	S3, S6	1	1	1									
Q8IAZ3	Eukaryotic translation initiation factor 3 subunit G	kWADIDVDDDEsINDNEIk	S12	1	1	1									
Q7K6A4	S-adenosylmethionine synthetase	vSIDEQSPDIAQcVHENR	C13	1	1	1	1	1	1				1		
C0H4A5	Putative uncharacterized protein	lQEFGIEGsk	S9	1	1	1									
		kLLsFTsNDILR	S4, S7		1	1									
Q8IJD6	Conserved Plasmodium protein	iIEHEGtDDNYILGNEEk	T7	1	1	1	1	1	1	2	2	2	1	1	
Q8IC15	Inositol phosphatase, putative	nIsIDQTNk	S3	2	2	2									
C6KSP9	RNA binding protein, putative	sYALVDNEVGNSNIsDEENIIkR	S15	1	1	1									
Q8IEF5	Exportin 1-like protein, putative	sSsNDYESSk	S3	2	2	2	1	1	1						
		sSSNDyESSk	Y6									1			
		sSSNDYEsSk	S8									1			
Q8I1Z1	Putative uncharacterized protein	vTsINNNk	S3	1	1	1	1	1	1						
		tLSSyTLk	Y5	1	1	1									
Q8IHU0	60S ribosomal protein L28, putative	lLQQHGsFEk	S7	1	1	1									
C0H4T6	Protein phosphatase, putative	rIsENEVQEVVEGEENEEEEER	S3	1	1	1									3
Q8I2Z8	6-phosphofructokinase, putative	dGGSsPTSSAk	S5	2	2	2	2	2	2						
Q8IJX3	RNA binding protein, putative	eDREENsGNFEk	S7	1	1	1	2	3	3	1		2	1		
Q8I278	Putative uncharacterized protein	nIQIYDIScsQEk	C9, S10	1	1	1									
Q8IKP8	Putative uncharacterized protein	nTNsQNIk	S4	1	1	1									

				Experiment 1			Experiment 2			Experiment 3			Experiment 4		
**Uniprot #**	**Protein Name**	**Peptide**	**Modification**	**Hep.**	**R1**	**Inv.**	**Hep.**	**R1**	**Inv.**	**Hep.**	**R1**	**Inv.**	**Hep.**	**R1**	**Inv.**
Q8ILG1	Putative uncharacterized protein	nSGsLENk	S4	1	1	1									
		rDsNEINk	S3							1		1		1	1
Q8I5I0	Integral membrane protein, putative	ykFDsEDAR	S5	1	1	1	3	3	3						
Q8IIH4	U2 snRNP auxiliary factor, small subunit, putative	nRsDNEDDDDDEER	S3	1	1	1				1	2	2	1		
Q8ID72	Uncharacterized protein	nNsLDSNk	S3	1	1	1									
Q8IIR0	U5 snRNP-associated protein, putative	sVNNTTASNISk	S1	1	1	1									
C0H5G9	Krox-like protein. putative	rNsFDLVYSDsNVNTINk	S11	1	1	1									
		qSAsIIELQNSSk	S4										1		1
P62344	Calcium-dependent protein kinase 1	kLGsGAYGEVLLcR	S4, C13	1	1	1									
		sGNNkNSEDLAINPGmyVR	M16, Y17										1	1	1
Q8IB31	Putative uncharacterized protein	kEEsDEDEDENEEk	S4	1	1	1				1	1	1	1	1	1
C6KTA4	Pyruvate kinase	vGsFQGTDIVIR	S3			1									
		vGSFQGtDIVIR	T7							1		1			1
Q8I655	membrane protein RAMP4, putative	vEAFDsNVTk	S6	1	1	1	2	3	3						
		vEAFDSNVtk	T9									1	1		1
Q8IKK7	Glyceraldehyde-3-phosphate dehydrogenase	gILGYTEDEVVSQDFVHDNR	None	1	1	1	2	2	2						
		aAEGPLk	None												1
Q8IJS1	Hypoxanthine phosphoribosyltransferase	vksYcNDQSTGTLEIVSEDLScLk	S3, C5, C22	1	1	1									
Q8IAX2	Translation initiation factor SUI1, putative	kNEQQsAEDEEEYDEEDDNEEk	S6	1	1	1									
C6KTB3	Transportin	sDINkSDSNyIDENVYk	Y10	1	1	1									
Q8IKG9	Putative uncharacterized protein	eIFEGENtDDDTk	T8	1	1	1				1	1	2			
Q8IBU1	Zinc transporter, putative	nINsDIIIDENDIR	S4	1	1	1				2	1	2	1	1	
Q8ILQ4	Putative uncharacterized protein	eIsDDDDDNkGPk	S3	1	1	1	1	1	1						
Q8I1S7	RNA binding protein, putative	nNskENQPTTLLSNNDR	S3	1	1	1									
C0H5J9	Uncharacterized protein	kINsLVAEENLSk	S4	1	1	1									

Uniprot #	Protein Name	Peptide	Modification	Experiment 1			Experiment 2			Experiment 3			Experiment 4		
				Hep.	R1	Inv.	Hep.	R1	Inv.	Hep.	R1	Inv.	Hep.	R1	Inv.
Q8IM01	Putative uncharacterized protein	dREDEsNGAHLAEk	S6	1	1	1				1	1	1	2	1	2
Q8IKH3	26S proteasome subunit, putative	tAQDDNSSVLsDGk	S11	1	1	1					1	2			1
Q8IEE9	Cholinephosphate cytidylyltransferase	vHNsQDDmDNEEk	S4, M8	1	1	1									
O97247	T-complex protein beta subunit, putative	vNVHsFEDVQDLENEER	S5	1	1	1	1	1	1	1	1	1			1
Q8IJW2	Conserved Plasmodium protein	kETtDDEDEQQDQHLQQk	T4	1	1	1				2	1	2			
C0H4L9	Putative uncharacterized protein	nQNsDHNDDcIk	S4, C10	1	1	1	1	1	1	1		1	1	1	1
C6KTC6	Putative uncharacterized protein	eNskGsHNEESVSSTLGk	S3, S6	1	1	1							1		1
Q8I5R7	Proline--tRNA ligase	lLsNNEVEDNk	S3	1	1	1	1	1	1						
Q8I3T4	Transcriptional regulator, putative	fAYksDEDDEGYNk	S5	1	1	1	1	1	1	1	1	1	1	1	1
Q8IEM3	60S ribosomal protein L24, putative	aYVQsIEAk	S5	1	1	1	1	1	1						
C0H4L1	Putative uncharacterized protein	dVEANIkNsLEYNLR	S9	1	1	1	2	2	2	1	1	1			
Q7K6A0	cAMP-dependent protein kinase catalytic subunit	dSDsSEQVLTNk	S4	1	1	1									
		dSDSsEQVLTNk	S5				1	1	1						
		dSDSSEQVLtNk	T10										3	2	3
		kDSDsSEQVLTNk	S5							1		1		1	1
		kDSDSsEQVLTNk	S6										1	1	1
Q8IDZ0	Uncharacterized protein	nsTDNDLIFR	S2	1	1	1	1	1	1				1	1	
		nStDNDLIFR	T3												1
Q8ID52	Uncharacterized protein	iLIEsEDFISSR	S5	1	1	1				1	1	1			
Q8I611	Transporter, putative	nLsQEENk	S3	1	1	1									
Q8I2T2	Ubiquitin-like protein, putative	dDVsEIcDNEk	S4, C7	1	1	1							1	1	1
C6KT34	Cell division cycle protein 48 homologue, putative	gSsLGDGSGAGDR	S3	1	1	1	2	2	2	1	1	1			

Uniprot #	Protein Name	Peptide	Modification	Experiment 1			Experiment 2			Experiment 3			Experiment 4		
				Hep.	R1	Inv.	Hep.	R1	Inv.	Hep.	R1	Inv.	Hep.	R1	Inv.
O77388	HVA22/TB2/DP1 family protein, putative	iSsNVFGEk	S3	1	1	1									
Q8IAX1	Putative uncharacterized protein	yQQNEQNDGsPk	S10	1	1	1				1	1				
Q8IKV1	SNARE protein, putative	fNISsQEIEER	S5	1	1	1									
Q8IEC3	tRNA methyltransferase, putative	iDNSGEDEtYIYEEk	T9	1	1	1									
		iDNSGEDETyIYEEk	Y10					1	1						1
Q8IJX1	Conserved Plasmodium membrane protein	nATYsDDEIYQVNk	S5	1	1	1	2	2	2						
		nATYSDDEIyQVNk	Y10									1			
Q8I461	Cation transporting P-ATPase	ILsDDDLk	S3	1	1	1									
Q8IJE1	Conserved Plasmodium protein	vQDGsADEk	S5	1	1	1	1	1	1						
Q8IAW5	Putative uncharacterized protein	aLSyPVFDSNk	Y4	1	1	1									
		kSsYSQEk	S3										1	1	1
C6KTA7	Ankyrin-repeat protein, putative	nVHDsIEk	S5	1	1	1									
Q8ID31	Alanine--tRNA ligase, putative	eksIEENk	S3		1	1	1	1	1						
Q8I3A1	Replication factor A-related protein, putative	sLNDDTNDSk	S1	1	1	1	1	1	1						
		nILSNDVtQTPDk	T8										1	1	1
Q8IL48	tRNA binding protein, putative	sQNNEsPk	S6	1	1	1									
Q8II84	Conserved Plasmodium protein	sGsNNQENmEISk	S3, M9		1	1									
Q8IIF6	Conserved Plasmodium protein	vYsNENIIDTPNk	S3		1	1	1	1	1						
Q8IB66	RNA binding protein, putative	yNTYsISR	S5	1	1	1									
		yNNsNDAQGGNk	S4										1		1
Q8I5V5	Conserved Plasmodium protein	sNsQEVVk	S3	1	1	1	1	1	1						
Q8IIF0	Circumsporozoite-related antigen	gTGsGVSSk	S4	1	1	1									
Q8I468	Ser/Arg-rich splicing factor, putative	fDTSPsIVR	S6				1	1	1						
Q8IIS9	MIF4G domain containing protein	dNENNQEYDNNEDHNNNNNR	None					1	1	1	1	1			
		rLsGNVNNENmk	S3, M11										1	1	1

Uniprot #	Protein Name	Peptide	Modification	Experiment 1			Experiment 2			Experiment 3			Experiment 4		
				Hep.	R1	Inv.	Hep.	R1	Inv.	Hep.	R1	Inv.	Hep.	R1	Inv.
Q8IB24	Heat shock 70 kDa protein	gRLsQDEIDR	S4				5	5	5	1	1	1		1	3
		nENVDIIANDQGNR	None				1	1	1	1		1	3	2	5
		nsLENYcYGVk	S2, C7				1	1	1						
		nSLENycYGVk	Y6, C7							2	1	1	1		1
		ykAEDEENR	None				1	1	1	1	1	1	1	1	1
		lSQDEIDR	None										1		1
Q8I2W3	Nucleosome assembly protein	dRsENSVENtDPk	S3, T10				5	6	6						
		dRsENsVENTDPk	S3, S6				3	3	3			1	1	1	1
		dRSENsVENtDPk	S6, T10				2	2	2						
		dRSENsVENTDPk	S6				1	2	2						
		dRSENSVENtDPk	T10					1	1						
		sENsVENTDPk	S4				1	1	1						
		sENSVENtDPk	T8							2	2	3			
Q7KQK0	kinase regulatory subunit, putative	rLsVSAEAYGDWNk	S3				1	1	1						
		tDsEILDGLDYSEMSk	S3				1	1	1						
		tDsEILDGLDYSEmSk	S3, M14										1	1	1
		nILNDDGsSDGNDTDVHSmFDR	S8, M19							1	1	1			
		nILNDDGSsDGNDTDVHSMFDR	S9										1	1	1
		nILNDDGSsDGNDTDVHSmFDR	S9, M19										1	1	1
Q8I5I8	Glideosome-associated protein 45	eEIDYAtQENksFEEk	T7, S12				1	1	1	1		1	1	1	1
		eEIDYATQENksFEEk	S12							1	1	1	1	1	1
		eEIDYAtQENkSFEEk	T7										2		2
		sNSDIySESQk	Y6									1			
		sVTPcDMNk	S1, C5										1		1
Q8I374	Subunit of proteaseome activator complex, putative	gNNsSDFDER	S4				1	1	1						
		gNNssDFDEREk	S4, S5										3	3	3
Q8IKQ7	Putative uncharacterized protein	hTLHEEEtGEQk	T8				1	1	1	1	1	1	1		1
		htLHEEETGEQk	T2				1	1	1				1	1	1
Q8IM73	Putative uncharacterized protein	sSTLSQGNySNFk	Y9				1	2	2						
C0H4F1	Putative uncharacterized protein	lTskDLDDELDk	S3				1	1	1	1		1			
C0H4C7	Putative uncharacterized protein	eQYVQAHDsmEkEANNNS	S9, M10				1	1	1	1	1	1			
		eQYVQAHDsmEk	S9, M10							1	1	1			1

				Experiment 1			Experiment 2			Experiment 3			Experiment 4		
**Uniprot #**	**Protein Name**	**Peptide**	**Modification**	**Hep.**	**R1**	**Inv.**	**Hep.**	**R1**	**Inv.**	**Hep.**	**R1**	**Inv.**	**Hep.**	**R1**	**Inv.**
Q8I5F0	Conserved Plasmodium protein	ssSNDLVR	S2				1	1	1						
Q8I5D2	101 kDa malaria antigen	eTSQNQNENNDNQk	None						1						
Q8IJD0	Merozoite capping protein 1	gSVsSSNk	S4				2	2	2						
		kSsTSTQk	S3										1	1	1
O77309	Cytoadherence linked asexual protein 3.2	qTsTYIDTEk	S3				1	1	1						
		kQTsTYIDTEk	S4										1		1
		qTStYIDTEk	T4										1		1
		qGtEQLIDISk	T3											1	
Q8ILJ7	Phosphoenolpyruvate carboxylase, putative	iPsRNNVSTYDEATNk	S3				1	1	1						
Q8IBS3	Seryl-tRNA synthetase, putative	iSsQADIkDDTTcNSQk	S3, C13				1	1	1				1	1	1
		iSSQADIkDDtTcNSQk	T11, C13							1	1	1			
Q8IKF6	Putative uncharacterized protein	skNEsDNDDENDYk	S5				1	1	1	3	1	2	1	1	1
Q8I433	Rhomboid protease ROM4	scsINDDER	C2, S3				1	1	1						
O77344	Glycogen synthase kinase 3	sVSyIcSR	Y4, C6				1	1	1			1	1		1
C6KTE1	Putative uncharacterized protein	gNsFSNDEk	S3				1	1	1						
O77389	Formate-nitrate transporter, putative	dNNDAQmksLsIELR	M7, S9, S11				1	3	1				2	2	2
		dNNDAQMksLsIELR	S9, S11										3	1	1
Q8ILY2	Putative uncharacterized protein	sNSDNLNtLHEENk	T8							1	1	1	1		1
		rsNsDNLNTLHEENk	S2, S4							1	1	1	1	1	
		rsNSDNLNtLHEENk	S2, T9							1	1	1			
		rSNsDNLNtLHEENk	S4, T9										1	1	1
Q8IIB7	Ethanolamine kinase, putative	eIDEsEkEITQQR	S5							1	1		1	1	1
Q8IL42	Guanine nucleotide exchange factor, putative	yGGISsNDGEDGNIk	S6									1		2	1
		kEsDLHk	S3										1	1	1
C0H599	Plasmodium falciparum 3D7 chromosome 13	ekSsINDENDTk	S4							1	1	1	2	2	2
		eksSINDENDTk	S3							1	1	1			
Q8IC01	Cg4 protein	nNETNsPNkTELk	S6							1	1	1	1	1	2
		dQEQNDDkDQTNDNNmNEk	M16							1	1				
C6KSP3	Putative uncharacterized protein	sNTPSHHsFDNAk	S8							1	1	1	1	1	1
		sTsNcDEETR	S3, C5							1	1	1			

				Experiment 1			Experiment 2			Experiment 3			Experiment 4		
**Uniprot #**	**Protein Name**	**Peptide**	**Modification**	**Hep.**	**R1**	**Inv.**	**Hep.**	**R1**	**Inv.**	**Hep.**	**R1**	**Inv.**	**Hep.**	**R1**	**Inv.**
Q8IB48	Putative uncharacterized protein	kPNskEEEEINQPVk	S4							1	1	2			
Q8IDM6	Nucleoside transporter 1	eSskAYADIESR	S3							1	1	1			
Q8IEJ4	Uncharacterized protein	hSNsNNLHDkNEk	S4							1	1	2	1		1
		hSNsNNLHDk	S4												1
Q8I406	Phosphatidylinositol 4-kinase, putative	nLNENNsDDNkk	S7							1		1	1		1
O77363	Putative uncharacterized protein	qGPSHEsGDNLGk	S7									2	1	1	1
Q8IAV6	Putative uncharacterized protein	acNDNEEktENsSNEk	C2, T9, S12							1		2			
		acNDNEEktENSSNEk	C2, T9										1	1	1
C0H4K6	Ubiquitin transferase, putative	mYEEAHGGVsNDDEEGIQk	M1, S10							1	1	1	2	1	1
		mYEEAHGGVsNDDEEGIQk	S10										1	1	1
		nEVNLIsDHEk	S7							1	1	1	1		1
		nINsDkGEDSEk	S4										1		1
Q8ILZ1	Rhoptry-associated protein 1, RAP1	sASPHGESNsSEESTTk	S10							1	1		1	1	1
Q8I5L6	Clathrin heavy chain, putative	dNIFGNksDNENDEYLk	S8									1	1		1
Q8ILL5	Putative uncharacterized protein	kNtHEHEQk	T3							2	2	2			
		eQNNsNDILSk	S5										2	1	2
Q8I2Y5	RNA binding protein, putative	gDEEEHDEksLGEDNk	S10							1	1	1	1	1	1
		sVEEHNIDDk	S1										2	1	2
Q76NN8	Calcium-transporting ATPase, putative	eMksNENTIISR	S4								1	1			
Q7K6A5	Multidrug resistance protein	sDNNNNNNNDDNNNNNNNNNk	None							1	1	1			
Q8IDN4	Nucleic acid binding protein, putative	sDTkLENEIR	S1							1		1			
		ksGDEEEEEEEEEDEENNk	S2										1		
Q8I378	Putative uncharacterized protein	eEYHssDEENNkHNVk	S5, S6							1	1	1	1	1	2
Q8IJS3	Conserved Plasmodium protein	dNNDNNNNEk	None							1		1			1
Q8IAX8	DNA/RNA-binding protein Alba, putative	cGSTVITDQYVsGQDNSEHVVQEk	C1, S12							1	1	1			
		cGStVITDQYVSGQDNSEHVVQEk	C1, T4										1	1	1
		cGSTVITDQYVSGQDNsEHVVQEk	C1, S17										2	1	1

				Experiment 1			Experiment 2			Experiment 3			Experiment 4		
**Uniprot #**	**Protein Name**	**Peptide**	**Modification**	**Hep.**	**R1**	**Inv.**	**Hep.**	**R1**	**Inv.**	**Hep.**	**R1**	**Inv.**	**Hep.**	**R1**	**Inv.**
Q8IIC2	Neutral-sphingomyelinase activation factor protein, putative	eNINNTHVNsDEEk	S10							1	1	1	1	1	1
Q8I551	Conserved Plasmodium protein	iHNHRNsLDDDER	S7							1	1	1	1		
Q8I624	Conserved Plasmodium protein	kELsDEADNLNNIENNQNEk	S24								1		1		1
Q8IHX8	Conserved Plasmodium protein	gLLHEGsLsQEDHTNk	S7, S9									1	1	1	
Q8I1T4	Cyclin-dependent kinase, putative	eYNHNRsPSHAk	S7							1		1			
C6KSZ7	Putative uncharacterized protein	sGHHNDDQksVHsDDQk	S10, S13							1	1		1		
Q8IIW0	PfSNF2L	kLNssVEDGNDNNHDk	S4, S5							1	1	1			
		nIGNDTHVsYDDk	S9										1	1	1
C0H485	Regulator of initiation factor 2 (EIF2)	iNNQcEDNSEGDtHmEGk	C5, T13, M15							1		1			
		iNNQcEDNsEGDTHmEGk	C5, S9, M15										1	1	1
Q8ID51	Uncharacterized protein	hSTSENHtNTIEQTNEk	T8							1	1	1	1		1
H0YFX9	Histone H2A	nDEELNk	None									1			
Q8I1Z8	Putative uncharacterized protein	lLDLQEsRENQQNk	S7							1		1			
Q8IKE8	Putative uncharacterized protein	nQNSFVmNNEtNDHNENR	M7, T11							1	1	1			
		nQNsFVmNNETNDHNENR	S4, M7										1	1	1
Q8IDR9	40S ribosomal protein S6, putative	yRsELNQQHDVETTk	S3							1		1	1	1	1
Q8IJG6	Chromodomain-helicase-DNA-binding protein 1 homolog, putative	tGGGSLDNNNTsHQPmk	S12, M16							1	1	1			
Q8IL16	Putative uncharacterized protein	dLNEsPkNEPDIVYEEk	S5							1		1	1	1	1
Q8IEK1	M1-family aminopeptidase	lGSVVNNEENtcSDkR	T11, C12							1	1	1			
C6KSV4	Trancription factor, putative	nDNNNDDIsEHDAk	S9							1	1	1			
		kNDNNNDDIsEHDAk	S10										1		1

				Experiment 1			Experiment 2			Experiment 3			Experiment 4		
**Uniprot #**	**Protein Name**	**Peptide**	**Modification**	**Hep.**	**R1**	**Inv.**	**Hep.**	**R1**	**Inv.**	**Hep.**	**R1**	**Inv.**	**Hep.**	**R1**	**Inv.**
O97250	60S ribosomal protein L7, putative	yENQVENELGksmtSLR	S12, M13							1					
Q8IIX3	Early transcribed membrane protein 11.2, etramp11.2	tITATPk	None							1	1	1			
Q8IED6	Uncharacterized protein	qSHDsLQNk	S5							1		1	1	1	1
		IcLEDLRQsHDsLQNk	C2, S12										2	2	2
Q8IE57	Uncharacterized protein	dTDENIsDEHSR	S7							1	1	1		1	2
Q8IB72	DNAJ protein, putative	kASNDSYENsDVNGAVSk	S10							1	1	1			
Q8IJT3	Conserved Plasmodium protein	tSNSLLDNLsDR	S10									1			
		tSNsLLDNLSDR	S4												1
Q8IDC0	Zinc finger protein, putative	dNIVsSIDEEk	S5							1	1	1			
Q8I3A0	Cu2+-transporting ATPase, Cu2+ transporter	vEEsEGVDENRk	S4							1		1			
O96271	Replication factor C subunit 1, putative	tYAsDTIk	S4							1	1	1			
		nLFsDDEsDDGRk	S4, S8										1	1	1
Q8I2W4	Gamma-glutamylcysteine synthetase	vHNsNNNIHk	S4							1		1			
C0H583	Putative uncharacterized protein	rVPsSSEk	S4							1		1			
C0H4L6	Putative uncharacterized protein	kSsNDYEk	S3							1		1			
Q8IK09	Conserved Plasmodium protein	cNsRDESNEk	C1, S3							1		1			
Q8II83	Conserved Plasmodium protein	iSFVEAk	None							1	1	1			
Q8I0U6	DNAJ protein, putative	aSITNSAINyYDTVk	Y10											1	
		aSITNsAINYYDTVk	S6										1		
Q8I574	Splicing factor 3b, subunit 3, 130kD, putative	qINEELENENDDEDksDEER	S16										3	2	1
Q8I619	Conserved Plasmodium protein	sTTTYtSEDEYNISSk	T6										1		
		nNNDkVENANcsDNEk	C11, S12										1		1
Q8IFP1	U5 small nuclear ribonucleoprotein-specific protein, putative	eEDNDGGsGkEDNEPTQk	S8										2	2	2

				Experiment 1			Experiment 2			Experiment 3			Experiment 4		
Uniprot #	Protein Name	Peptide	Modification	Hep.	R1	Inv.	Hep.	R1	Inv.	Hep.	R1	Inv.	Hep.	R1	Inv.
Q8IKN7	Putative uncharacterized protein	ekEDDHSsSNVTNEVk	S8										1	2	2
Q9U0K8	Putative uncharacterized protein	dNHksEDNHk	S5										1	1	1
		sVEFyPPDINSk	Y5										1		1
Q8IJ28	Antigen UB05	dDAHkGDEDPNR	None										2	1	2
Q8I255	Putative uncharacterized protein	kNtEAsVNNNVEENYLR	T3, S6										1	1	2
Q8IJN7	Enolase	sAGYEGk	None										1		1
		tPNNDk	None										1		1
Q8I5S4	Bromodomain protein, putative	hGsNNsNINAHYEk	S3, S6										2	2	1
O97239	Protein dopey homolog PFC0245c	nFNSLtYEDk	T6										1		1
Q8I1Q1	Putative uncharacterized protein	sASNsVQIIEVDk	S5										1	1	1
Q7KWJ5	Hexose transporter, PfHT1	sskDIcSENEGk	S2, C6										1	1	1
Q8IJ99	Glycoprotease, putative	qTtNQGcNEk	T3, C7												1
C6KT67	Coronin binding protein	iSDSADEyNNSEk	Y8										1		1
Q8IJL2	Eukaryotic translation initiation factor subunit eIF2A, putative	lEEQNNTHLQHLySQQQR	Y13										1	1	1
O77334	Protein phosphatase	nLsRsVEISEk	S3, S5										1	1	1
Q8IKV6	Rhoptry neck protein 2	aHGSSNDYNAsNSQDk	S11										1	1	1
O77384	Protein PFC0760c	nNDGDNksQEDDDGNk	S8										1		1
Q8IC04	Heat shock protein 86 family protein	iYDGDEsEDDEGHDNk	S7										1		1
Q8IE99	Splicing factor, putative	aDTLkEEsDVsDNHHNNR	S8, S11										1		1
Q8I266	Lipid/sterol:H+ symporter	dGAIINNRPsSDEEYkEEk	S10										1	1	1
O97303	Putative uncharacterized protein	lEQDELkYsDDENEEQSYNk	S9										1		1
C0H584	Putative uncharacterized protein	nIGNINNsLENHPYNk	S8										1	1	1
Q9TY94	DEAD box helicase, UAP56	gSYATVHtGGFk	T8										1	1	1
C0H5J6	Sec24 subunit, putative	nIYSSSSsGNNVVDSk	S8										1	1	1
Q8IIV2	Histone H4	vFLENVIk	None												1
Q8IJ55	Merozoite surface protein 3	eQSNENNDQk	None												1

Uniprot #	Protein Name	Peptide	Modification	Experiment 1			Experiment 2			Experiment 3			Experiment 4		
				Hep.	R1	Inv.	Hep.	R1	Inv.	Hep.	R1	Inv.	Hep.	R1	Inv.
Q7KQK5	Apical membrane antigen 1, AMA1	sNsRNDEmLDPEASFWGEEk	S3, M8										1		1
Q8I537	Sphingomyelin phosphodiesterase, putative	rHDNYNDSSssTGQk	S10, S11										1	1	
Q8IB11	MAC/Perforin, putative	kVsIDLVDEEDDISNEk	S3										1		1
Q8I3K0	Putative uncharacterized protein	vEDGIHNHsLEk	S9										1	1	1
Q8IC05	Heat shock protein 86	lGIHEDNANR	None										1		1
Q8I2R3	Putative uncharacterized protein	gSINNDINsFER	S9												1
Q8IEG8	Uncharacterized protein	sAsHHNDDLEEk	S3										1	1	1
C6KT86	Phosphatidic acid phosphatase	hYNHkDsYSDNk	S7										1	1	1
Q8I5L1	Pre-mRNA splicing factor, putative	gYNDYNEsDIEREDESk	S8											1	1
O96205	Conserved Plasmodium protein	eGGSDNIYtSNIk	T9											1	1
Q8IHQ2	CCAAT-box DNA binding protein subunit B	eSINNINsDEk	S8										1		1
Q8IDL6	Elongation factor Tu, putative	dNVHINcNNsVEk	C7, S10										1	1	1
Q8IJR3	Conserved Plasmodium protein	tDSNIPDsNENVENk	S8											1	1
C0H4R3	Pseudouridylate synthase, putative	tVNNsNDDHk	S5										1		1
Q8IIW4	NOT family protein, putative	ecSEsLINTNk	C2, S5										1	1	1
Q8IL40	Putative uncharacterized protein	yNNNNskDDNTFNk	S6										1		1
Q8I320	Elongation factor 1-beta	gNSGTNtTTcTNk	T7, C10										1		
Q8I5M3	Conserved Plasmodium protein	tDSyENHmDLDk	Y4, M8										1	1	1
Q8IKG1	Putative uncharacterized protein	rEEISNsHEk	S7										1		1
Q8IHM7	DNAJ protein, putative	nDsNTNIQEk	S3										1		1
C0H4G1	Putative uncharacterized protein	nLDHccsIkR	C5, C6, S7										1	1	1

				Experiment 1			Experiment 2			Experiment 3			Experiment 4		
**Uniprot #**	**Protein Name**	**Peptide**	**Modification**	**Hep.**	**R1**	**Inv.**	**Hep.**	**R1**	**Inv.**	**Hep.**	**R1**	**Inv.**	**Hep.**	**R1**	**Inv.**
C0H564	Monocarboxylate transporter, putative	yNTNTDsNNIDIk	S7										1		1
Q8IHY0	Protein phosphatase 2C	sELTYDNLksMEEQEk	S10											1	1
Q8ID46	Uncharacterized protein	kNDDtDNEmEk	T5, M9										1	1	1
Q8II16	Coatomer delta subunit, putative	tYIEmEsHEEk	M5, S7											1	1
O77324	Putative uncharacterized protein	sDLPSSNIDEGsck	S12, C13										1		1
C6KSS2	G-protein associated signal transduction protein, putative	hSNtkEk	T4										1		1
Q8I5F9	Ubiquitin-activating enzyme E1, putative	qRtDEsVkPIEFDk	T3, S6										1	1	

Zuccala and Satchwell, Supplementary Table 5. Outlier peptides identified in assays where invasion was inhibited with heparin.

Exp.	Uniprot #	Protein	Peptide	Mod.	No.	A	B	C
**1**	B2RMN7	Beta Spectrin Erythrocytic	lLTSQDVsYDEAR	S8	1	49.98	5.523	5.744
	Q92508	Piezo-type mechanosensitive ion channel component 1	sGsEEAVTDPGER	S3	1	4.367	2.127	2.348
	P35580	Myosin 10	qLHLEGASLELsDDDTESk	S12	1	3.369	1.752	1.973
	Q5HYM4	Putative uncharacterized protein DKFZp686H1993	cDmSDLSLIGcLGysLLLMVtcTVYAIk	C1, M3, C11, Y14, S15, T21, C22	1	2.469	1.304	1.525
**2**	B2RMN7	Beta Spectrin Erythrocytic	lLTSQDVsYDEAR	S8	1	47.72	5.557	5.305
	A2RUB6	Coiled-coil domain-containing protein 66	syPGSQSQLFSQsTHk	S1, Y2, S13	1	38.77	5.277	5.005
	Q03001	Dystonin	stStQGLEHDLDDVNARWk	S1, T2, T4	1	24.08	4.59	4.319
	Q92508	Piezo-type mechanosensitive ion channel component 1	sGsEEAVTDPGER	S3	1	5.788	2.533	2.262
	Q59HD5	Sulfurtransferase	aRsPSVAAmASPQLcR	S3, M9, C15	1	3.891	1.960	1.689
	P02549	Alpha Spectrin Erythrocytic 1	gLAEVQNR	None	1	2.684	1.424	1.153
**3**	Q92508	Piezo-type mechanosensitive ion channel component 1	sGSEEAVtDPGER	T8	1	9.373	3.229	2.370
	P32119	Peroxiredoxin-2	qITVNDLPVGR	None	1	8.05	3.009	2.15
	P04921	Glycophorin C	gTEFAEsADAALQGDPALQDAGDSSR	S7	1	6.614	2.726	1.876
	Q7Z5Y0	EIF4B protein	sQSSDTEQQsPTSGGGk	S10	1	5.318	2.411	1.552
	B4DNV4	cDNA FLJ53071, highly similar to Heat shock 70 kDa protein 1*	aTAGDTHLGGEDFDNR	None	1	5.301	2.406	1.548
	I3L0W5	14-3-3 protein epsilon*	lAEQAER	None	1	4.383	2.132	1.273
	Q5T4S7	E3 ubiquitin-protein ligase UBR4	hAsTSSPADk	S3	1	3.944	1.980	1.121
	P16157	Ankyrin 1	rQDDATGAGQDsENEVSLVSGHQR/ qDDATGAGQDsENEVSLVSGHQR	S12/S11	6	3.404	1.767	0.909
	F5GWK0	Glucose 1,6-bisphosphate synthase	aVAGVmITAsHNR	M6, S10	1	3.244	1.698	0.839
	P02549	Alpha Spectrin Erythrocytic 1	gLAEVQNR	None	1	3.023	1.596	0.737
	P02549	Alpha Spectrin Erythrocytic 1	gTQLHEANQQLQFENNAEDLQR	None	1	2.959	1.565	0.706
	Q9UQ98	Multidrug resistance protein	hHNStAELQk	T5	1	2.948	1.560	0.701
**4**	Q9H8W4	Pleckstrin homology domain-containing family F member 2	sDSYSQsLk	S7	1	100	6.644	5.721
	Q4VB87	Protein 4.1	tQTVTIsDNANAVk	S7	1	60.88	5.928	5.005
	A5JTV0	MutL homolog 1	cAYRAsYSDGk	S6	1	46.48	5.539	4.615
	Q92508	Piezo-type mechanosensitive ion channel component 1	sGSEEAVtDPGER	T8	1	28.90	4.853	3.93
	Q9BZ23	Pantothenate kinase 2, mitochondrial	rAsSASVPAVGASAEGTR	S3	1	7.734	2.951	2.028
	P04921	Glycophorin C	gTEFAEsADAALQGDPALQDAGDSSR	S7	1	6.052	2.597	1.674
	F5GWK0	Glucose 1,6-bisphosphate synthase	aVAGVmITAsHNR	M6, S10	1	5.836	2.545	1.622
	Q7Z5Y0	EIF4B protein	sQSSDTEQQsPTSGGGk	S10	1	5.766	2.528	1.604
	P00915	Carbonic Anhydrase 1	gGPFSDSYR	None	1	5.275	2.399	1.476
	P16157	Ankyrin 1	rQDDATGAGQDsENEVSLVSGHQR/ qDDATGAGQDsENEVSLVSGHQR	S12/S11	5	3.76	1.911	0.988

Heparin outlier details including raw median fold change value (A), log2 (median fold change) value (B) and transformed log2 (median fold change) value (C) ranked within each experiment. Modifications on S, T and Y residues are phosphorylation events, modification of C residues is carbamidomethylation and modification of M residues is oxidation. Abbreviations: Experiment (Exp.), peptide modification (Mod.), number of peptide reads collected (No.). *These peptides share 100% sequence identity with *P. falciparum* proteins. B4DNV4 peptide is found in Pf 3D7 heat shock 70 kDa protein, Uniprot ID Q8IB24. I3L0W5 is found in putative Pf 3D7 14-3-3 protein, Uniprot ID C0H4V6.

Zuccala and Satchwell, Supplementary Table 6. Outlier peptides identified in assays where invasion was inhibited with R1.

Exp.	Uniprot #	Protein	Peptide	Mod.	No.	A	B	C
**1**	B2RMN7	Beta Spectrin Erythrocytic	lLTSQDVsYDEAR	S8	1	54.18	5.760	5.188
	Q92508	Piezo-type mechanosensitive ion channel component 1	sGsEEAVTDPGER	S3	1	6.108	2.611	2.039
	P16157	Ankyrin 1	iTHsPtVSQVTER	S4, T6	1	2.825	1.498	0.927
	Q4VB87	Protein 4.1	sLDGAAAVDSADR	S1	5	2.822	1.497	0.925
	H3BS66	Small integral membrane protein 1	dGVsLGAVsSTEEASR	S4, S9	1	2.797	1.484	0.912
	Q5HYM4	Putative uncharacterized protein DKFZp686H1993	cDmSDLSLIGcLGysLLLMVtcTVYAIk	C1, M3, C11, Y14, S15, T21, C22	1	2.589	1.372	0.801
	Q5H924	HECT, UBA and WWE domain containing 1	sHHAASTTTAPTPAAR	S1	1	2.570	1.361	0.790
	P02549	Alpha Spectrin Erythrocytic 1	gTQLHEANQQLQFENNAEDLQR	None	1	2.382	1.252	0.681
**2**	A2RUB6	Coiled-coil domain-containing protein 66	syPGSQSQLFSQsTHk	S1, Y2, S13	1	28.33	4.824	4.246
	Q03001	Dystonin	stStQGLEHDLDDVNARWk	S1, T2, T4	1	18.19	4.185	3.607
	B2RMN7	Beta Spectrin Erythrocytic	lLTSQDVsYDEAR	S8	1	11.82	3.563	2.985
	Q92508	Piezo-type mechanosensitive ion channel component 1	sGsEEAVTDPGER	S3	1	5.721	2.516	1.938
	Q59HD5	Sulfurtransferase	aRsPSVAAmASPQLcR	S3, M9, C15	1	3.318	1.730	1.152
	P02549	Alpha Spectrin Erythrocytic 1	gLAEVQNR	None	1	2.729	1.448	0.870
**3**	Q92508	Piezo-type mechanosensitive ion channel component 1	sGSEEAVtDPGER	T8	1	8.322	3.057	2.479
	P32119	Peroxiredoxin-2	qITVNDLPVGR	None	1	8.191	3.034	2.456
	Q7Z5Y0	EIF4B protein	sQSSDTEQQsPTSGGGk	S10	1	7.721	2.949	2.371
	P04921	Glycophorin C	gTEFAEsADAALQGDPALQDAGDSSR	S7	1	5.916	2.565	1.987
	B4DNV4	cDNA FLJ53071, highly similar to Heat shock 70 kDa protein 1*	aTAGDTHLGGEDFDNR	None	1	5.516	2.464	1.886
	Q5T4S7	E3 ubiquitin-protein ligase UBR4	hAsTSSPADk	S3	1	3.764	1.912	1.334
	I3L0W5	14-3-3 protein epsilon*	lAEQAER	None	1	3.589	1.844	1.266
	Q9UQ98	Multidrug resistance protein	hHNStAELQk	T5	1	2.979	1.575	0.997
	Q9NZD4	Alpha-hemoglobin-stabilizing protein	qQVTGEPQER	None	1	2.879	1.526	0.948
**4**	Q4VB87	Protein 4.1	tQTVTIsDNANAVk	S7	1	100	6.644	5.760
	Q9H8W4	Pleckstrin homology domain-containing family F member 2	sDSYSQsLk	S7	1	100	6.644	5.760
	A5JTV0	MutL homolog 1	cAYRAsYSDGk	S6	1	100	6.644	5.760
	Q92508	Piezo-type mechanosensitive ion channel component 1	sGSEEAVtDPGER	T8	1	35.705	5.158	4.274
	Q9BZ23	Pantothenate kinase 2, mitochondrial	rAsSASVPAVGASAEGTR	S3	1	9.691	3.277	2.393
	P04921	Glycophorin C	gTEFAEsADAALQGDPALQDAGDSSR	S7	1	6.532	2.708	1.824
	P00915	Carbonic Anhydrase 1	gGPFSDSYR	None	1	5.578	2.480	1.596
	F5GWK0	Glucose 1,6-bisphosphate synthase	aVAGVmITAsHNR	M6, S10	1	5.444	2.445	1.561
	Q7Z5Y0	EIF4B protein	sQSSDTEQQsPTSGGGk	S10	1	4.590	2.199	1.315
	P16157	Ankyrin 1	rQDDATGAGQDsENEVSLVSGHQR/ qDDATGAGQDsENEVSLVSGHQR	S12/S11	5	3.961	1.986	1.102
	P16157	Ankyrin 1	iTHSPtVsQVTER	T6, S8	1	3.573	1.837	0.954

R1 outlier details including raw median fold change value (A), log2 (median fold change) value (B) and transformed log2 (median fold change) value (C) ranked within each experiment. Modifications on S, T and Y residues are phosphorylation events, modification of C residues is carbamidomethylation and modification of M residues is oxidation. Abbreviations: Experiment (Exp.), peptide modification (Mod.), number of peptide reads collected (No.). *These peptides share 100% sequence identity with *P. falciparum* proteins. B4DNV4 peptide is found in Pf 3D7 heat shock 70 kDa protein, Uniprot ID Q8IB24. I3L0W5 is found in putative Pf 3D7 14-3-3 protein, Uniprot ID C0H4V6.

Zuccala and Satchwell, Supplementary Table 7. Outlier peptides identified in assays where invasion was not inhibited.

Exp.	Uniprot #	Protein	Peptide	Mod.	No.	A	B	C
**1**	B2RMN7	Beta Spectrin Erythrocytic	lLTSQDVsYDEAR	S8	1	42.33	5.404	5.227
	Q92508	Piezo-type mechanosensitive ion channel component 1	sGsEEAVTDPGER	S3	1	3.161	1.660	1.484
	P35580	Myosin 10	qLHLEGASLELsDDDTESk	S12	1	2.320	1.214	1.037
	Q5H924	HECT, UBA and WWE domain containing 1	sHHAASTTTAPTPAAR	S1	1	2.311	1.209	1.032
	P16157	Ankyrin 1	iTHsPtVSQVTER	S4, T6	1	2.297	1.200	1.023
	Q5HYM4	Putative uncharacterized protein DKFZp686H1993	cDmSDLSLIGcLGysLLLMVtcTVYAIk	C1, M3, C11, Y14, S15, T21, C22	1	2.296	1.199	1.022
**2**	B2RMN7	Beta Spectrin Erythrocytic	lLTSQDVsYDEAR	S8	1	35.33	5.143	4.586
	A2RUB6	Coiled-coil domain-containing protein 66	syPGSQSQLFSQsTHk	S1, Y2, S13	1	18.58	4.261	3.659
	Q03001	Dystonin	stStQGLEHDLDDVNARWk	S1, T2, T4	1	15.49	3.954	3.397
	Q92508	Piezo-type mechanosensitive ion channel component 1	sGsEEAVTDPGER	S3	1	4.743	2.246	1.689
	P68871	Hemoglobin subunit beta	vLGAFSDGLAHLDNLk	None	5	3.643	1.865	1.308
	P02549	Alpha Spectrin Erythrocytic 1	gLAEVQNR	None	1	2.962	1.567	1.010
	P04921	Glycophorin C	gTEFAESADAALQGDPALQDAGDSSR	None	2	2.796	1.484	0.926
	Q59HD5	Sulfurtransferase	aRsPSVAAmASPQLcR	S3, M9, C15	1	2.667	1.415	0.858
	P02549	Alpha Spectrin Erythrocytic 1	gTQLHEANQQLQFENNAEDLQR	None	1	2.361	1.239	0.682
**3**	B2RMN7	Beta Spectrin Erythrocytic	lLTSQDVsYDEAR	S8	1	93.55	6.548	6.055
	Q4VB87	Protein 4.1	tQTVtISDNANAVk	T5	1	76.30	6.254	5.761
	Q92508	Piezo-type mechanosensitive ion channel component 1	sGSEEAVtDPGER	T8	1	6.183	2.628	2.136
	P32119	Peroxiredoxin-2	qITVNDLPVGR	None	1	5.503	2.460	1.968
	P04921	Glycophorin C	gTEFAEsADAALQGDPALQDAGDSSR	S7	1	4.208	2.073	1.581
	Q7Z5Y0	EIF4B protein	sQSSDTEQQsPTSGGGk	S10	1	4.197	2.069	1.577
	Q5T4S7	E3 ubiquitin-protein ligase UBR4	hAsTSSPADk	S3	1	3.288	1.717	1.225
	I3L0W5	14-3-3 protein epsilon*	lAEQAER	None	1	3.098	1.631	1.139
	Q9NZD4	Alpha-hemoglobin-stabilizing protein	qQVTGEPQER	None	1	3.038	1.603	1.111
	B4DNV4	cDNA FLJ53071, highly similar to Heat shock 70 kDa protein 1*	aTAGDTHLGGEDFDNR	None	1	2.760	1.465	0.972
	P16157	Ankyrin 1	dSGEGDTTSLR	None	1	2.590	1.373	0.881
	P02549	Alpha Spectrin Erythrocytic 1	gTQLHEANQQLQFENNAEDLQR	None	1	2.502	1.323	0.831
	Q9UQ98	Multidrug resistance protein	hHNStAELQk	T5	1	2.496	1.319	0.827
**4**	B2RMN7	Beta Spectrin Erythrocytic	lLTSQDVSyDEAR	Y9	1	78.30	6.291	5.537
	Q4VB87	Protein 4.1	tQTVTIsDNANAVk	S7	1	77.91	6.284	5.530
	A5JTV0	MutL homolog 1	cAYRAsYSDGk	S6	1	64.21	6.005	5.251
	Q9H8W4	Pleckstrin homology domain-containing family F member 2	sDSYSQsLk	S7	1	26.03	4.702	3.948
	Q92508	Piezo-type mechanosensitive ion channel component 1	sGSEEAVtDPGER	T8	1	11.48	3.521	2.767
	Q9BZ23	Pantothenate kinase 2, mitochondrial	rAsSASVPAVGASAEGTR	S3	1	5.390	2.430	1.676
	P04921	Glycophorin C	gTEFAEsADAALQGDPALQDAGDSSR	S7	1	4.548	2.185	1.431
	P00915	Carbonic Anhydrase 1	gGPFSDSYR	None	1	4.423	2.145	1.391
	Q7Z5Y0	EIF4B protein	sQSSDTEQQsPTSGGGk	S10	1	4.185	2.065	1.311
	P16157	Ankyrin 1	rQDDATGAGQDsENEVSLVSGHQR/ qDDATGAGQDsENEVSLVSGHQR	S12/S11	5	3.266	1.708	0.953

Invasion outlier details including raw median fold change value (A), $\log_2$ (median fold change) value (B) and transformed $\log_2$ (median fold change) value (C) ranked within each experiment. Modifications on S, T and Y residues are phosphorylation events, modification of C residues is carbamidomethylation and modification of M residues is oxidation. Abbreviations: Experiment (Exp.), peptide modification (Mod.), number of peptide reads collected (No.). *These peptides share 100% sequence identity with *P. falciparum* proteins. B4DNV4 peptide is found in Pf 3D7 heat shock 70 kDa protein, Uniprot ID Q8IB24. I3L0W5 is found in putative Pf 3D7 14-3-3 protein, Uniprot ID C0H4V6.

Zuccala and Satchwell, Supplementary Table 8. Outlier phospho-peptides unique to one or two assay conditions.

Conditions	Exp.	Protein	Peptide	Mod.	Detected in other Conditions?
**R1**	1	Protein 4.1	SLDGAAAVDSAD R	S1	Hep. (1-4), R1 (2-4), Inv. (1-4)
**R1**	1	Small integral membrane protein 1	DGVsLGAVsSTE EASR	S4, S9	Hep. (1), R1 (4), Inv. (1,4)
**R1 & Invasion**	1 & 4	Ankyrin 1	ITHsPtVSQVTER	S4, T6 or T6, S8	Hep. (1-3), R1 (2,3), Inv. (2,3)
**R1 & Invasion**	1	HECT, UBA and WWE domain containing 1	SHHAASTTTAPT PAAR	S1	Hep. (1,3), R1 (3), Inv. (3)

Four outlier phospho9peptides were found exclusively in either R1 assays or R1 and invasion (uninhibited) assays. These peptides were also detected in other experiments and assay conditions. Abbreviations: Peptide modifications (Mod.), heparin (Hep.), uninhibited invasion (Inv.), experiment (Exp.).

Zuccala and Satchwell, Supplementary Table 9. Summary of outliers identified across all four experiments.

Exp.	Condition	Outlier Proportion	Phospho-Peptide Proportion	Proportion of Outliers that are Phospho-Peptides
**1**	Heparin	4/116 (3.5%)	70/116 (60.3%)	4/4 (100%)
	R1	8/114 (7%)	69/114 (60.5%)	7/8 (87.5%)
	Invasion	6/114 (5.3%)	70/114 (61.4%)	6/6 (100%)
**2**	Heparin	6/95 (6.3%)	55/95 (57.9%)	5/6 (83.3%)
	R1	6/95 (6.3%)	55/95 (57.95%)	5/6 (83.3%)
	Invasion	9/94 (9.6%)	55/94 (58.5%)	5/9 (55.6%)
**3**	Heparin	12/134 (9.0%)	61/134 (45.5%)	7/12 (58.3%)*
	R1	9/134 (6.7%)	61/134 (45.5%)	5/9 (55.6%)*
	Invasion	13/136 (9.6%)	62/134 (46.3%)	6/13 (46.2%)*
**4**	Heparin	10/112 (8.9%)	57/112 (50.9%)	9/10 (90.0%)
	R1	11/111 (9.9%)	55/111 (49.5%)	10/11 (90.9%)
	Invasion	10/113 (8.9%)	58/113 (51.3%)	9/10 (90.0%)

Outlier proportions across experiments and conditions ranged from 3.5% 9 9% of all erythrocyte peptides. While the proportion of phospho9peptides in each assay ranged from around 45% to around 60%, the proportion of outliers that were phosphorylated was much higher, indicating the outlier identification method is robust. Abbreviations: Experiment (Exp.). *Assays where some non9phosphorylated outlier peptides shared 100% sequence identity with *P. falciparum* proteins.

Zuccala and Satchwell, Supplementary Table 10. Confidence measures of phospho-site assignment for shortlisted peptides.

		Average SEQUEST delta Cn Score				
**Protein**	**Peptide**	**Mod.**	**Exp.1**	**Exp. 2**	**Exp. 3**	**Exp. 4**
Beta Spectrin	ILTSQDVsYDEAR	S8	0.58	0.56	0.09**	ND
Beta Spectrin	ILTSQDVSyDEAR	Y9	ND	ND	ND	0.00*
PIEZO1	SGsEEAVTDPGER	S3	0.63	0.52	ND	ND
PIEZO1	SGSEEAVtDPGER	T8	ND	ND	0.29	0.14
Glycophorin C	GTEFAEsADAALQGDPALQDAGDSSR	S7	0.32	ND	0.32	0.35
Protein 4.1	SQSSDTEQQsPTSGGGk	T5	ND	ND	0.05**	ND
Protein 4.1	SQSSDtEQQSPTSGGGk	S7			ND	0.02**
EIF4B Protein	TQTVtISDNANAVK	S10	0.10	ND	0.14	ND
EIF4B Protein	TQTVTIsDNANAVK	T6	ND	ND	ND	0.11
Ankyrin 1	RQDDATGAGQDsENEVSLVSGHQR/QDDA TGAGQDsENEVSLVSGHQR	S12/S11	0.56	0.58	0.35	0.32
Ankyrin 1	ITHsPtVSQVTER	S4, T6	0.59	0.52	0.11	ND
Ankyrin 1	ITHSPtVsQVTER	T6, S8	ND	0.15	ND	0.01**

For each experiment, the average SEQUEST delta Cn score was calculated for outlier phospho-peptides and those peptides with the same sequence and an alternative closely located phosphorylated residue. * Manual inspection of peptide fragmentation ions indicated site assignment is ambiguous. **Manual inspection reveals good evidence for selected phospho-site. Abbreviations: Mod. = modification site within peptide, Exp. = experiment, ND = peptide not detected.

Zuccala and Satchwell, Supplementary Table 11. Previous reports of shortlisted outlier phosphorylated residues.

Protein	Site	Site Previously Identified?	Cell Type/Tissue
Beta Spectrin	S1301	1	*P. falciparum* infected erythrocyte
Beta Spectrin	Y1302	No	
PIEZO1	S1621	2-5	HeLa (cervical), K562 (erythroid)
PIEZO1	T1626	4, 6	HeLa (cervical), leukocyte (blood)
Glycophorin C	S104	7	Jurkat (T lymphocyte)
EIF4B protein	S504	4, 8-23	293 (epithelial), HUES9 (hESC), 293E (epithelial), breast, fibroblast-skin, Flp-In T-Rex-293 (epithelial), HeLa (cervical), HeLa S3 (cervical), HT-29 (intestinal), Jurkat (T lymphocyte), K562 (erythroid), liver, MKN-45 (gastric)
Protein 4.1	T738	9	HUES9 (hESC)
Protein 4.1	S740	No	
Ankyrin	S1666	1, 3, 11, 12, 18, 24	*P. falciparum* infected erythrocyte, skeletal muscle, Flip-In T-Rex-293 (epithelial), K562 (erythroid), liver
Ankyrin	S1686	1-3, 5, 10, 18, 25-31	*P. falciparum* infected erythrocyte, skeletal muscle, HEL (erythroid), HeLa (cervical), hepatocyte (liver), K562 (erythroid), liver, MV4-11 (macrophage), P31/FUJ (myocyte), platelet (blood), T lymphocyte (blood)
Ankyrin	T1688	No	

Most identified outlier phospho-sites present in at least two experiments have been reported in the literature, with the exception of beta-spectrin p-Y1302 and protein 4.1 p-S740, where no previous reports could be found.

Table References

1. Solyakov, L. et al. Global kinomic and phospho-proteomic analyses of the human malaria parasite Plasmodium falciparum. *Nat Commun* **2**, 565 (2011).
2. Shiromizu, T. et al. Identification of missing proteins in the neXtProt database and unregistered phosphopeptides in the PhosphoSitePlus database as part of the Chromosome-centric Human Proteome Project. *J Proteome Res* **12**, 2414-21 (2013).
3. Zhou, H. et al. Toward a comprehensive characterization of a human cancer cell phosphoproteome. *J Proteome Res* **12**, 260-71 (2013).
4. Kettenbach, A.N. et al. Quantitative phosphoproteomics identifies substrates and functional modules of Aurora and Polo-like kinase activities in mitotic cells. *Sci Signal* **4**, rs5 (2011).
5. Phanstiel, D.H. et al. Proteomic and phosphoproteomic comparison of human ES and iPS cells. *Nat Methods* **8**, 821-7 (2011).
6. Raijmakers, R., Kraiczek, K., de Jong, A.P., Mohammed, S. & Heck, A.J. Exploring the human leukocyte phosphoproteome using a microfluidic reversed-phase-TiO2-reversed-phase high-performance liquid chromatography phosphochip coupled to a quadrupole time-of-flight mass spectrometer. *Anal Chem* **82**, 824-32 (2010).
7. Mayya, V. et al. Quantitative phosphoproteomic analysis of T cell receptor signaling reveals system-wide modulation of protein-protein interactions. *Sci Signal* **2**, ra46 (2009).
8. Olsen, J.V. et al. Quantitative phosphoproteomics reveals widespread full phosphorylation site occupancy during mitosis. *Sci Signal* **3**, ra3 (2010).
9. Rigbolt, K.T. et al. System-wide temporal characterization of the proteome and phosphoproteome of human embryonic stem cell differentiation. *Sci Signal* **4**, rs3 (2011).
10. Sharma, K. et al. Ultradeep human phosphoproteome reveals a distinct regulatory nature of Tyr and Ser/Thr-based signaling. *Cell Rep* **8**, 1583-94 (2014).
11. Bian, Y. et al. An enzyme assisted RP-RPLC approach for in-depth analysis of human liver phosphoproteome. *J Proteomics* **96**, 253-62 (2014).
12. Franz-Wachtel, M. et al. Global detection of protein kinase D-dependent phosphorylation events in nocodazole-treated human cells. *Mol Cell Proteomics* **11**, 160-70 (2012).
13. Weber, C., Schreiber, T.B. & Daub, H. Dual phosphoproteomics and chemical proteomics analysis of erlotinib and gefitinib interference in acute myeloid leukemia cells. *J Proteomics* **75**, 1343-56 (2012).
14. Grosstessner-Hain, K. et al. Quantitative phospho-proteomics to investigate the polo-like kinase 1-dependent phospho-proteome. *Mol Cell Proteomics* **10**, M111 008540 (2011).
15. Jernigan, C.S. et al. The mTOR signaling pathway in the prefrontal cortex is compromised in major depressive disorder. *Prog Neuropsychopharmacol Biol Psychiatry* **35**, 1774-9 (2011).
16. Hsu, P.P. et al. The mTOR-regulated phosphoproteome reveals a mechanism of mTORC1-mediated inhibition of growth factor signaling. *Science* **332**, 1317-22 (2011).
17. Christensen, G.L. et al. Quantitative phosphoproteomics dissection of seven-transmembrane receptor signaling using full and biased agonists. *Mol Cell Proteomics* **9**, 1540-53 (2010).
18. Pan, C., Olsen, J.V., Daub, H. & Mann, M. Global effects of kinase inhibitors on signaling networks revealed by quantitative phosphoproteomics. *Mol Cell Proteomics* **8**, 2796-808 (2009).
19. Gauci, S. et al. Lys-N and trypsin cover complementary parts of the phosphoproteome in a refined SCX-based approach. *Anal Chem* **81**, 4493-501 (2009).
20. Beausoleil, S.A., Villen, J., Gerber, S.A., Rush, J. & Gygi, S.P. A probability-based approach for high-throughput protein phosphorylation analysis and site localization. *Nat Biotechnol* **24**, 1285-92 (2006).
21. Beausoleil, S.A. et al. Large-scale characterization of HeLa cell nuclear phosphoproteins. *Proc Natl Acad Sci U S A* **101**, 12130-5 (2004).
22. Yang, F. et al. Phosphoproteome profiling of human skin fibroblast cells in response to low- and high-dose irradiation. *J Proteome Res* **5**, 1252-60 (2006).
23. Kim, J.E., Tannenbaum, S.R. & White, F.M. Global phosphoproteome of HT-29 human colon adenocarcinoma cells. *J Proteome Res* **4**, 1339-46 (2005).
24. Wu, Y. et al. Identification of phosphorylated proteins in erythrocytes infected by the human malaria parasite Plasmodium falciparum. *Malar J* **8**, 105 (2009).
25. Alcolea, M.P., Casado, P., Rodriguez-Prados, J.C., Vanhaesebroeck, B. & Cutillas, P.R. Phosphoproteomic analysis of leukemia cells under basal and drug-treated conditions identifies markers of kinase pathway activation and mechanisms of resistance. *Mol Cell Proteomics* **11**, 453-66 (2012).
26. Ruperez, P., Gago-Martinez, A., Burlingame, A.L. & Oses-Prieto, J.A. Quantitative phosphoproteomic analysis reveals a role for serine and threonine kinases in the cytoskeletal reorganization in early T cell receptor activation in human primary T cells. *Mol Cell Proteomics* **11**, 171-86 (2012).
27. Han, G. et al. Phosphoproteome analysis of human liver tissue by long-gradient nanoflow LC coupled with multiple stage MS analysis. *Electrophoresis* **31**, 1080-9 (2010).
28. Hojlund, K. et al. In vivo phosphoproteome of human skeletal muscle revealed by phosphopeptide enrichment and HPLC-ESI-MS/MS. *J Proteome Res* **8**, 4954-65 (2009).
29. Lee, H.J. et al. Quantitative analysis of phosphopeptides in search of the disease biomarker from the hepatocellular carcinoma specimen. *Proteomics* **9**, 3395-408 (2009).
30. Carrascal, M., Ovelleiro, D., Casas, V., Gay, M. & Abian, J. Phosphorylation analysis of primary human T lymphocytes using sequential IMAC and titanium oxide enrichment. *J Proteome Res* **7**, 5167-76 (2008).
31. Zahedi, R.P. et al. Phosphoproteome of resting human platelets. *J Proteome Res* **7**, 526-34 (2008).

Zuccala and Satchwell, Supplementary Table 12. Outlier peptides identified in assays where erythrocytes were incubated with microbeads.

Experiment & Condition	Uniprot #	Protein Name	Peptide	Mod.	No.	A	B	C
**1. Beads + BlockAid**	Q9C0C9	Ubiquitin-conjugating enzyme E2 O	IIHGEDsDsEG EEEGR	S7, S9	2	1.738	0.797	0.954
	E7EV99	Alpha-adducin	AAVVtSPPPT TAPHK	T5	1	1.493	0.578	0.734
	E5RFE7	Carbonic anhydrase 1	ySSLAEAASk	None	1	1.359	0.442	0.599
	E7EV99	Alpha-adducin	AAVVTsPPPT TAPHK	S6	3	1.335	0.417	0.574
	P02042	Hemoglobin subunitdelta	VNVDAVGGE ALGR	None	1	1.198	0.261	0.418
	K7EM20	14-3-3 protein epsilon	VLAEFATGND R	None	2	1.169	0.226	0.382
**1. Beads + WGA**	P07900	Heat shock protein HSP 90-alpha	ESEDkPEIED VGsDEEEEK	S13	2	0.432	-1.209	-1.05
	P07900	Heat shock protein HSP 90-alpha	DKEVsDDEAE EK	S5	1	0.504	-0.987	-0.831
	Q6J1Z8	Hemoglobin beta	ALAHK	None	2	0.543	-0.880	-0.724
**2. Beads + BlockAid**	Q9C0C9	Ubiquitin-conjugating enzyme E2 O	IIHGEDsDsEG EEEGR	S7, S9	2	2.178	1.123	1.17
	P02042	Hemoglobin subunitdelta	VNVDAVGGE ALGR	None	1	1.418	0.504	0.498
	E7EV99	Alpha-adducin	AAVVTsPPPT TAPHK	S6	3	1.387	0.472	0.467
	E5RFE7	Carbonic anhydrase 1	YSSLAEAASK	None	1	1.378	0.463	0.457
	Q14476	G-gamma-hemoglobin gene from Greek HPFH mutant	HLDDLk	None	1	1.240	0.310	0.304
	K7EPV9	Tropomyosin alpha-4 chain	HIAEEADR	None	1	0.751	-0.414	-0.419
**2. Beads + WGA**	P07900	Heat shock protein HSP 90-alpha	ESEDkPEIED VGsDEEEEK	S13	2	0.519	-0.947	-0.904
	P07900	Heat shock protein HSP 90-alpha	DKEVsDDEAE EK	S5	1	0.574	-0.801	-0.758

Outlier details including raw median fold change value (A), log_2 (median fold change) value (B) and transformed log_2 (median fold change) value (C) ranked within each experiment. Modifications on S and T residues are phosphorylation events. Abbreviations: Experiment (Exp.), peptide modification (Mod.), number of peptide reads collected (No.).

Zuccala and Satchwell Supplementary Table 13: Shortlisted erythrocyte outlier phospho-peptides from invasion proteomics were not phosphorylated in response to microbeads.

Protein	Peptide	Mod.	Detected in Bead + Blockaid Assays?	Detected in Bead + WGA Assays?
Beta Spectrin	ILTSQDVSYDEAR	S8 or Y9	No: Non-phosphorylated peptide detected	No: Non-phosphorylated peptide detected
PIEZO1	SGSEEAVTDPGER	S3 or T8	Yes (T8): Non an outlier	Yes (T8): Non an outlier
Glycophorin C	GTEFAESADAALQGDPAL QDAGDSSR	S7	No	No
EIF4B protein	SQSSDTEQQSPTSGGGK	S10	Yes: Not an outlier	Yes: Not an outlier
Protein 4.1	TQTVTISDNANAVK	T5 or S7	No	No
Ankyrin	RQDDATGAGQDSENEVS LVSGHQR/ GDDATGAGQDsENEVSL VSGHQR	S12/S11	Yes: Not an outlier	Yes: Not an outlier
Ankyrin	ITHsPtVSQVTER	S4 & T6 or T6 & S8	No: Only single phosphorylation events detected	No: Only single phosphorylation events detected

Zuccala and Satchwell Supplementary Table 14: Top kinase predictions for shortlisted invasion phospho-peptides using NetPhosK

Protein	Peptide	Identified Sites	Top Kinase Predictions
Beta Spectrin	ILTSQDVSYDEAR	S8 (S1301) or Y9 (Y1302)	S1301 – CKII (0.66)<br>S1297 – ATM (0.61)<br>S1297 – DNAPK (0.53)<br>Y1302 – INSR (0.48)
PIEZO1	sGSEEAVTDPGER	S3 (S1621) or T8 (T1626)	S1619 – CKII (0.61)<br>T1626 – CKII (0.60)<br>S1619 – cdc2 (0.58)<br>T1626 – cdc2 (0.48)<br>S1621 – GSK3/CAM-II (0.45)*
Glycophorin C	gTEFAESADAALQGDPALQDAGDS SR	S7 (S104)	S122 – CKII (0.58)<br>S122 – PKG (0.54)<br>T99 – CKII (0.53)<br>S104 – CaM-II (0.48)
EIF4B protein	sQSSDTEQQSPTSGGGk	S10 (S504)	S497 – PKA (0.64)<br>S495 – DNAPK (0.6)<br>S504 – cdk5 (0.56)<br>T506 – CKI (0.55)
Protein 4.1	tQTVTISDNANAVk	T5 (T738) or S7 (S740)	T736 – PKC (0.56)<br>T734 –PKG/DNAPK (0.47)<br>T738 – CaM-II (0.46)<br>T736 – CaM II (0.45)<br>S740 – CaM II (0.44)*
Ankyrin 1	rQDDATGAGQDSENEVSLVSGHQR / qDDATGAGQDsENEVSLVSGHQR	S12/S11 (S1666)	S1674 – PKC (0.75)<br>S1666 – CKII (0.67)<br>S1674 – CaM-II (0.46)<br>S1666 – CaM-II (0.45)
Ankyrin 1	iTHsPtVSQVTER	S4 & T6 (S1686 & T 1688) or T6 & S8 (T1688 & S1690)	S1686 – p38MAPK (0.58)<br>S1690 – DNAPK (0.56)<br>S1686 – GSK3 (0.5)<br>S1690 – ATM (0.5)<br>T1688 – CaM-II (0.45)*

Top kinase predictions for sites located within invasion assay outlier phospho-peptides. Predictions highlighted in blue are top matches for the phosphorylated residues identified by mass spectrometry. Modifications on S and T residues are phosphorylation events. * phospho-site does not represent next most likely in the list.

Zuccala and Satchwell Figure S1

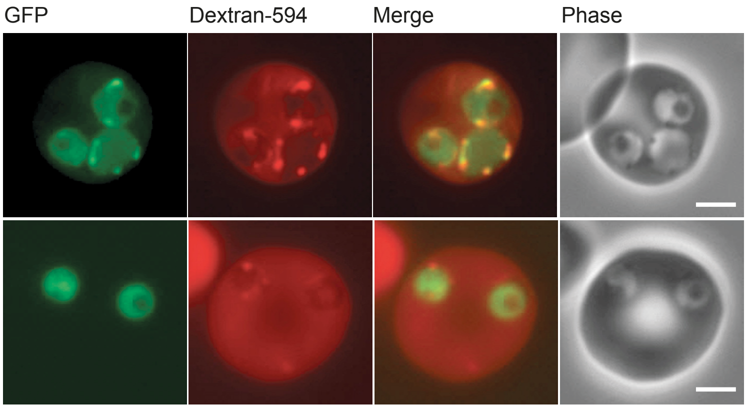

Zuccala and Satchwell Figure S2

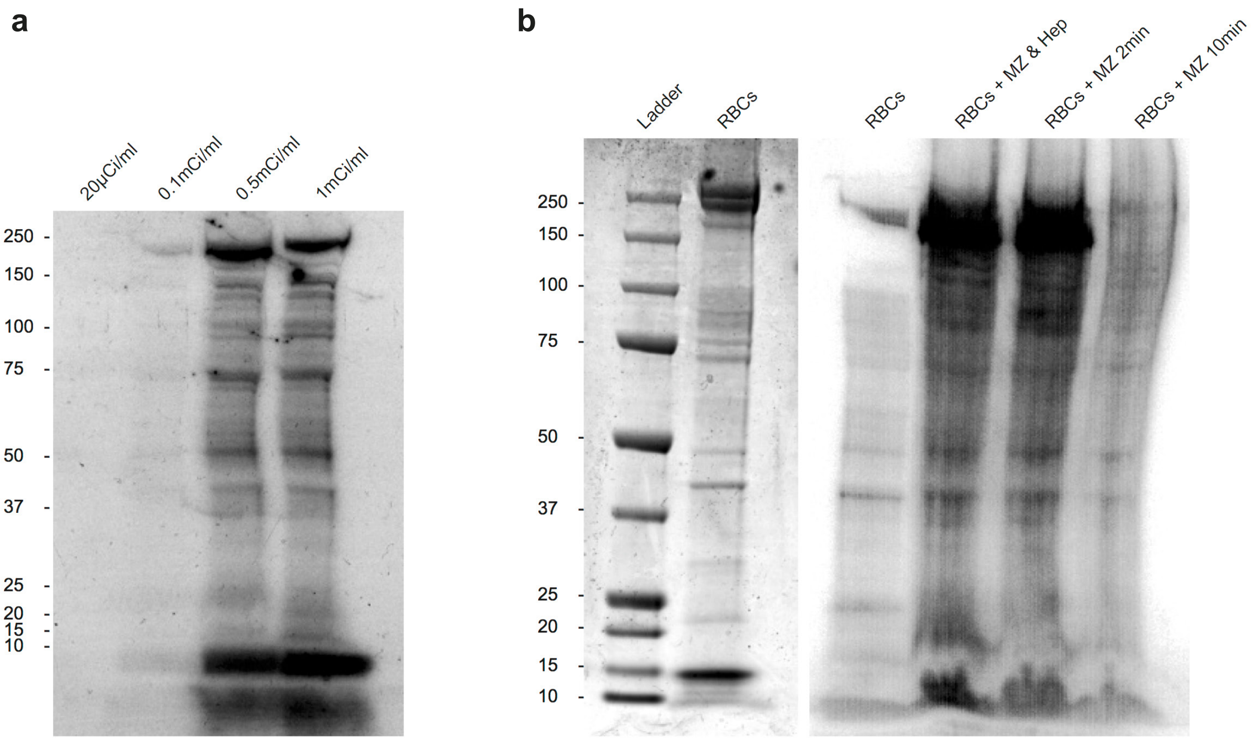

Zuccala and Satchwell Figure S3

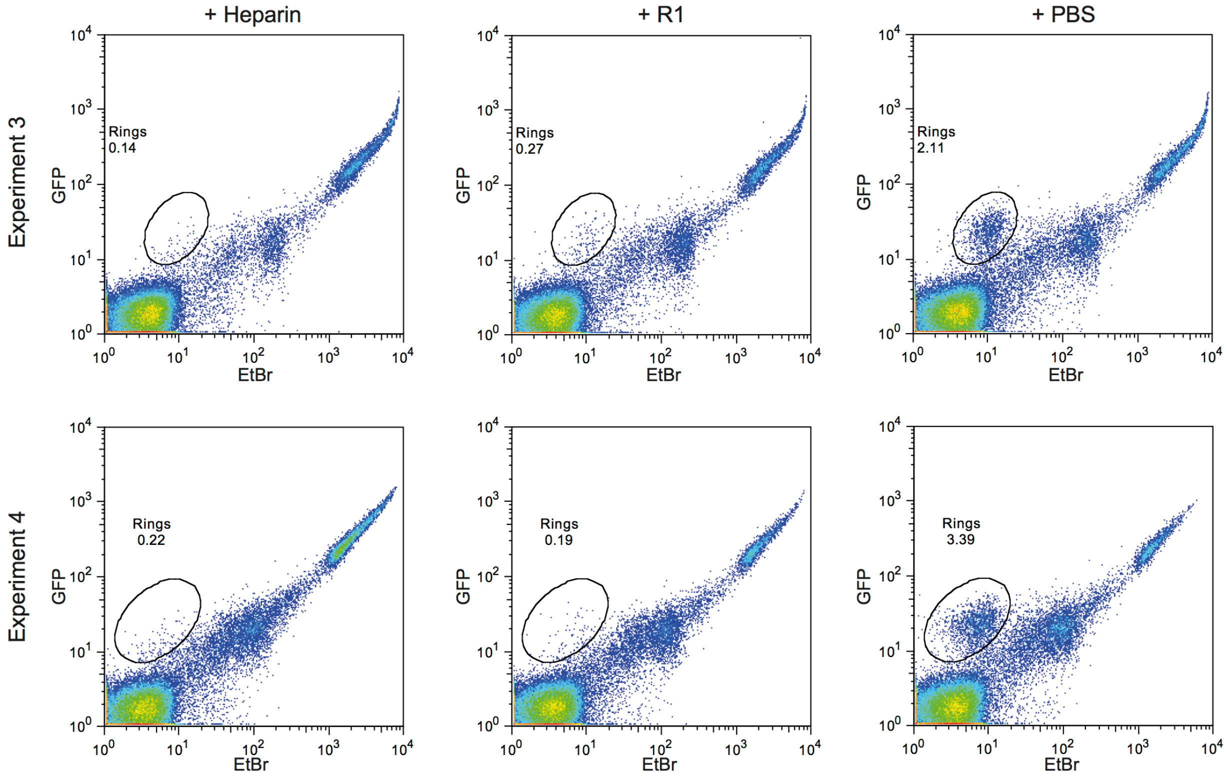

Zuccala and Satchwell Figure S4

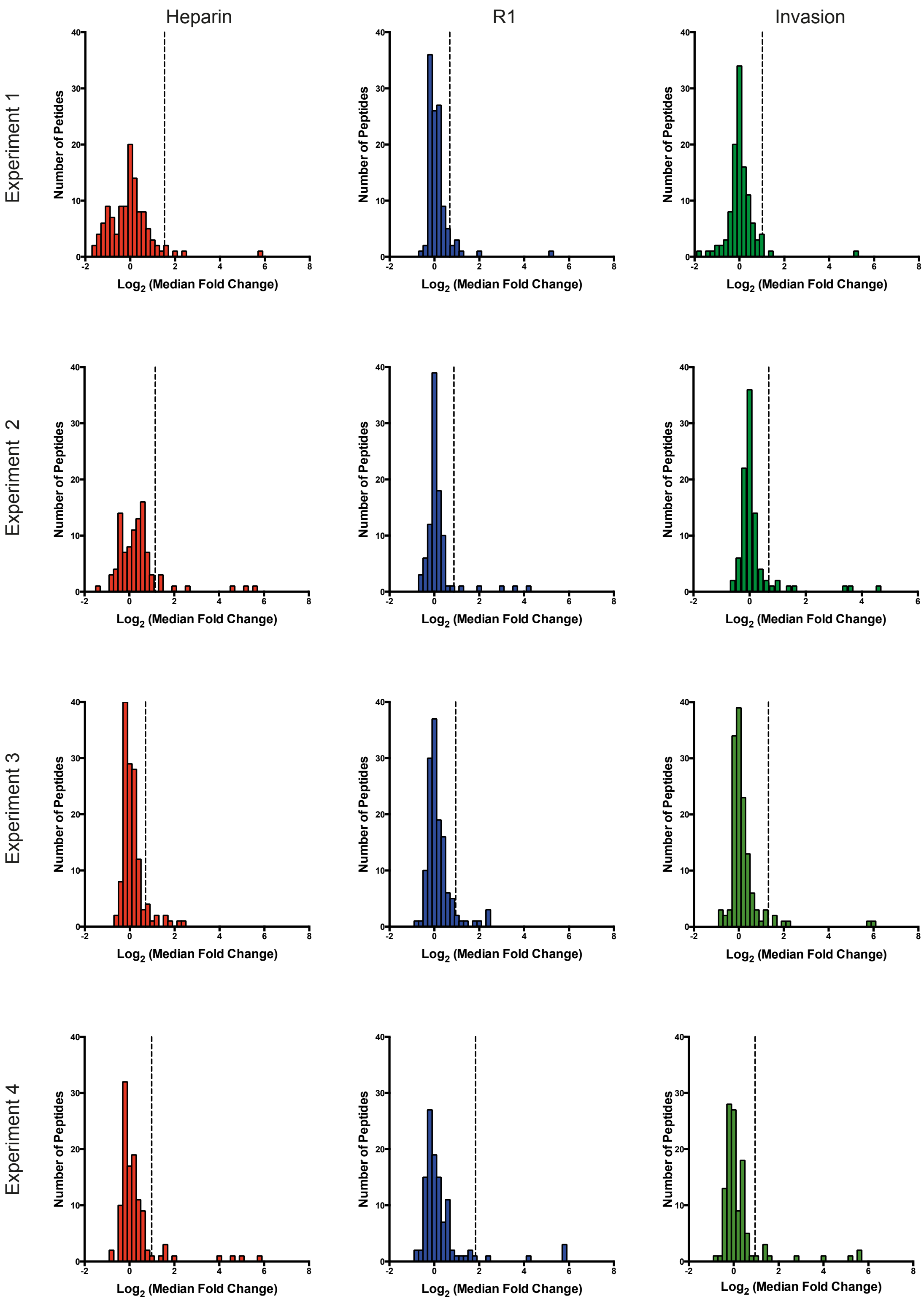

Zuccala and Satchwell Figure S5

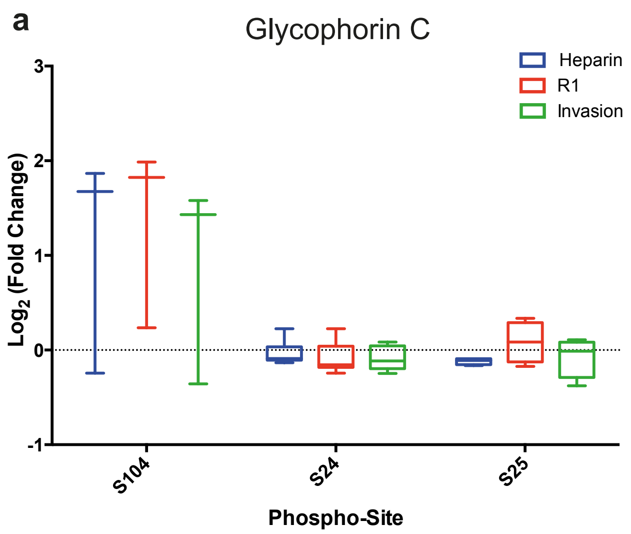

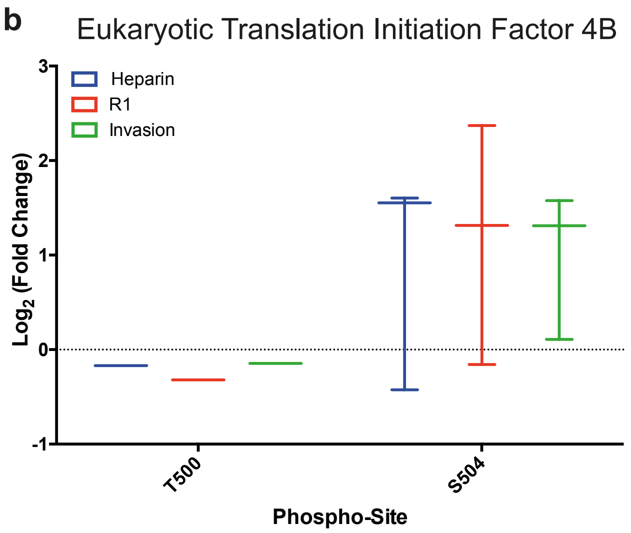

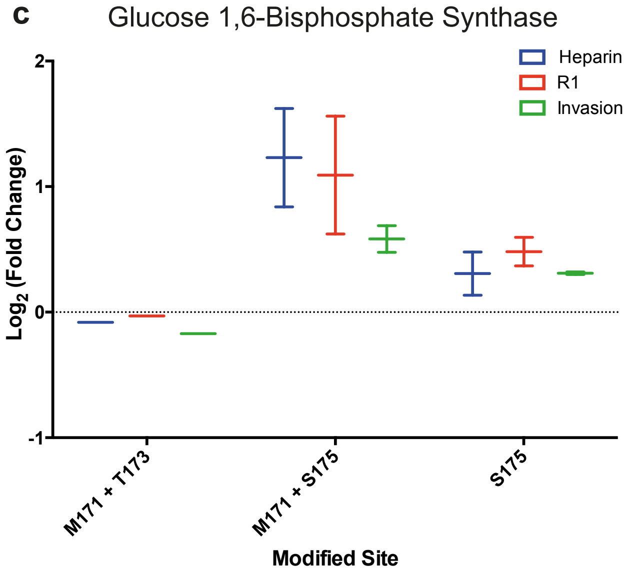

Zuccala and Satchwell Figure S6

a

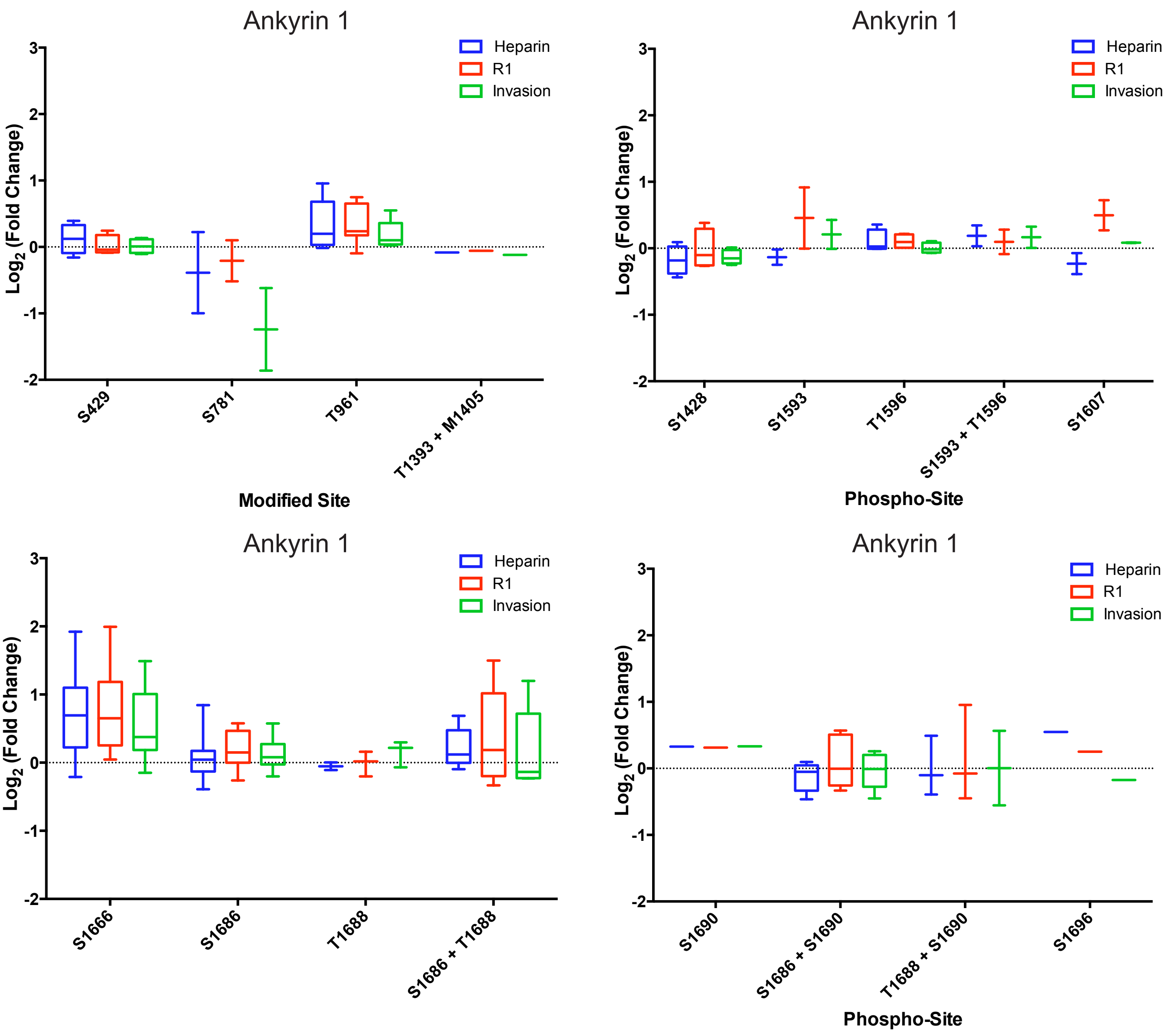

b

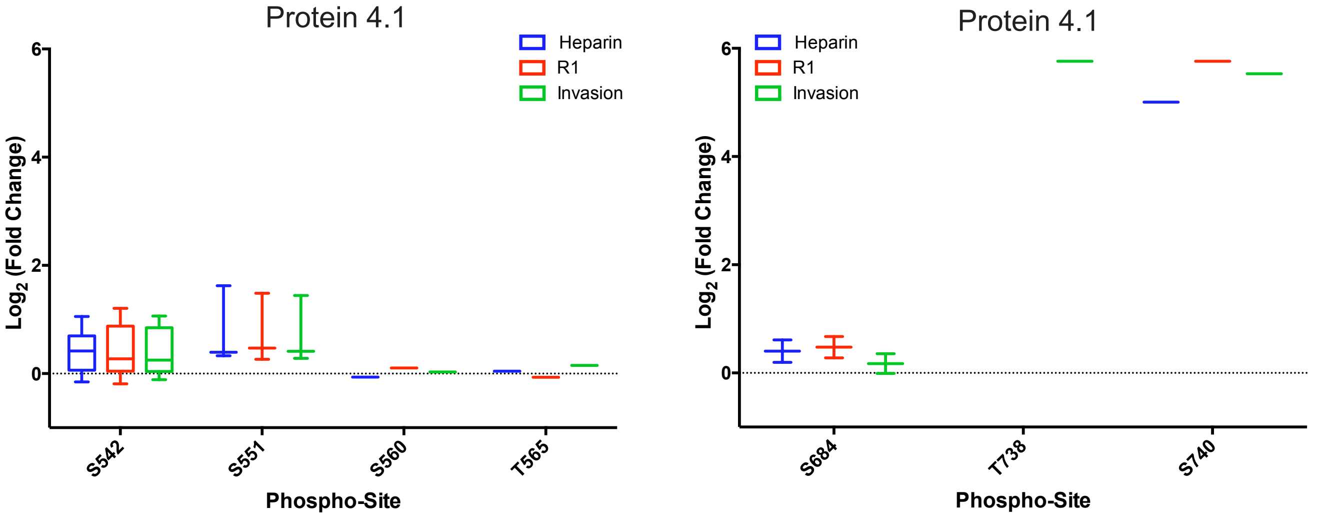

Zuccala and Satchwell Figure S7

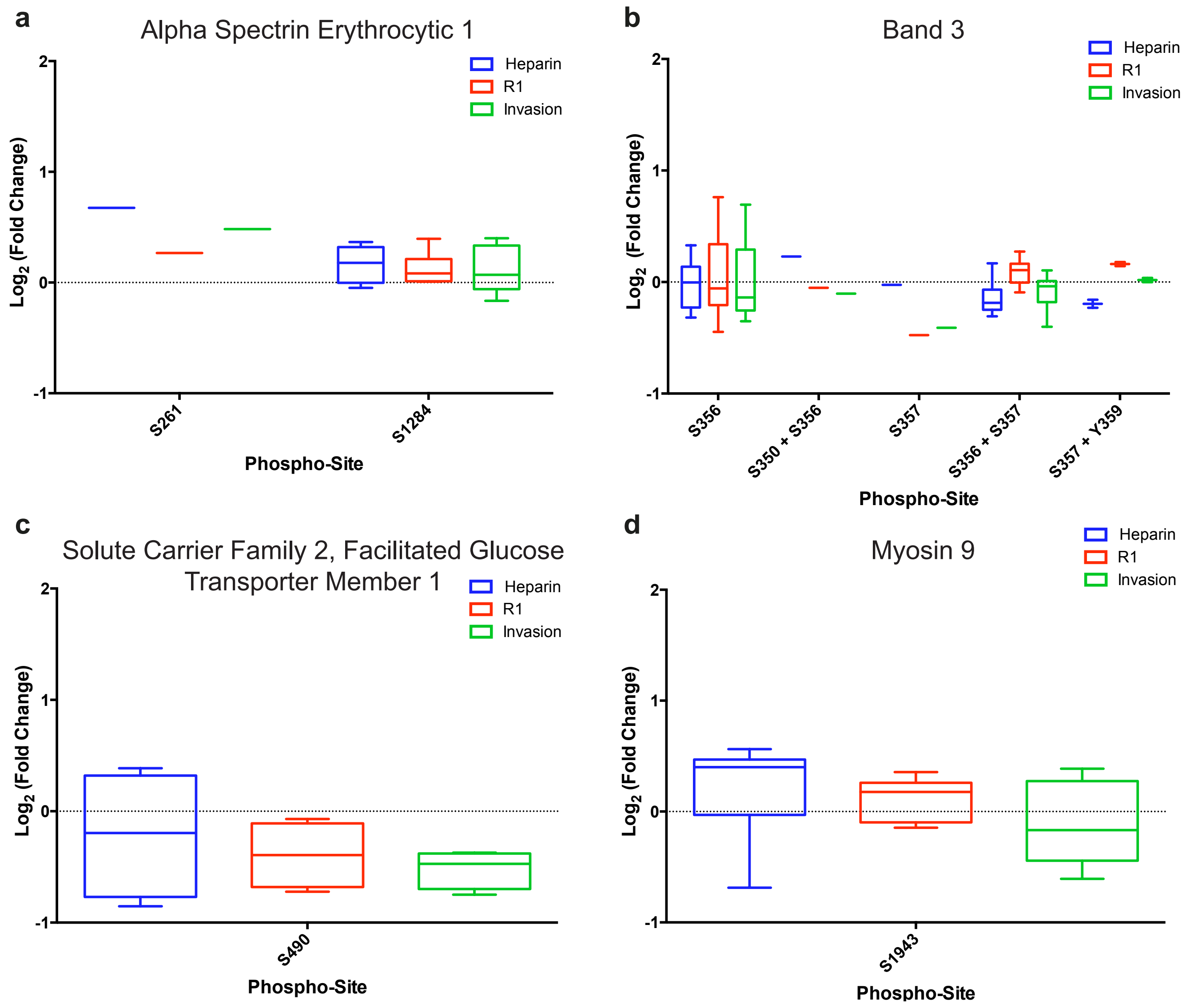

Zuccala and Satchwell Figure S8

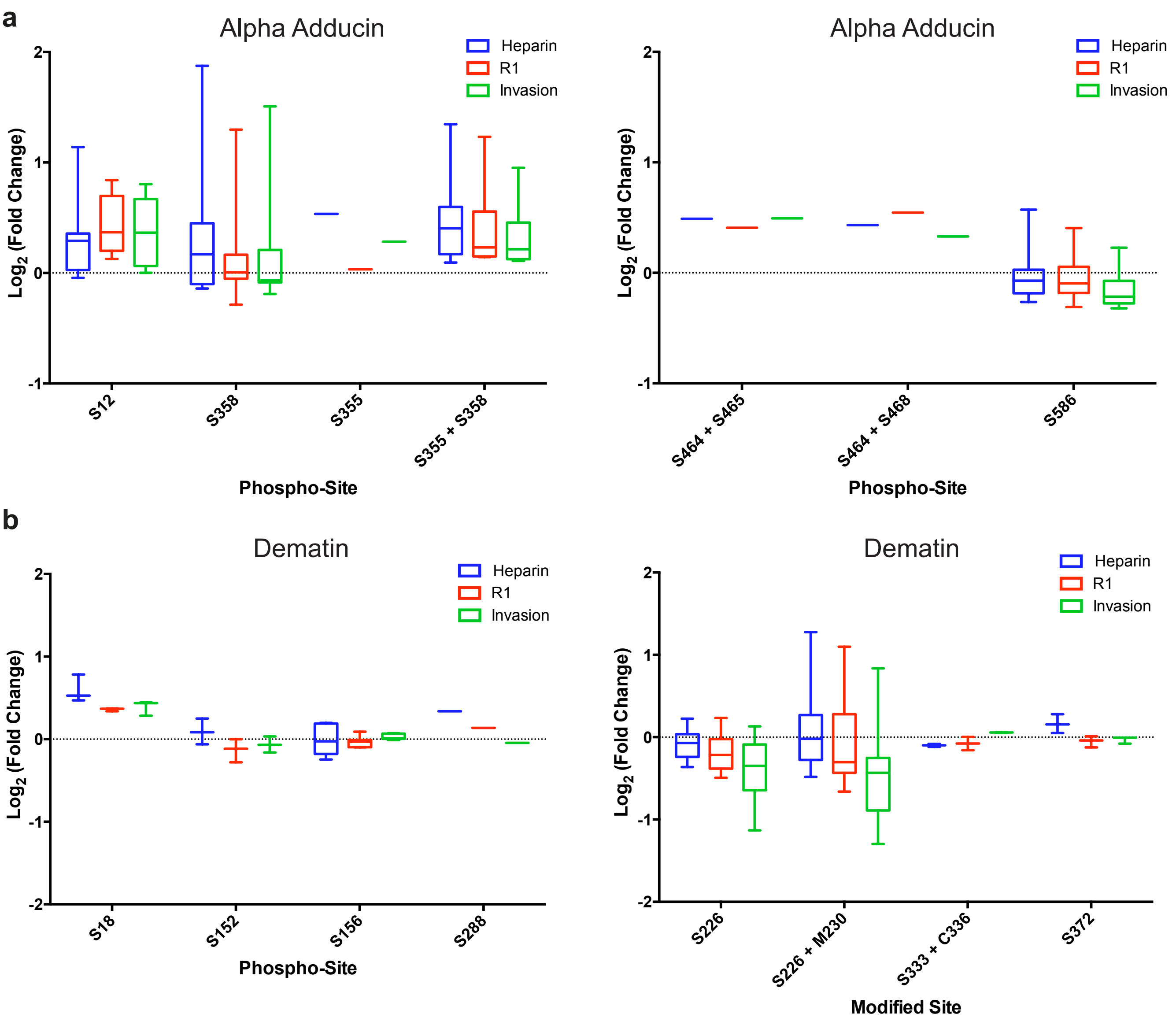

Supplement: Supplementary Information [file srep19766-s1.pdf]
